# Synthesis and Evaluation of New 1,5-Diaryl-3-[4-(methyl-sulfonyl)phenyl]-4,5-dihydro-1*H*-pyrazole Derivatives as Potential Antidepressant Agents

**DOI:** 10.3390/molecules20022668

**Published:** 2015-02-04

**Authors:** Ahmet Özdemir, Mehlika Dilek Altıntop, Zafer Asım Kaplancıklı, Özgür Devrim Can, Ümide Demir Özkay, Gülhan Turan-Zitouni

**Affiliations:** 1 Department of Pharmaceutical Chemistry, Faculty of Pharmacy, Anadolu University, 26470 Eskişehir, Turkey; E-Mails: mdaltintop@anadolu.edu.tr (M.D.A.); zakaplan@anadolu.edu.tr (Z.A.K.); gturan@anadolu.edu.tr (G.T.-Z.); 2 Graduate School of Health Sciences, Anadolu University, 26470 Eskişehir, Turkey; 3 Department of Pharmacology, Faculty of Pharmacy, Anadolu University, 26470 Eskişehir, Turkey; E-Mails: ozgurdt@anadolu.edu.tr (Ö.D.C.); udemir@anadolu.edu.tr (Ü.D.Ö.)

**Keywords:** pyrazoline, antidepressant activity, tail suspension test, modified forced swimming test, activity cage

## Abstract

In an effort to develop potent antidepressant agents, new pyrazoline derivatives **2a**–**s** were synthesized and evaluated for their antidepressant-like activity by tail suspension test (TST) and modified forced swimming test (MFST). The effects of the compounds on spontaneous locomotor activity were also investigated using an activity cage apparatus. Among these derivatives, compounds **2b**, **2d**, **2f**, **2o**, and **2r** decreased both horizontal and vertical activity number of the mice. On the other hand, compounds **2a**, **2h**, **2j**, **2k**, **2l**, **2m**, and **2n**, which did not induce any significant change in the locomotor activity, significantly shortened the immobility time of mice in TST and MFST, representing the presence of the antidepressant-like effect. Additionally, the same compounds increased the swimming time of mice in MFST without any change in climbing duration, similar to the reference drug fluoxetine (10 mg/kg). In the light of previous papers examining the effects of pyrazolines on central nervous system, this study, once more, pointed out remarkable antidepressant activity potential of pyrazoline derivatives.

## 1. Introduction

Major depressive disorder (MDD) is a multifactorial mood disorder affecting millions of people around the world. MDD typically arises in the third decade of life, with a high recurrence rate. Lifetime prevalence of MDD is around 13% and an incidence rate of 4%. Approximately 15% of patients with depression die as a consequence of the illness and MDD accounts for at least 90% of all suicides. The total cost of depression in Europe has been estimated at €118 billion [1].

According to the World Health Organization (WHO), depression is the second leading cause of disability (years of healthy life lost) in patients aged 15–44 years. For 2020, the WHO estimates that depression will become the second leading cause of disability for all age groups. By 2030, depression is expected to become the leading cause of disability in industrialized countries [1,2].

Antidepressant drugs, which increase the levels of one or more monoamines in the synaptic clefts, can be classified as tricyclic antidepressants (amitriptyline, nortriptyline, imipramine, *etc.*), monoamine oxidase inhibitors (phenelzine, moclobemide, *etc.*), selective serotonin reuptake inhibitors (fluoxetine, paroxetine, citalopram, *etc.*), selective noradrenaline reuptake inhibitors (reboxetine), and serotonin-noradrenaline reuptake inhibitors (venlafaxine, desvenlafaxine) [1,2,3,4,5,6].

The limited mechanistic understanding of depression pathogenesis and decreased antidepressant treatment response have resulted in the high rate of treatment failures. As a result, pharmaceutical industry has focused on delineating the mechanisms underlying depression as well as on the antidepressant drug discovery [3,4,5,6].

Hydrazine-based drugs still remain in clinical use for the treatment of depression. Due to their side effects, medicinal chemists have focused on the discovery of new antidepressant agents with enhanced pharmacological activity and limited toxicity *via* the structural modification of the hydrazine group [7].

In medicinal chemistry, pyrazoline scaffold has attracted a great deal of interest owing to its high synthetic accessibility and diverse therapeutic applications. In particular, pyrazolines are considered as the cyclic congeners of hydrazine group and therefore considerable research on them in relation to depression has been carried out [7,8,9,10,11,12,13,14,15,16,17]. Encouraged by the large number of papers regarding the antidepressant potential of pyrazoline scaffold [7,8,9,10,11,12,13,14,15,16,17], herein we describe the synthesis and *in vivo* evaluation of some new methylsulfonyl-substituted pyrazoline derivatives as potential antidepressant agents.

## 2. Results and Discussion

**Scheme 1:**
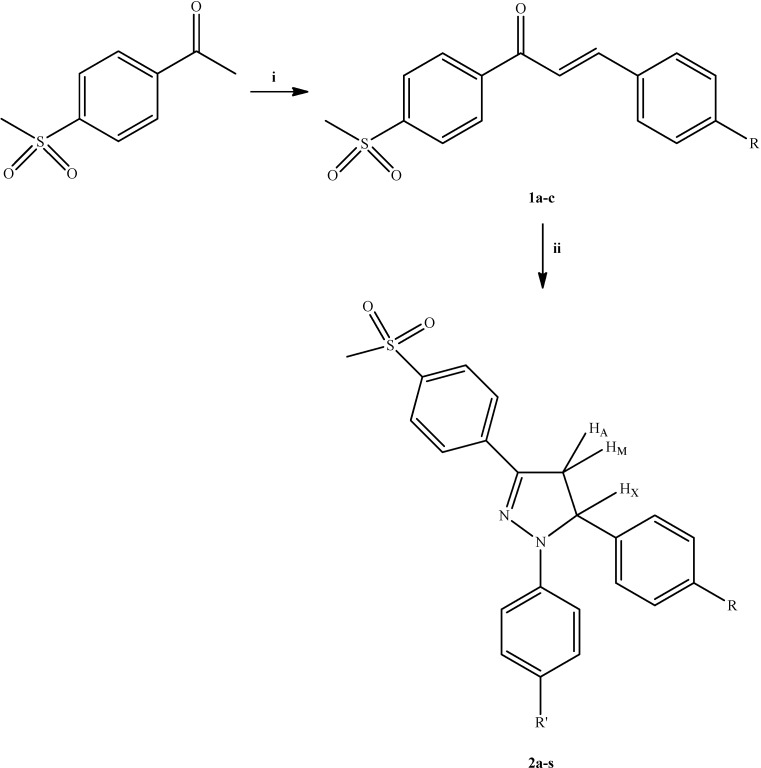
The synthetic route for the preparation of compounds **2a**–**s**.

The synthesis of compounds **2a**–**s** followed the general pathway outlined in Scheme 1. Methylsulfonyl-substituted chalcones **1a**–**c** were synthesized *via* the base-catalyzed Claisen-Schmidt condensation of 4'-(methylsulfonyl)acetophenone with appropriate aromatic aldehydes [18]. The ring closure reaction of chalcones **1a**–**c** with phenylhydrazine hydrochloride derivatives in hot acetic acid afforded 1,5-diaryl-3-[4-(methylsulfonyl)phenyl]-4,5-dihydro-1*H*-pyrazoles **2a**–**s**.

*Reagents and conditions*: (i) aromatic aldehyde, 10% aqueous sodium hydroxide solution, ethanol, rt, 10 h; (ii) appropriate phenylhydrazine hydrochloride derivative, CH_3_COOH, reflux, 8 h.

The structures of the newly synthesized compounds were elucidated by FT-IR, ^1^H-NMR, ^13^C-NMR, mass spectral data, and elemental analyses. In the IR spectra of compounds **2a**–**s**, C=N and C=C stretching bands were observed in the region 1596–1406 cm^−1^. In the ^1^H-NMR spectra of compounds **2a**–**s**, the CH_2_ protons of the pyrazoline ring resonated as a pair of doublets of doublets at δ 3.13–3.20 ppm (*J_AM_* = 17.5–18.0 Hz, *J_AX_* = 6.0–7.5 Hz) and 3.89–3.96 ppm (*J_MA_* =16.5–18.0 Hz, *J_MX_* = 12.0–13.0 Hz). The CH proton appeared as doublet of doublets at δ 5.54–5.65 ppm (*J_MX_* =11.0–12.5 Hz, *J_AX_* = 5.5–7.0 Hz) due to the vicinal coupling with two magnetically non-equivalent protons of the methylene group at position 4 of the pyrazoline ring. All the other aromatic and aliphatic protons were observed at expected regions. The ^13^C-NMR chemical shift values of the carbon atoms at 43–44 ppm (C-4), 62–64 ppm (C-5) and 144–155 ppm (C-3) corroborate the 2-pyrazoline character deduced from the ^1^H-NMR data. In the mass spectra of compounds **2a**–**s**, the M+1 peak is observed. All compounds gave satisfactory elemental analysis.

Tail suspension test (TST) and modified forced swimming test (MFST) were carried out to evaluate the antidepressant-like effects of the test compounds. Further, the effects of the test compounds on spontaneous locomotor activity of mice were assessed by activity cage measurements.

**Figure 1:**
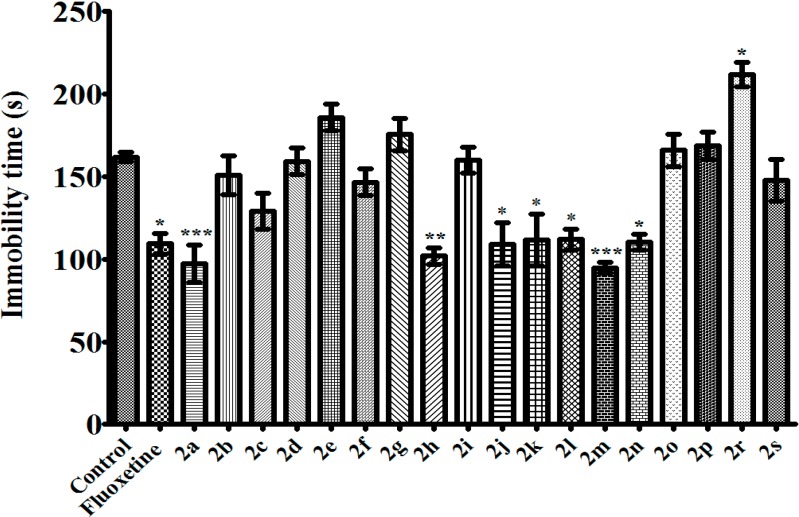
Effects of test compounds (100 mg/kg) and fluoxetine (10 mg/kg) on immobility time of mice in TST. Significance against control values * *p* < 0.05, ** *p* < 0.01, *** *p* < 0.001. Values are given as mean ± SEM. One-way ANOVA, *post-hoc* Tukey’s test, *n* = 7.

**Figure 2:**
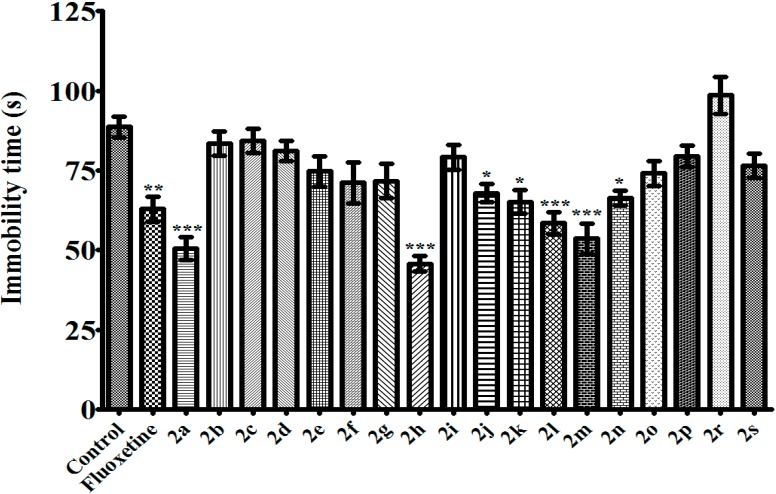
Effects of test compounds (100 mg/kg) and fluoxetine (10 mg/kg) on immobility time of mice in MFST. Significance against control values * *p* < 0.05, ** *p* < 0.01, *** *p* < 0.001. Values are given as mean ± SEM. One-way ANOVA, *post-hoc* Tukey’s test, *n* = 7.

**Figure 3:**
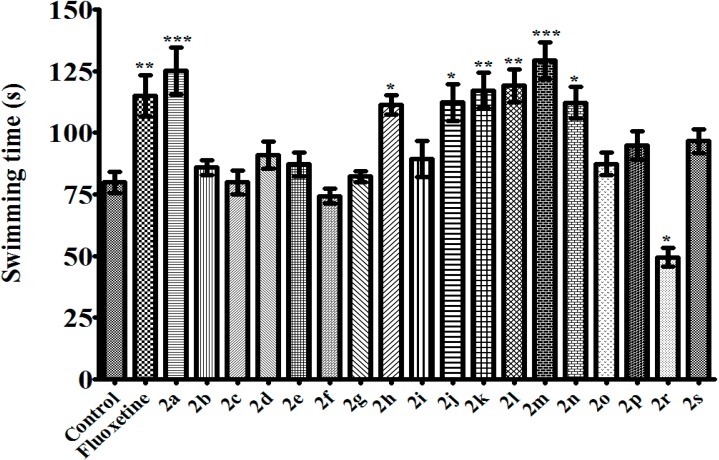
Effects of test compounds (100 mg/kg) and fluoxetine (10 mg/kg) on swimming time of mice in MFST. Significance against control values * *p* < 0.05, ** *p* < 0.01, *** *p* < 0.001. Values are given as mean ± SEM. One-way ANOVA, *post-hoc* Tukey’s test, *n* = 7.

**Figure 4:**
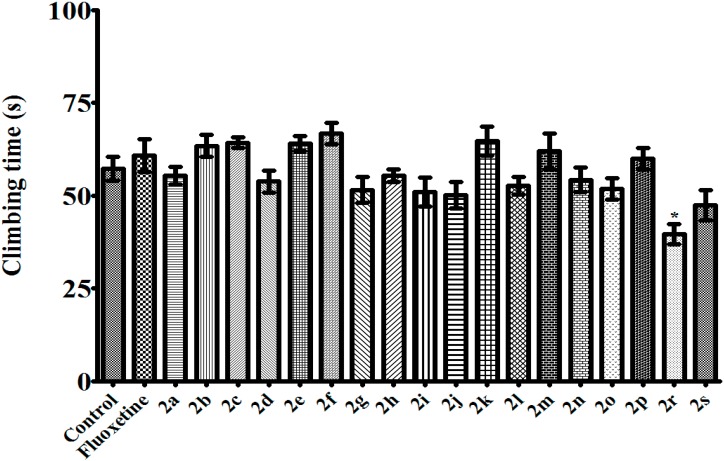
Effects of test compounds (100 mg/kg) and fluoxetine (10 mg/kg) on climbing time of mice in MFST. Significance against control values * *p* < 0.05. Values are given as mean ± SEM. One-way ANOVA, *post-hoc* Tukey’s test, *n* = 7.

TST and MFST are the most common experimental models for antidepressant activity screening. Both of these two methods based on the observation that mice, after initial escape-oriented movements, develop an immobile posture when placed in a short-term inescapable stressful situation. This immobility, referred to as behavioral despair in animals, is believed to reproduce a condition similar to human depression. Thus, a reduction in the total duration of immobility indicates an antidepressant effect [19,20,21]. In this study, when assessed in TST and MFST, compounds **2a**, **2h**, **2j**, **2k**, **2l**, **2m**, and **2n** decreased the immobility time of mice compared to the control group, indicating the antidepressant-like effects of these pyrazoline derivatives (100 mg/kg) (Figure 1 and Figure 2). In MFST, the same compounds increased the swimming time of the animals without any significant change in the climbing duration (Figure 3 and Figure 4). Shortened immobility and prolonged swimming duration, without any change in the climbing time, indicated that the antidepressant-like effects of the compounds may be related to serotonergic, rather than noradrenergic mechanisms in the central nervous system [22]. Nevertheless, involvement of serotonergic system in the observed antidepressant activity must be confirmed with further studies such as depleting neuronal serotonin by *p*-chlorophenylalanine pretreatment or measuring serotonin levels in limbic areas of brain *etc.* Fluoxetine (10 mg/kg), a selective serotonin reuptake inhibitor, also showed an antidepressant-like action in both of these tests, as expected.

**Figure 5:**
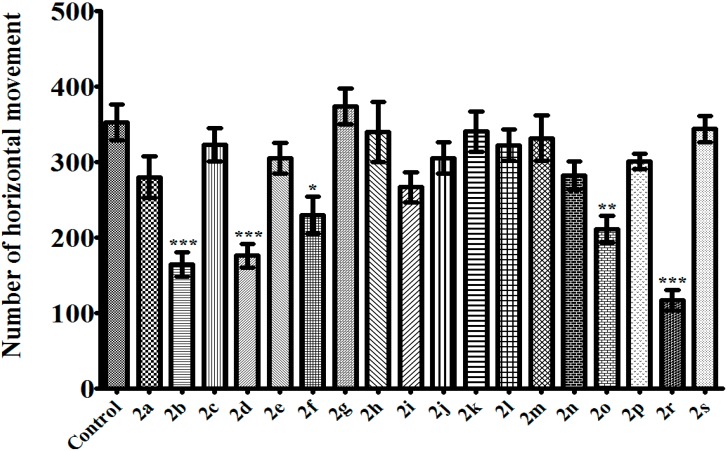
Effects of test compounds (100 mg/kg) on the number of horizontal movement of mice in the activity cage test. Significance against control values * *p* < 0.05, ** *p* < 0.01, *** *p* < 0.001. Values are given as mean ± SEM. One-way ANOVA, *post-hoc* Tukey’s test, *n* = 7.

**Figure 6:**
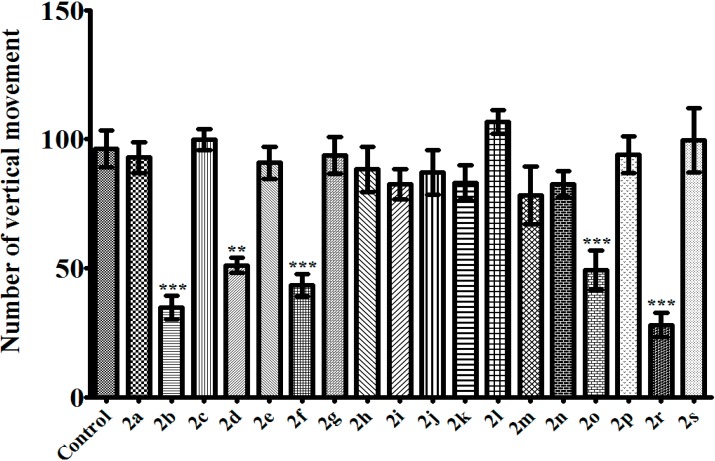
Effects of test compounds (100 mg/kg) on the number of vertical movement of mice in the activity cage test. Significance against control values ** *p* < 0.01, *** *p* < 0.001. Values are given as mean ± SEM. One-way ANOVA, *post-hoc* Tukey’s test, *n* = 7.

In the activity cage test, compounds **2a**, **2h**, **2j**, **2k**, **2l**, **2m**, and **2n** possessing antidepressant-like activity did not induce any significant alteration in the total number of spontaneous locomotor activities (Figure 5 and Figure 6). This means that the anti-immobility effect cannot be attributable to a stimulant activity. In other words, the observed antidepressant-like effect is specific. On the other hand, compounds **2b**, **2d**, **2f**, and **2o**, which did not induce any alteration in the immobility or the swimming time of the animals, significantly reduced the number of both horizontal and vertical locomotor activity (Figure 5 and Figure 6). This decrease in the spontaneous locomotor activity may be produced by neurosedative effect of these aforementioned compounds. Instead, the effects of these compounds on neuromuscular junction may also cause this situation. Further detailed studies will help to clarify this issue.

Among the tested pyrazoline derivatives, compound **2r** was the only compound increasing the immobility time of the mice in TST (Figure 1). On the other hand, the same compound did not change the immobility time in MFST (Figure 2). Furthermore, in MFST, it decreased both swimming and climbing time of the mice (Figure 3 and Figure 4). Therefore, the prolongation of the immobility time in TST, may not be caused by a possible depressant-like activity of compound **2r**; instead this compound probably affected motor activity/motor coordination of the mice. As a matter of fact, in the activity cage test, decrease in the number of spontaneous locomotor activity of **2r**-treated animals (Figure 4 and Figure 5) confirmed this idea. However, examining motor coordination of the animals by a further experiment such as a Rota-rod test, may provide additional information about the unexpected immobility-inducing effect of compound **2r** in the TST.

As well as their remarkable antidepressant-like activity, compounds **2a**, **2h**, **2j**, **2k**, **2l**, **2m**, and **2n** exhibited negligible toxicity; incurred neither deaths nor undesirable side effects such as ataxia, paralysis, convulsions, and diarrhea, giving an idea about the safety of the compounds. However, the exact mechanism of action and probable side effects of these compounds should be clarify with further detailed studies.

## 3. Experimental Section

### 3.1. General Information

All reagents were purchased from commercial suppliers and were used without further purification. Melting points were determined on an Electrothermal 9100 melting point apparatus (Weiss-Gallenkamp, Loughborough, UK) and were uncorrected. IR spectra were recorded on a Shimadzu 8400 FT-IR spectrophotometer (Shimadzu, Tokyo, Japan). ^1^H-NMR and ^13^C-NMR spectra were recorded on a Bruker spectrometer (Bruker, Billerica, MA, USA). Mass spectra were recorded on an Agilent LC-MSD-Trap-SL Mass spectrometer (Agilent Technologies, Palo Alto, CA, USA). Elemental analyses were performed on a Perkin Elmer EAL 240 elemental analyzer (Perkin-Elmer, Norwalk, CT, USA). Thin Layer Chromatography (TLC) was performed on TLC Silica gel 60 F_254_ aluminium sheets (Merck, Darmstadt, Germany) using petroleum ether-ethyl acetate (3:1 v/v) as eluent.

### 3.2. Chemistry: General Procedures for the Synthesis of Compounds

#### 3.2.1. 3-(4-Substituted phenyl)-1-[4-(methylsulfonyl)phenyl]-2-propen-1-ones **1a**–**c**

A mixture of 4'-(methylsulfonyl)acetophenone (0.05 mol), aromatic aldehyde (0.05 mol) and 10% aqueous sodium hydroxide (10 mL) in ethanol (30 mL) was stirred at room temperature for 10 h. The progress of the reaction was checked by TLC. Upon completion, the reaction mixture was poured into crushed ice. The precipitated solid was filtered, washed with water, and dried. The product was crystallized from ethanol [18].

#### 3.2.2. 1,5-Diaryl-3-[4-(methylsulfonyl)phenyl]-4,5-dihydro-1*H*-pyrazoles **2a**–**s**

A mixture of the appropriate chalcone **1a**–**c** (10.0 mmol) and phenylhydrazine hydrochloride derivative (20.0 mmol) in the presence of acetic acid (50 mL) was refluxed for 8 h, then poured into crushed ice. The precipitate was separated by filtration, washed with water and crystallized from methanol.

*1-(4-Chlorophenyl)-5-(4-fluorophenyl)-3-(4-(methylsulfonyl)phenyl)-4,5-dihydro-1H-pyrazole* (**2a**): Yield: 93%; m.p. 193 °C. IR (KBr) ν_max_ (cm^−1^): 3012.60 (Aromatic C-H), 2918.10 (Aliphatic C-H), 1585.38, 1490.87 (C=N and C=C), 1307.65, 1149.50, 1087.78 (SO_2_ and C-N), 835.12 (C-H out of plane deformation). ^1^H-NMR (500 MHz, DMSO-*d*_6_) δ (ppm): 3.19 (1H, dd, *J_AM_* = 17.5 Hz, *J_AX_* = 6.0 Hz, C_4_-H_A_ pyrazoline), 3.23 (3H, s, SO_2_CH_3_), 3.96 (1H, dd, *J_MA_* = 17.5 Hz, *J_MX_* = 12.0 Hz, C_4_-H_M_ pyrazoline), 5.65 (1H, dd, *J_MX_* = 12.5 Hz, *J_AX_* = 6.0 Hz, C_5_-H_X_ pyrazoline), 7.04 (2H, d, *J* = 9.0 Hz, aromatic protons), 7.15–7.19 (2H, m, aromatic protons), 7.23 (2H, d, *J* = 9.0 Hz, aromatic protons), 7.29–7.32 (2H, m, aromatic protons), 7.94–8.02 (4H, m, 4-methylsulfonylphenyl protons). ^13^C-NMR (125 MHz, DMSO-*d*_6_) δ (ppm): 42.52 (CH_3_), 43.51 (CH_2_), 62.59 (CH), 114.74 (2CH), 115.78 (CH), 115.95 (CH), 126.28 (C), 127.33 (2CH), 127.98 (CH), 128.77 (2CH), 131.53 (CH), 136.72 (CH), 137.77 (CH), 140.12 (C), 142.19 (C), 144.05 (C), 146.46 (C), 152.05 (C), 160.46 (C). Anal. Calcd. for C_22_H_18_ClFN_2_O_2_S: C, 61.61; H, 4.23; N, 6.53; Found: C, 61.60; H, 4.25; N, 6.52. MS (ESI) (*m/z*): [M+1]^+^ 429.

*1,5-Bis(4-fluorophenyl)-3-(4-(methylsulfonyl)phenyl)-4,5-dihydro-1H-pyrazole* (**2b**): Yield: 85%; m.p. 221 °C. IR (KBr) ν_max_ (cm^−1^): 1508.23 (C=N), 1305.72, 1228.57, 1151.42 (SO_2_ and C-N), 835.12 (C-H out of plane deformation). ^1^H-NMR (500 MHz, DMSO-*d*_6_) δ (ppm): 3.18 (1H, dd, *J_AM_* = 18.0 Hz, *J_AX_* = 7.0 Hz, C_4_-H_A_ pyrazoline), 3.23 (3H, s, SO_2_CH_3_), 3.94 (1H, dd, *J_MA_* = 17.5 Hz, *J_MX_* = 12.5 Hz, C_4_-H_M_ pyrazoline), 5.61 (1H, dd, *J_MX_* = 12.5 Hz, *J_AX_* = 6.5 Hz, C_5_-H_X_ pyrazoline), 7.04 (4H, d, *J* = 6.5 Hz, aromatic protons), 7.15–7.19 (2H, m, aromatic protons), 7.31–7.34 (2H, m, aromatic protons), 7.93–7.97 (4H, m, 4-methylsulfonylphenyl protons). ^13^C-NMR (125 MHz, DMSO-*d*_6_) δ (ppm): 42.54 (CH_3_), 43.53 (CH_2_), 63.13 (CH), 114.54 (CH), 114.60 (CH), 115.46 (CH), 115.76 (CH), 115.93 (CH), 126.14 (2CH), 127.33 (2CH), 128.00 (CH), 128.07 (CH), 136.92 (CH), 138.01 (C), 139.92 (C), 140.25 (C), 145.75 (C), 155.34 (C), 160.45 (C), 162.39 (C). Anal. Calcd. for C_22_H_18_F_2_N_2_O_2_S: C, 64.06; H, 4.40; N, 6.79; Found: C, 64.05; H, 4.39; N, 6.80. MS (ESI) (*m/z*): [M+1]^+^ 413.

*1-(4-Bromophenyl)-5-(4-fluorophenyl)-3-(4-(methylsulfonyl)phenyl)-4,5-dihydro-1H-pyrazole* (**2c**): Yield: 77%; m.p. 169 °C. IR (KBr) ν_max_ (cm^−1^): 3014.53 (Aromatic C-H), 1583.45, 1488.94 (C=N and C=C), 1307.65, 1151.42, 1085.85 (SO_2_ and C-N), 835.12 (C-H out of plane deformation). ^1^H-NMR (500 MHz, DMSO-*d*_6_) δ (ppm): 3.19 (1H, dd, *J_AM_* = 17.5 Hz, *J_AX_* = 6.0 Hz, C_4_-H_A_ pyrazoline), 3.23 (3H, s, SO_2_CH_3_), 3.95 (1H, dd, *J_MA_* = 17.5 Hz, *J_MX_* = 12.5 Hz, C_4_-H_M_ pyrazoline), 5.65 (1H, dd, *J_MX_* = 12.5 Hz, *J_AX_* = 6.0 Hz, C_5_-H_X_ pyrazoline), 6.99 (2H, d, *J* = 9.0 Hz, aromatic protons), 7.15–7.19 (2H, m, aromatic protons), 7.29–7.32 (2H, m, aromatic protons), 7.33–7.36 (2H, m, aromatic protons), 7.94–7.98 (4H, m, 4-methylsulfonylphenyl protons). ^13^C-NMR (125 MHz, DMSO-*d*_6_) δ (ppm): 42.52 (CH_3_), 43.51 (CH_2_), 62.49 (CH), 115.22 (2CH), 115.80 (CH), 115.97 (CH), 125.98 (2CH), 127.35 (2CH), 127.97 (CH), 129.36 (C), 131.62 (2CH), 136.71 (CH), 137.73 (C), 140.14 (C), 142.52 (C), 146.56 (C), 160.47 (C), 162.41 (C). Anal. Calcd. for C_22_H_18_BrFN_2_O_2_S: C, 55.82; H, 3.83; N, 5.92. Found: C, 55.80; H, 3.81; N, 5.92. MS (ESI) (*m/z*): [M+1]^+^ 474.

*1-(4-Methoxyphenyl)-5-(4-fluorophenyl)-3-(4-(methylsulfonyl)phenyl)-4,5-dihydro-1H-pyrazole* (**2d**): Yield: 60%; m.p. 170 °C. IR (KBr) ν_max_ (cm^−1^): 1510.16 (C=N), 1321.15, 1245.93, 1153.35, 1099.35 (SO_2_, C-N and C-O), 811.98 (C-H out of plane deformation). ^1^H-NMR (500 MHz, DMSO-*d*_6_) δ (ppm): 3.13 (1H, dd, *J_AM_* = 17.5 Hz, *J_AX_* = 7.0 Hz, C_4_-H_A_ pyrazoline), 3.22 (3H, s, SO_2_CH_3_), 3.65 (3H, s, OCH_3_), 3.89 (1H, dd, *J_MA_* = 17.5 Hz, *J_MX_* = 12.5 Hz, C_4_-H_M_ pyrazoline), 5.54 (1H, dd, *J_MX_* = 12.5 Hz, *J_AX_* = 7.0 Hz, C_5_-H_X_ pyrazoline), 6.80 (2H, d, *J* = 7.0 Hz, aromatic protons), 6.99 (2H, d, *J* = 7.0 Hz, aromatic protons), 7.14–7.18 (2H, m, aromatic protons), 7.31–7.34 (2H, m, aromatic protons), 7.90–7.94 (4H, m, 4-methylsulfonylphenyl protons). ^13^C-NMR (125 MHz, DMSO-*d*_6_) δ (ppm): 42.35 (CH_3_), 43.56 (CH_2_), 55.16 (CH_3_), 63.54 (CH), 114.41 (2CH), 114.78 (2CH), 115.67 (CH), 115.84 (CH), 125.85 (2CH), 127.32 (2CH), 128.05 (CH), 128.11 (CH), 137.19 (C), 138.31 (C), 139.51 (C), 144.53 (C), 153.18 (C), 160.41 (C), 162.34 (C). Anal. Calcd. for C_23_H_21_FN_2_O_3_S: C, 65.08; H, 4.99; N, 6.60. Found: C, 65.10; H, 4.97; N, 6.59. MS (ESI) (*m/z*): [M+1]^+^ 425.

*1-(4-Methylphenyl)-5-(4-fluorophenyl)-3-(4-(methylsulfonyl)phenyl)-4,5-dihydro-1H-pyrazole* (**2e**): Yield: 76%; m.p. 186 °C. IR (KBr) ν_max_ (cm^−1^): 3028.03 (Aromatic C-H), 2920.03 (Aliphatic C-H), 1585.38, 1510.16 (C=N and C=C), 1305.72, 1224.71, 1151.42, 1087.78 (SO_2_ and C-N), 835.12, 781.12 (C-H out of plane deformation). ^1^H-NMR (500 MHz, DMSO-*d*_6_) δ (ppm): 2.17 (3H, s, CH_3_), 3.14 (1H, dd, *J_AM_* = 17.5 Hz, *J_AX_* = 6.0 Hz, C_4_-H_A_ pyrazoline), 3.22 (3H, s, SO_2_CH_3_), 3.91 (1H, dd, *J_MA_* = 17.5 Hz, *J_MX_* = 12.5 Hz, C_4_-H_M_ pyrazoline), 5.60 (1H, dd, *J_MX_* = 12.0 Hz, *J_AX_* = 6.0 Hz, C_5_-H_X_ pyrazoline), 6.95–7.00 (4H, m, aromatic protons), 7.13–7.18 (2H, m, aromatic protons), 7.28–7.35 (2H, m, aromatic protons), 7.91–7.94 (4H, m, 4-methylsulfonylphenyl protons). ^13^C-NMR (125 MHz, DMSO-*d*_6_) δ (ppm): 20.08 (CH_3_), 43.20 (CH_3_), 43.56 (CH_2_), 62.88 (CH), 113.45 (2CH), 115.68 (CH), 115.85 (CH), 115.91 (CH), 125.97 (2CH), 127.38 (2CH), 128.00 (CH), 128.22 (CH), 129.41 (CH), 131.55 (C), 137.11 (C), 139.66 (C), 144.07 (C), 144.89 (C), 160.39 (C), 162.33 (C). Anal. Calcd. for C_23_H_21_FN_2_O_2_S: C, 67.63; H, 5.18; N, 6.86. Found: C, 67.65; H, 5.17; N, 6.85. MS (ESI) (*m/z*): [M+1]^+^ 409.

*1-(4-Methoxyphenyl)-5-(4-chlorophenyl)-3-(4-(methylsulfonyl)phenyl)-4,5-dihydro-1H-pyrazole* (**2f**): Yield: 76%; m.p. 152 °C. IR (KBr) ν_max_ (cm^−1^): 2925.81 (Aliphatic C-H asymmetric), 2833.24 (Aliphatic C-H symmetric), 1508.23 (C=N), 1307.65, 1242.07, 1151.42, 1087.78 (SO_2_, C-N and C-O), 825.48 (C-H out of plane deformation). ^1^H-NMR (500 MHz, DMSO-*d*_6_) δ (ppm): 3.14 (1H, dd, *J_AM_* = 18.0 Hz, *J_AX_* = 7.5 Hz, C_4_-H_A_ pyrazoline), 3.22 (3H, s, SO_2_CH_3_), 3.65 (3H, s, OCH_3_), 3.90 (1H, dd, *J_MA_* = 17.0 Hz, *J_MX_* = 12.5 Hz, C_4_-H_M_ pyrazoline), 5.55 (1H, dd, *J_MX_* = 12.5 Hz, *J_AX_* = 7.0 Hz, C_5_-H_X_ pyrazoline), 6.80 (2H, d, *J* = 9.0 Hz, aromatic protons), 6.98 (2H, d, *J* = 9.0 Hz, aromatic protons), 7.30 (2H, d, *J* = 8.5 Hz, aromatic protons), 7.39 (2H, d, *J* = 8.5 Hz, aromatic protons), 7.90–7.94 (4H, m, 4-methylsulfonylphenyl protons). ^13^C-NMR (125 MHz, DMSO-*d*_6_) δ (ppm): 42.73 (CH_3_), 44.06 (CH_2_), 55.66 (CH_3_), 64.03 (CH), 114.95 (2CH), 115.25 (2CH), 126.37 (2CH), 127.82 (2CH), 128.49 (2CH), 129.46 (2CH), 131.76 (C), 138.10 (C), 140.05 (C), 141.59 (C), 145.09 (C), 153.70 (C), 160.40 (C). Anal. Calcd. for C_23_H_21_ClN_2_O_3_S: C, 62.65; H, 4.80; N, 6.35. Found: C, 62.63; H, 4.78; N, 6.34. MS (ESI) (*m/z*): [M+1]^+^ 441.

*1-(4-Fluorophenyl)-5-(4-chlorophenyl)-3-(4-(methylsulfonyl)phenyl)-4,5-dihydro-1H-pyrazole* (**2g**): Yield: 87%; m.p. 203 °C. IR (KBr) ν_max_ (cm^−1^): 2974.03 (Aliphatic C-H asymmetric), 2885.31 (Aliphatic C-H symmetric), 1577.66, 1504.37 (C=N and C=C), 1305.72, 1230.50, 1151.42, 1087.78 (SO_2_ and C-N), 825.48 (C-H out of plane deformation). ^1^H-NMR (500 MHz, DMSO-*d*_6_) δ (ppm): 3.18 (1H, dd, *J_AM_* = 17.5 Hz, *J_AX_* = 6.5 Hz, C_4_-H_A_ pyrazoline), 3.23 (3H, s, SO_2_CH_3_), 3.95 (1H, dd, *J_MA_* = 17.5 Hz, *J_MX_* = 12.5 Hz, C_4_-H_M_ pyrazoline), 5.61 (1H, dd, *J_MX_* = 12.5 Hz, *J_AX_* = 6.5 Hz, C_5_-H_X_ pyrazoline), 7.02–7.07 (4H, m, aromatic protons), 7.30 (2H, d, *J* = 7.0 Hz, aromatic protons), 7.40 (2H, d, *J* = 6.5 Hz, aromatic protons), 7.93–7.96 (4H, m, 4-methylsulfonylphenyl protons). ^13^C-NMR (125 MHz, DMSO-*d*_6_) δ (ppm): 42.42 (CH_3_), 43.52 (CH_2_), 63.12 (CH), 114.51 (CH), 114.57 (CH), 115.50 (CH), 115.68 (CH), 126.16 (2CH), 127.34 (2CH), 127.93 (2CH), 129.04 (2CH), 132.13 (C), 136.85 (C), 139.95 (C), 140.78 (C), 145.81 (C), 155.36 (C), 160.41 (C). Anal. Calcd. for C_22_H_18_ClFN_2_O_2_S: C, 61.61; H, 4.23; N, 6.53. Found: C, 61.60; H, 4.22; N, 6.52. MS (ESI) (*m/z*): [M+1]^+^ 429

*1-(4-Methylphenyl)-5-(4-chlorophenyl)-3-(4-(methylsulfonyl)phenyl)-4,5-dihydro-1H-pyrazole* (**2h**): Yield: 83%; m.p. 230 °C. IR (KBr) ν_max_ (cm^−1^): 2970.17 (Aliphatic C-H asymmetric), 2883.38 (Aliphatic C-H symmetric), 1510.16 (C=N), 1305.72, 1151.42, 1087.78 (SO_2_ and C-N), 825.48 (C-H out of plane deformation). ^1^H-NMR (500 MHz, DMSO-*d*_6_) δ (ppm): 2.17 (3H, s, CH_3_), 3.15 (1H, dd, *J_AM_* = 17.5 Hz, *J_AX_* = 6.0 Hz, C_4_-H_A_ pyrazoline), 3.22 (3H, s, SO_2_CH_3_), 3.92 (1H, dd, *J_MA_* = 18.0 Hz, *J_MX_* = 12.0 Hz, C_4_-H_M_ pyrazoline), 5.60 (1H, dd, *J_MX_* = 12.5 Hz, *J_AX_* = 6.0 Hz, C_5_-H_X_ pyrazoline), 6.95 (2H, d, *J* = 7.0 Hz, aromatic protons), 6.99 (2H, d, *J* = 8.0 Hz, aromatic protons), 7.29 (2H, d, *J* = 7.0 Hz, aromatic protons), 7.39 (2H, d, *J* = 8.5 Hz, aromatic protons), 7.93 (4H, s, 4-methylsulfonylphenyl protons). ^13^C-NMR (125 MHz, DMSO-*d*_6_) δ (ppm): 20.07 (CH_3_), 42.15 (CH_3_), 43.55 (CH_2_), 62.87 (CH), 113.43 (2CH), 125.99 (2CH), 127.33 (2CH), 127.87 (2CH), 128.27 (CH), 128.96 (CH), 129.43 (CH), 132.00 (CH), 137.05 (C), 139.70 (C), 141.07 (C), 141.13 (2C), 144.96 (C), 160.43 (C). Anal. Calcd. for C_23_H_21_ClN_2_O_2_S: C, 65.01; H, 4.98; N, 6.59. Found: C, 65.00; H, 4.99; N, 6.58. MS (ESI) (*m/z*): [M+1]^+^ 425.

*1-(4-Bromophenyl)-5-(4-chlorophenyl)-3-(4-(methylsulfonyl)phenyl)-4,5-dihydro-1H-pyrazole* (**2i**): Yield: 83%; m.p. 204 °C. IR (KBr) ν_max_ (cm^−1^): 2918.10 (Aliphatic C-H), 1583.45, 1487.01 (C=N and C=C), 1307.65, 1151.42, 1087.78 (SO_2_ and C-N), 819.69 (C-H out of plane deformation). ^1^H-NMR (500 MHz, DMSO-*d*_6_) δ (ppm): 3.20 (1H, dd, *J_AM_* = 18.0 Hz, *J_AX_* = 6.0 Hz, C_4_-H_A_ pyrazoline), 3.23 (3H, s, SO_2_CH_3_), 3.96 (1H, dd, *J_MA_* = 17.5 Hz, *J_MX_* = 12.5 Hz, C_4_-H_M_ pyrazoline), 5.65 (1H, dd, *J_MX_* = 12.5 Hz, *J_AX_* = 6.0 Hz, C_5_-H_X_ pyrazoline), 6.99 (2H, d, *J* = 9.0 Hz, aromatic protons), 7.28 (2H, d, *J* = 8.5 Hz, aromatic protons), 7.35 (2H, d, *J* = 9.5 Hz, aromatic protons), 7.41 (2H, d, *J* = 8.5 Hz, aromatic protons), 7.94–7.98 (4H, m, 4-methylsulfonylphenyl protons). ^13^C-NMR (125 MHz, DMSO-*d*_6_) δ (ppm): 42.90 (CH_3_), 44.01 (CH_2_), 63.00 (CH), 115.70 (2CH), 126.84 (2CH), 127.86 (2CH), 128.33 (2CH), 129.59 (2CH), 132.16 (2CH), 132.69 (C), 137.16 (C), 140.68 (C), 141.00 (C), 142.97 (C), 147.13 (C), 160.45 (C). Anal. Calcd. For C_22_H_18_BrClN_2_O_2_S: C, 53.95; H, 3.70; N, 5.72. Found: C, 53.93; H, 3.69; N, 5.70. MS (ESI) (*m/z*): [M+1]^+^ 490.

*1,5-Bis(4-chlorophenyl)-3-(4-(methylsulfonyl)phenyl)-4,5-dihydro-1H-pyrazole* (**2j**): Yield: 73%; m.p. 209 °C. IR (KBr) ν_max_ (cm^−1^): 2918.10 (Aliphatic C-H), 1583.45, 1488.94 (C=N and C=C), 1307.65, 1151.42, 1087.78 (SO_2_ and C-N), 821.62 (C-H out of plane deformation). ^1^H-NMR (500 MHz, DMSO-*d*_6_) δ (ppm): 3.20 (1H, dd, *J_AM_* = 17.5 Hz, *J_AX_* = 6.0 Hz, C_4_-H_A_ pyrazoline), 3.23 (3H, s, SO_2_CH_3_), 3.96 (1H, dd, *J_MA_* = 18.0 Hz, *J_MX_* = 12.5 Hz, C_4_-H_M_ pyrazoline), 5.65 (1H, dd, *J_MX_* = 12.5 Hz, *J_AX_* = 6.0 Hz, C_5_-H_X_ pyrazoline), 7.03 (2H, d, *J* = 9.0 Hz, aromatic protons), 7.23 (2H, d, *J* = 9.0 Hz, aromatic protons), 7.29 (2H, d, *J* = 8.5 Hz, aromatic protons), 7.41 (2H, d, *J* = 8.5 Hz, aromatic protons), 7.94–7.98 (4H, m, 4-methylsulfonylphenyl protons). ^13^C-NMR (125 MHz, DMSO-*d*_6_) δ (ppm): 42.91 (CH_3_), 44.01 (CH_2_), 63.09 (CH), 115.23 (2CH), 126.82 (2CH), 127.86 (2CH), 128.34 (2CH), 129.32 (2CH), 129.90 (2CH), 132.69 (C), 137.18 (C), 140.66 (C), 141.05 (C), 142.64 (C), 147.04 (C), 160.48 (C). Anal. Calcd. for C_22_H_18_Cl_2_N_2_O_2_S: C, 59.33; H, 4.07; N, 6.29. Found: C, 59.32; H, 4.05; N, 6.30. MS (ESI) (*m/z*): [M+1]^+^ 446.

*1-(4-Methoxyphenyl)-5-(4-bromophenyl)-3-(4-(methylsulfonyl)phenyl)-4,5-dihydro-1H-pyrazole* (**2k**): Yield: 68%; m.p. 145 °C. IR (KBr) ν_max_ (cm^−1^): 2927.74 (Aliphatic C-H asymmetric), 2827.45 (Aliphatic C-H symmetric), 1510.16, 1417.58 (C=N and C=C), 1313.43, 1242.07, 1151.42, 1087.78 (SO_2_, C-N and C-O), 813.90 (C-H out of plane deformation). ^1^H-NMR (500 MHz, DMSO-*d*_6_) δ (ppm): 6.97 (d, *J* = 3.6 Hz, 1H), 7.11 (d, *J* = 3.2 Hz, 1H), 7.43–7.49 (m, 3H), 7.83–7.89 (m, 3H), 7.93–7.95 (m, 2H), 8.13–8.14 (m, 1H), 12.37 (brs, 1H). ^13^C-NMR (125 MHz, DMSO-*d*_6_) δ (ppm): 42.17 (CH_3_), 43.56 (CH_2_), 55.16 (CH_3_), 63.56 (CH), 114.45 (2CH), 114.73 (2CH), 120.56 (C), 125.87 (2CH), 127.33 (2CH), 128.33 (2CH), 131.88 (2CH), 137.12 (C), 137.57 (C), 139.54 (C), 141.52 (C), 144.59 (C), 153.19 (C). Anal. Calcd. for C_23_H_21_BrN_2_O_3_S: C, 56.91; H, 4.36; N, 5.77. Found: C, 56.90; H, 4.35; N, 5.76. MS (ESI) (*m/z*): [M+1]^+^ 486.

*1-(4-Fluorophenyl)-5-(4-bromophenyl)-3-(4-(methylsulfonyl)phenyl)-4,5-dihydro-1H-pyrazole* (**2l**): Yield: 75%; m.p. 196 °C. IR (KBr) ν_max_ (cm^−1^): 3031.89 (Aromatic C-H), 2918.10 (Aliphatic C-H), 1577.66, 1506.30, 1406.01 (C=N and C=C), 1307.65, 1218.93, 1151.42, 1010.63 (SO_2_ and C-N), 825.48 (C-H out of plane deformation). ^1^H-NMR (500 MHz, DMSO-*d*_6_) δ (ppm): 3.17 (1H, dd, *J_AM_* = 18.0 Hz, *J_AX_* = 7.0 Hz, C_4_-H_A_ pyrazoline), 3.23 (3H, s, SO_2_CH_3_), 3.95 (1H, dd, *J_MA_* = 16.5 Hz, *J_MX_* = 12.5 Hz, C_4_-H_M_ pyrazoline), 5.59 (1H, dd, *J_MX_* = 11.0 Hz, *J_AX_* = 6.0 Hz, C_5_-H_X_ pyrazoline), 7.04 (4H, s, aromatic protons), 7.24–7.26 (2H, m, aromatic protons), 7.53–7.55 (2H, m, aromatic protons), 7.95 (4H, s, 4-methylsulfonylphenyl protons). ^13^C-NMR (125 MHz, DMSO-*d*_6_) δ (ppm): 42.36 (CH_3_), 43.52 (CH_2_), 63.17 (CH), 114.57 (CH), 115.50 (CH), 115.68 (2CH), 120.66 (CH), 126.16 (2CH), 127.34 (2CH), 128.26 (2CH), 131.96 (2C), 136.85 (CH), 139.96 (C), 140.17 (C), 141.20 (C), 145.82 (C), 155.37 (C). Anal. Calcd. for C_22_H_18_BrFN_2_O_2_S: C, 55.82; H, 3.83; N, 5.92. Found: C, 55.82; H, 3.81; N, 5.93. MS (ESI) (*m/z*): [M+1]^+^ 474.

*1-(4-Methylphenyl)-5-(4-bromophenyl)-3-(4-(methylsulfonyl)phenyl)-4,5-dihydro-1H-pyrazole* (**2m**): Yield: 87%; m.p. 227 °C. IR (KBr) ν_max_ (cm^−1^): 3016.46 (Aromatic C-H), 2918.10 (Aliphatic C-H), 1585.38, 1508.23, 1407.94 (C=N and C=C), 1294.15, 1242.07, 1151.42, 1085.85, 1010.63 (SO_2_ and C-N), 821.62 (C-H out of plane deformation). ^1^H-NMR (500 MHz, DMSO-*d*_6_) δ (ppm): 2.17 (3H, s, CH_3_), 3.15 (1H, dd, *J_AM_* = 17.5 Hz, *J_AX_* = 6.0 Hz, C_4_-H_A_ pyrazoline), 3.22 (3H, s, SO_2_CH_3_), 3.92 (1H, dd, *J_MA_* = 17.5 Hz, *J_MX_* = 12.5 Hz, C_4_-H_M_ pyrazoline), 5.59 (1H, dd, *J_MX_* = 12.5 Hz, *J_AX_* = 6.0 Hz, C_5_-H_X_ pyrazoline), 6.94 (2H, d, *J* = 8.5 Hz), 6.99 (2H, d, *J* = 8.0 Hz), 7.22 (2H, d, *J* = 8.5 Hz, aromatic protons), 7.53 (2H, d, *J* = 8.5 Hz, aromatic protons), 7.93 (4H, s, 4-methylsulfonylphenyl protons). ^13^C-NMR (125 MHz, DMSO-*d*_6_) δ (ppm): 20.58 (CH_3_), 42.59 (CH_3_), 44.05 (CH_2_), 63.42 (CH), 113.92 (2CH), 121.02 (C), 126.50 (2CH), 127.83 (2CH), 128.73 (2CH), 128.77 (2CH), 129.94 (2CH), 132.38 (C), 137.54 (C), 140.20 (C), 141.62 (C), 142.00 (C), 145.47 (C). Anal. Calcd. for C_23_H_21_BrN_2_O_2_S: C, 58.85; H, 4.51; N, 5.97. Found: C, 58.85; H, 4.50; N, 5.95. MS (ESI) (*m/z*): [M+1]^+^ 470.

*1,5-Bis(4-bromophenyl)-3-(4-(methylsulfonyl)phenyl)-4,5-dihydro-1H-pyrazole* (**2n**): Yield: 94%; m.p. 225 °C. IR (KBr) ν_max_ (cm^−1^): 3012.60 (Aromatic C-H), 2918.10 (Aliphatic C-H), 1583.45, 1487.01, 1409.87 (C=N and C=C), 1307.65, 1151.42, 1085.85, 1010.63 (SO_2_ and C-N), 819.69 (C-H out of plane deformation). ^1^H-NMR (500 MHz, DMSO-*d*_6_) δ (ppm): 3.20 (1H, dd, *J_AM_* = 18.0 Hz, *J_AX_* = 6.0 Hz, C_4_-H_A_ pyrazoline), 3.23 (3H, s, SO_2_CH_3_), 3.96 (1H, dd, *J_MA_* = 17.5 Hz, *J_MX_* = 12.5 Hz, C_4_-H_M_ pyrazoline), 5.63 (1H, dd, *J_MX_* = 12.0 Hz, *J_AX_* = 5.5 Hz, C_5_-H_X_ pyrazoline), 6.98 (2H, d, *J* = 8.5 Hz), 7.21 (2H, d, *J* = 8.0 Hz, aromatic protons), 7.35 (2H, d, *J* = 9.0 Hz, aromatic protons), 7.54 (2H, d, *J* = 8.5 Hz, aromatic protons), 7.93–7.97 (4H, m, 4-methylsulfonylphenyl protons). ^13^C-NMR (125 MHz, DMSO-*d*_6_) δ (ppm): 42.34 (CH_3_), 43.51 (CH_2_), 62.54 (CH), 110.74 (2CH), 115.19 (C), 120.72 (C), 126.33 (2CH), 127.35 (2CH), 128.15 (2CH), 131.99 (2CH), 136.64 (2CH), 140.17 (C), 140.91 (C), 142.45 (C), 146.62 (C), 151.70 (C). Anal. Calcd. for C_22_H_18_Br_2_N_2_O_2_S: C, 49.46; H, 3.40; N, 5.24. Found: C, 49.44; H, 3.41; N, 5.25. MS (ESI) (*m/z*): [M+1]^+^ 535.

*1-(4-Chlorophenyl)-5-(4-bromophenyl)-3-(4-(methylsulfonyl)phenyl)-4,5-dihydro-1H-pyrazole* (**2o**): Yield: 95%; m.p. 222 °C. IR (KBr) ν_max_ (cm^−1^): 3045.39 (Aromatic C-H), 2918.10 (Aliphatic C-H), 1583.45, 1488.94 (C=N and C=C), 1307.65, 1151.42, 1085.85 (SO_2_ and C-N), 819.69 (C-H out of plane deformation). ^1^H-NMR (500 MHz, DMSO-*d*_6_) δ (ppm): 3.20 (1H, dd, *J_AM_* = 18.0 Hz, *J_AX_* = 6.0 Hz, C_4_-H_A_pyrazoline), 3.23 (3H, s, SO_2_CH_3_), 3.96 (1H, dd, *J_MA_* = 18.0 Hz, *J_MX_* = 12.5 Hz, C_4_-H_M_ pyrazoline), 5.63 (1H, dd, *J_MX_* = 12.5 Hz, *J_AX_* = 6.0 Hz, C_5_-H_X_ pyrazoline), 7.03 (2H, d, *J* = 9.0 Hz, aromatic protons), 7.22–7.24 (4H, m, aromatic protons), 7.54 (2H, d, *J* = 8.5 Hz, aromatic protons), 7.94–7.98 (4H, m, 4-methylsulfonylphenyl protons). ^13^C-NMR (125 MHz, DMSO-*d*_6_) δ (ppm): 42.35 (CH_3_), 43.51 (CH_2_), 62.64 (CH), 114.71 (2CH), 120.71 (C), 123.06 (CH), 126.31 (CH), 127.35 (2CH), 128.17 (2CH), 128.82 (2CH), 131.99 (2CH), 136.66 (C), 140.15 (2C), 140.96 (C), 142.12 (C), 146.53 (C). Anal. Calcd. for C_22_H_18_BrClN_2_O_2_S: C, 53.95; H, 3.70; N, 5.72. Found: C, 53.93; H, 3.69; N, 5.74. MS (ESI) (*m/z*): [M+1]^+^ 490.

*1-Phenyl-5-(4-bromophenyl)-3-(4-(methylsulfonyl)phenyl)-4,5-dihydro-1H-pyrazole* (**2p**): Yield: 86%; m.p. 205 °C. IR (KBr) ν_max_ (cm^−1^): 3020.32 (Aromatic C-H), 2921.96 (Aliphatic C-H), 1595.02, 1485.09, 1407.94 (C=N and C=C), 1303.79, 1151.42, 1010.63 (SO_2_ and C-N), 821.62, 750.26 (C-H out of plane deformation). ^1^H-NMR (500 MHz, DMSO-*d*_6_) δ (ppm): 3.17 (1H, dd, *J_AM_* = 17.5 Hz, *J_AX_* = 6.0 Hz, C_4_-H_A_ pyrazoline), 3.23 (3H, s, SO_2_CH_3_), 3.94 (1H, dd, *J_MA_* = 17.5 Hz, *J_MX_* = 12.5 Hz, C_4_-H_M_ pyrazoline), 5.62 (1H, dd, *J_MX_* = 12.0 Hz, *J_AX_* = 6.0 Hz, C_5_-H_X_ pyrazoline), 6.77 (1H, m, aromatic protons), 7.04 (2H, d, *J* = 8.0 Hz, aromatic protons), 7.17–7.20 (2H, m, aromatic protons), 7.24 (2H, d, *J* = 8.5 Hz, aromatic protons), 7.53 (2H, d, *J* = 8.5 Hz, aromatic protons), 7.93–7.97 (4H, m, 4-methylsulfonylphenyl protons). ^13^C-NMR (125 MHz, DMSO-*d*_6_) δ (ppm): 42.19 (CH_3_), 43.54 (CH_2_), 62.71 (CH), 113.28 (2CH), 119.47 (C), 120.57 (CH), 126.14 (2CH), 127.34 (2CH), 128.18 (2CH), 129.00 (2CH), 131.93 (2CH), 136.92 (C), 139.90 (C), 141.45 (C), 143.28 (C), 145.60 (C). Anal. Calcd. for C_22_H_19_BrN_2_O_2_S: C, 58.03; H, 4.21; N, 6.15. Found: C, 58.01; H, 4.20; N, 6.17. MS (ESI) (*m/z*): [M+1]^+^ 456.

*1-Phenyl-5-(4-chlorophenyl)-3-(4-(methylsulfonyl)phenyl)-4,5-dihydro-1H-pyrazole* (**2r**): Yield: 81%; m.p. 212 °C. IR (KBr) ν_max_ (cm^−1^): 3020.32 (Aromatic C-H), 2921.96 (Aliphatic C-H), 1596.95, 1492.80, 1411.80 (C=N and C=C), 1303.79, 1151.42, 1087.78 (SO_2_ and C-N), 823.55, 750.26 (C-H out of plane deformation). ^1^H-NMR (500 MHz, DMSO-*d*_6_) δ (ppm): 3.17 (1H, dd, *J_AM_* = 17.5 Hz, *J_AX_* = 6.0 Hz, C_4_-H_A_ pyrazoline), 3.23 (3H, s, SO_2_CH_3_), 3.94 (1H, dd, *J_MA_* = 18.0 Hz, *J_MX_* = 13.0 Hz, C_4_-H_M_ pyrazoline), 5.63 (1H, dd, *J_MX_* = 12.0 Hz, *J_AX_* = 6.0 Hz, C_5_-H_X_ pyrazoline), 6.76–6.79 (1H, m, aromatic protons), 7.04 (2H, d, *J* = 8.0 Hz, aromatic protons), 7.17–7.20 (2H, m, aromatic protons), 7.30 (2H, d, *J* = 8.5 Hz, aromatic protons), 7.40 (2H, d, *J* = 8.5 Hz, aromatic protons), 7.95–7.97 (4H, m, 4-methylsulfonylphenyl protons). ^13^C-NMR (125 MHz, DMSO-*d*_6_) δ (ppm): 42.24 (CH_3_), 43.53 (CH_2_), 62.67 (CH), 113.29 (2CH), 119.46 (CH), 126.13 (2CH), 127.33 (2CH), 127.82 (2CH), 129.00 (3CH), 132.05 (CH), 136.93 (C), 139.89 (C), 141.01 (2C), 143.29 (C), 145.57 (C). Anal. Calcd. for C_22_H_19_ClN_2_O_2_S: C, 64.30; H, 4.66; N, 6.82. Found: C, 64.31; H, 4.65; N, 6.80. MS (ESI) (*m/z*): [M+1]^+^ 411.

*1-Phenyl-5-(4-fluorophenyl)-3-(4-(methylsulfonyl)phenyl)-4,5-dihydro-1H-pyrazole* (**2s**): Yield: 78%; m.p. 219 °C. IR (KBr) ν_max_ (cm^−1^): 3020.32 (Aromatic C-H), 2921.96 (Aliphatic C-H), 1595.02, 1492.80 (C=N and C=C), 1380.94, 1299.93, 1224.71, 1149.50, 1087.78 (SO_2_ and C-N), 837.05, 748.33 (C-H out of plane deformation). ^1^H-NMR (500 MHz, DMSO-*d*_6_) δ (ppm): 3.16 (1H, dd, *J_AM_* = 17.5 Hz, *J_AX_* = 6.0 Hz, C_4_-H_A_ pyrazoline), 3.23 (3H, s, SO_2_CH_3_), 3.93 (1H, dd, *J_MA_* = 17.5 Hz, *J_MX_* = 12.5 Hz, C_4_-H_M_ pyrazoline), 5.62 (1H, dd, *J_MX_* = 12.5 Hz, *J_AX_* = 6.5 Hz, C_5_-H_X_ pyrazoline), 6.75–6.78 (1H, m, aromatic protons), 7.06 (2H, d, *J* = 8.0 Hz, aromatic protons), 7.15–7.20 (4H, m, aromatic protons), 7.31–7.34 (2H, m, aromatic protons), 7.94–7.96 (4H, m, 4-methylsulfonylphenyl protons). ^13^C-NMR (125 MHz, DMSO-*d*_6_) δ (ppm): 42.37 (CH_3_), 43.55 (CH_2_), 62.68 (CH), 113.32 (2CH), 115.72 (CH), 115.89 (CH), 119.42 (CH), 126.11 (2CH), 127.34 (2CH), 127.96 (CH), 128.96 (2CH), 137.00 (CH), 138.22 (C), 139.85 (C), 143.36 (C), 145.51 (C), 160.41 (C), 162.35 (C). Anal. Calcd. for C_22_H_19_FN_2_O_2_S: C, 66.99; H, 4.85; N, 7.10. Found: C, 66.98; H, 4.82; N, 7.12. MS (ESI) (*m/z*): [M+1]^+^ 395.

### 3.3. Pharmacology

#### 3.3.1. Animals

Adult Balb/c male mice (30–35 g), obtained from Anadolu University Research Center for Animal Experiments, were used for the experiments. The animals were housed at room temperature of 24 ± 1 °C with 12/12 h light/dark cycle (lights on at 08:00 h). Temperature, sound, and light conditions were not altered during the course of the experiments. 12 h before each experiment, animals received only water, in order to avoid food interference with substances absorption. The experimental protocols were approved by the Local Ethical Committee on Animal Experimentation of Anadolu University, Eskişehir, Turkey.

#### 3.3.2. Assessment of Antidepressant Activity

##### Tail Suspension Test

TST was carried out by a method described earlier by Steru and co-workers [21]. The method was performed using an automatic TST apparatus (BioSeb, Vitrolles, France), as described previously [19]. The mice were taped by their tails on a metal hook in 3 test chambers (15 cm width × 19 cm height) constructed of white plastic walls and black plastic floors. Each hook was connected to a computerized strain gauge that was adjusted to detect all movements of the animals (Tail suspension software, Bioseb). Immobility time of mice was measured during the last 4 min of 6 min test duration [23].

##### Modified Forced Swimming Test

MFST was performed as described previously [15,24]. The mice were forced to swim individually in a glass cylinder (12 cm diameter × 30 cm height) containing 20 cm of water at 25 ± 1 °C. A 15-min pre-test was conducted 24 h before the 5-min swim test. During the test, time for swimming (horizontal movement on the surface of the water), climbing (upward-directed movements of the forepaws along the side of the cylinder), and immobility (movement required just to keep the head above the water) were recorded using a stopwatch.

#### 3.3.3. Assessment of Locomotor Activity

##### Activity Cage Test

The horizontal and vertical locomotor activity of the mice were monitored using an activity cage apparatus (Ugo Basile, No. 7420, Varese, Italy), which contains two pairs of 16 photocells 3 cm and 12 cm above the floor. Interruptions of light beams to the photocells during horizontal and vertical movements of the animals were automatically recorded for 4 min [15].

#### 3.3.4. Statistical Analyses

Statistical analyses were performed on data for seven animals (n = 7) from each group by using GraphPad Prism 3.0 software (GraphPad Software, San Diego, CA, USA). Comparisons between the experimental groups were performed by one-way ANOVA followed by Tukey’s test. The results were expressed as mean ± standard error of mean (SEM). Differences between the datasets were considered significant at *p* < 0.05.

## 4. Conclusions

In conclusion, this study supports the previous papers reporting the antidepressant-like activities of pyrazoline derivatives [7,8,9,10,11,12,13,14,15,16,17]. The antidepressant-like effects of compounds **2a**, **2h**, **2j**, **2k**, **2l**, **2m**, and **2n** seem to be related with the serotonergic system rather than the noradrenergic system. However, involvement of the serotonergic system in the observed antidepressant activity needs to be confirmed with further detailed studies.

## Supplementary Materials

Supplementary materials can be accessed at: http://www.mdpi.com/1420-3049/20/02/2668/s1.
