# Supplementary material for: Synthesis and Evaluation of New 1,5-Diaryl-3-[4-(methyl-sulfonyl)phenyl]-4,5-dihydro-1H-pyrazole Derivatives as Potential Antidepressant Agents
Source: Molecules. 2015 Feb 4;20(2):2668–84. doi: 10.3390/molecules20022668 (PMC6272793; doi:10.3390/molecules20022668)
Supplement: Supplementary file 1 [file molecules-20-02668-s001.pdf]

Supplementary Materials

Table S1. Some properties of compounds **2a**–**s**.

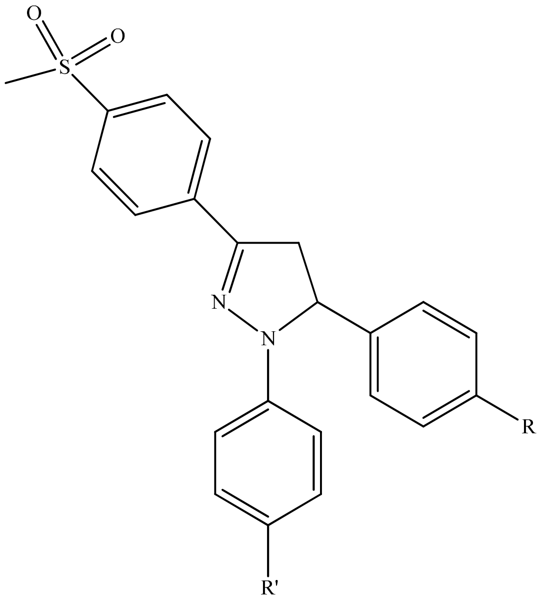

Compound	R	R'	Yield (%)	M.p. (°C)	Molecular Formula	Molecular Weight
**2a**	F	Cl	93	193	$C_{22}H_{18}ClFN_2O_2S$	428.91
**2b**	F	F	85	221	$C_{22}H_{18}F_2N_2O_2S$	412.45
**2c**	F	Br	77	169	$C_{22}H_{18}BrFN_2O_2S$	473.36
**2d**	F	OMe	60	170	$C_{23}H_{21}FN_2O_3S$	424.49
**2e**	F	Me	76	186	$C_{23}H_{21}FN_2O_2S$	408.49
**2f**	Cl	OMe	76	152	$C_{23}H_{21}ClN_2O_3S$	440.94
**2g**	Cl	F	87	203	$C_{22}H_{18}ClFN_2O_2S$	428.91
**2h**	Cl	Me	83	230	$C_{23}H_{21}ClN_2O_2S$	424.94
**2i**	Cl	Br	83	204	$C_{22}H_{18}BrClN_2O_2S$	489.81
**2j**	Cl	Cl	73	209	$C_{22}H_{18}Cl_2N_2O_2S$	445.36
**2k**	Br	OMe	68	145	$C_{23}H_{21}BrN_2O_3S$	485.39
**2l**	Br	F	75	196	$C_{22}H_{18}BrFN_2O_2S$	473.36
**2m**	Br	Me	87	227	$C_{23}H_{21}BrN_2O_2S$	469.39
**2n**	Br	Br	94	225	$C_{22}H_{18}Br_2N_2O_2S$	534.26
**2o**	Br	Cl	95	222	$C_{22}H_{18}BrClN_2O_2S$	489.81
**2p**	Br	H	86	205	$C_{22}H_{19}BrN_2O_2S$	455.37
**2r**	Cl	H	81	212	$C_{22}H_{19}ClN_2O_2S$	410.92
**2s**	F	H	78	219	$C_{22}H_{19}FN_2O_2S$	394.46

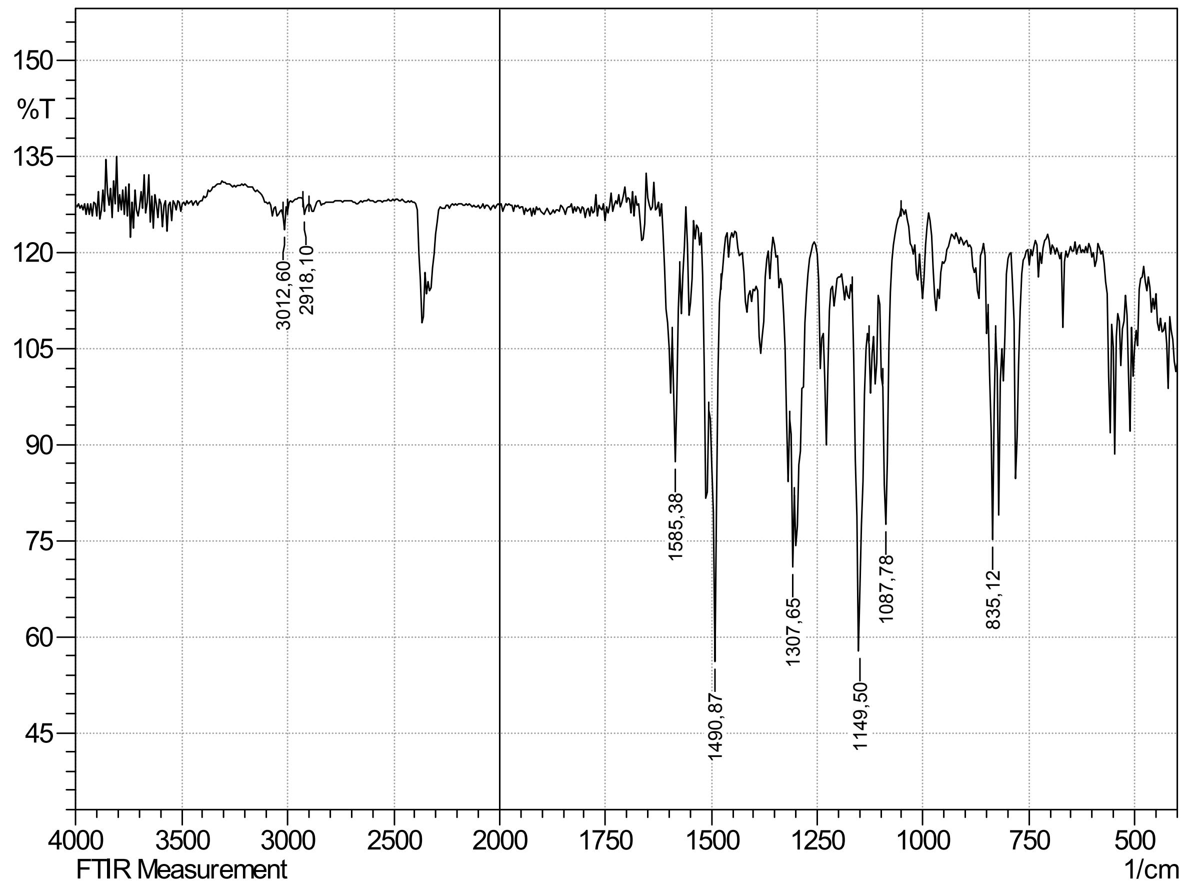

Figure S1. IR Spectrum of compound **2a**.

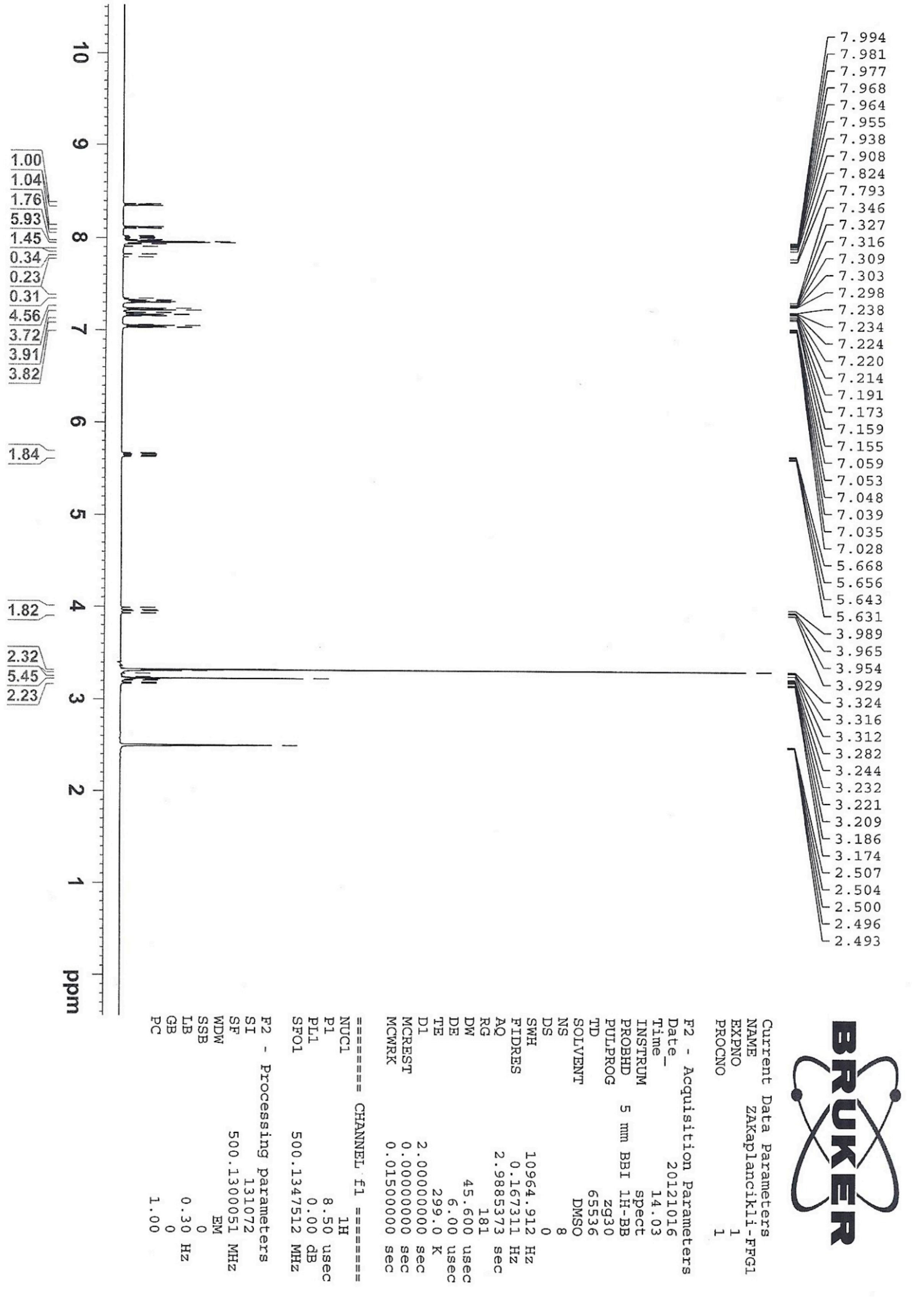

Figure S2. ^{1}H-NMR Spectrum of compound **2a**.

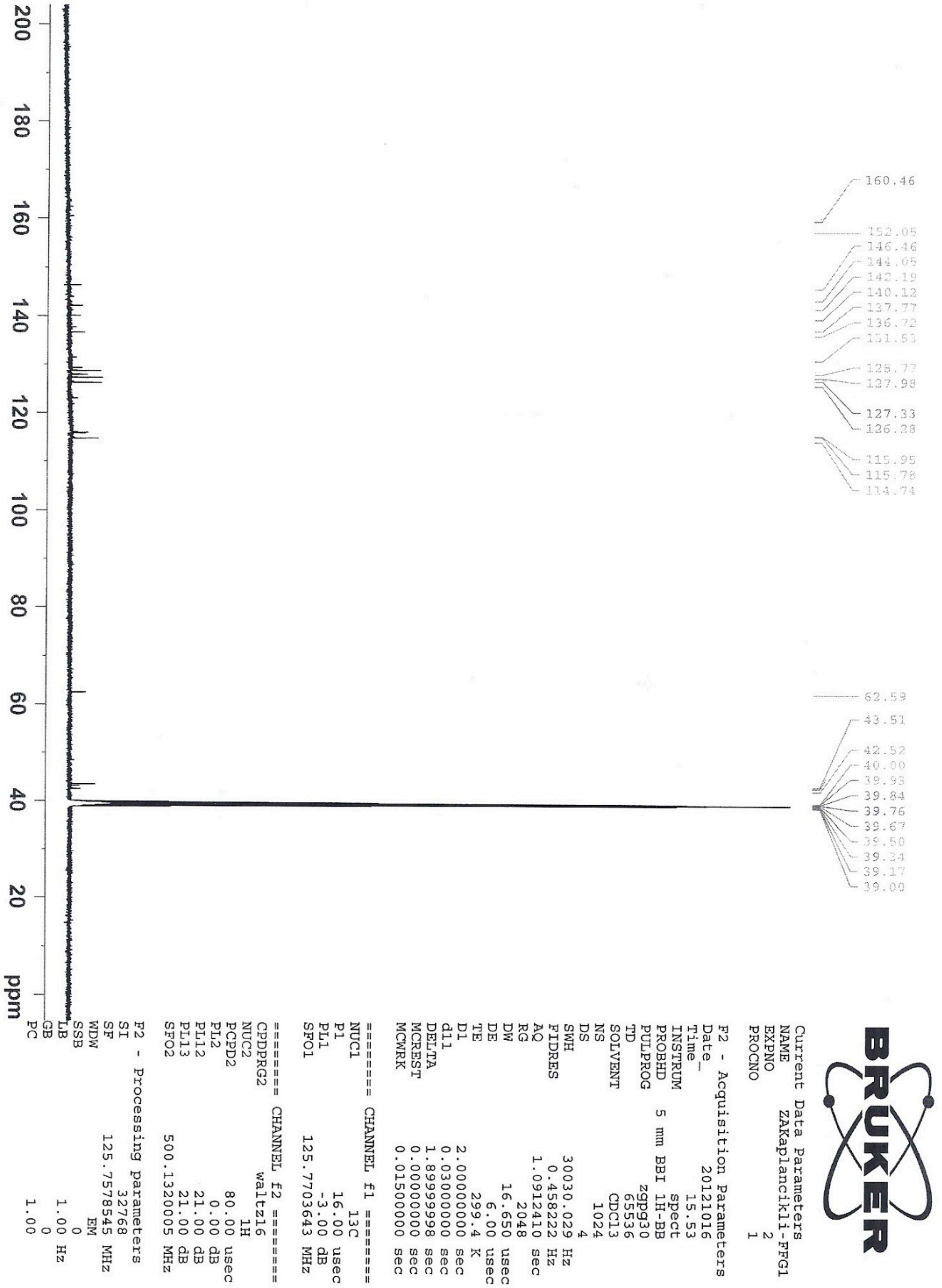

Figure S3. ^{13}C-NMR Spectrum of compound **2a**.

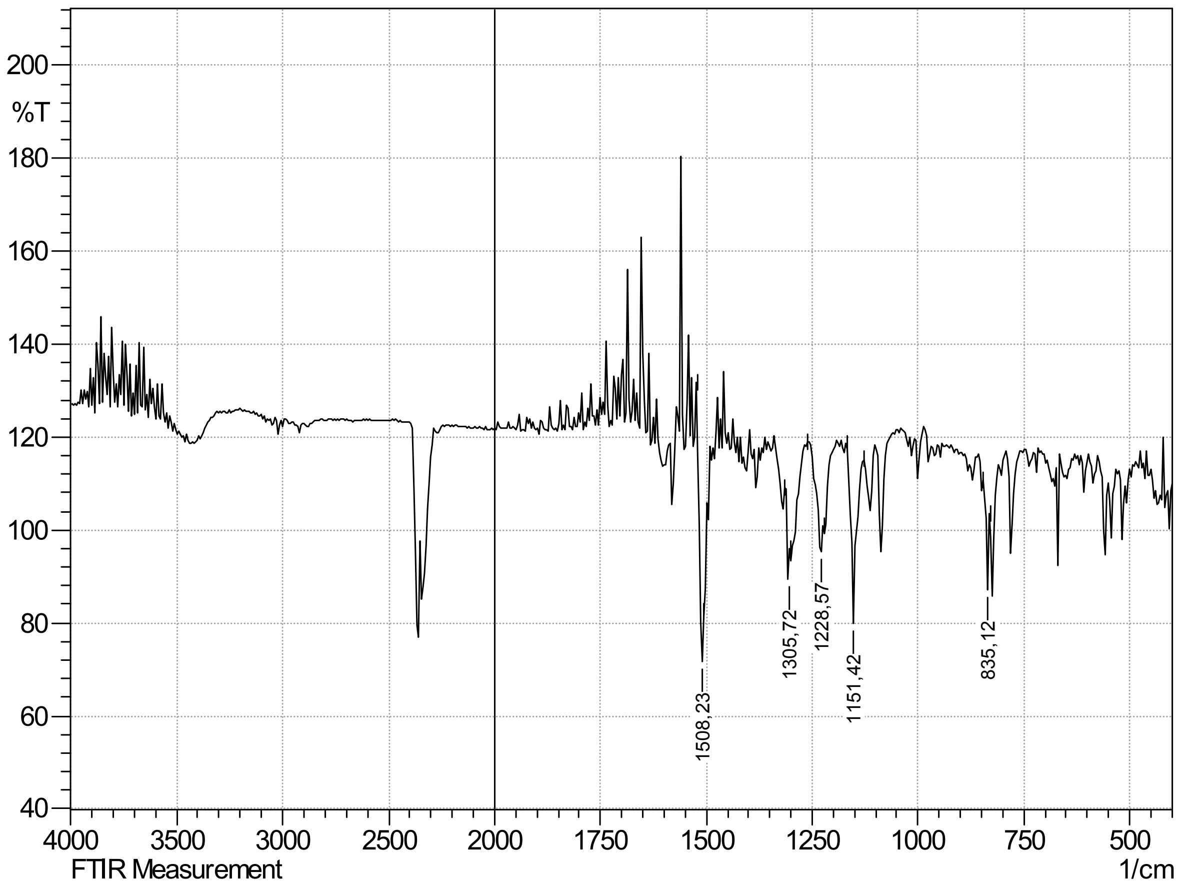

Figure S4. IR Spectrum of compound **2b**.

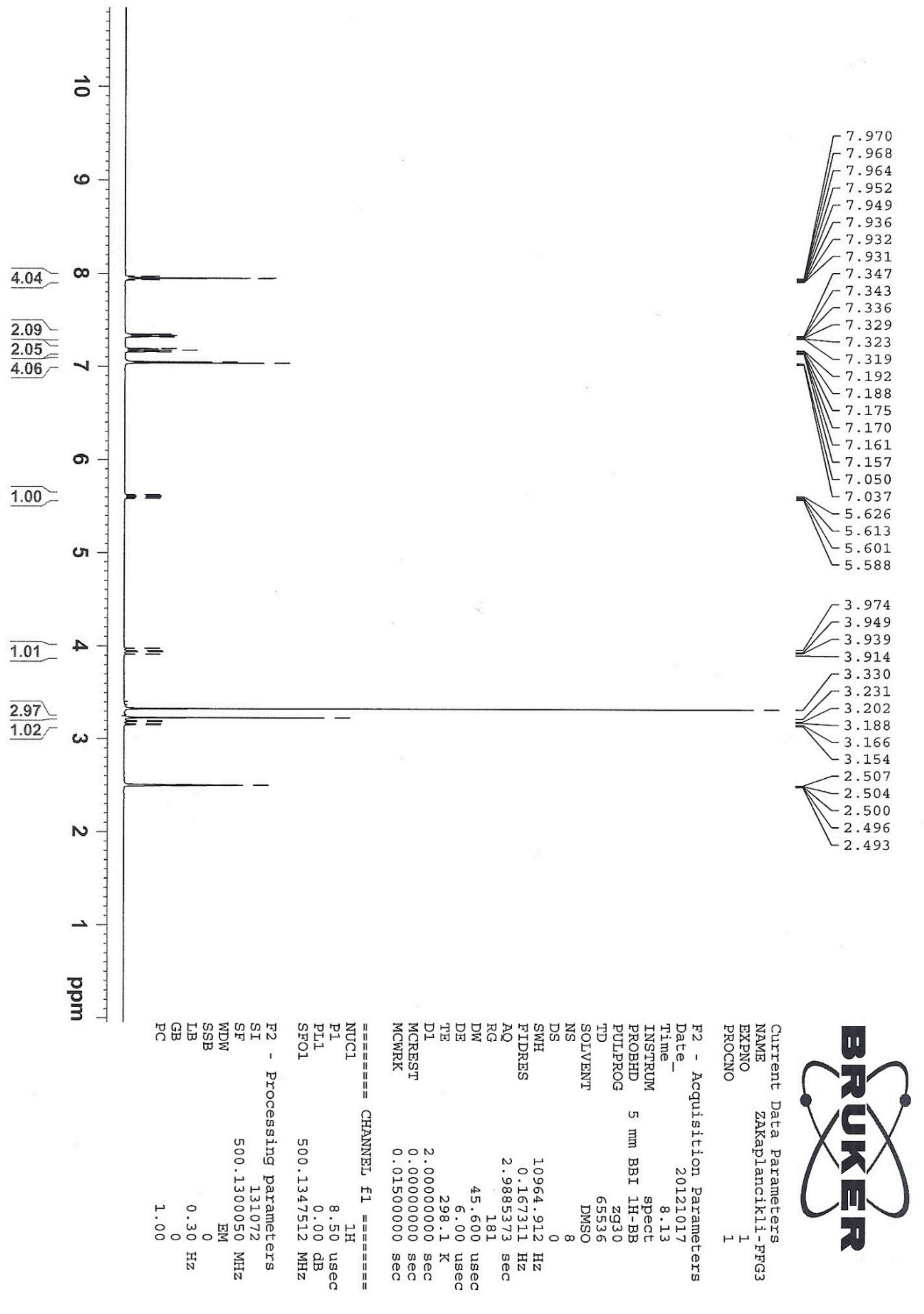

Figure S5. ^{1}H-NMR Spectrum of compound **2b**.

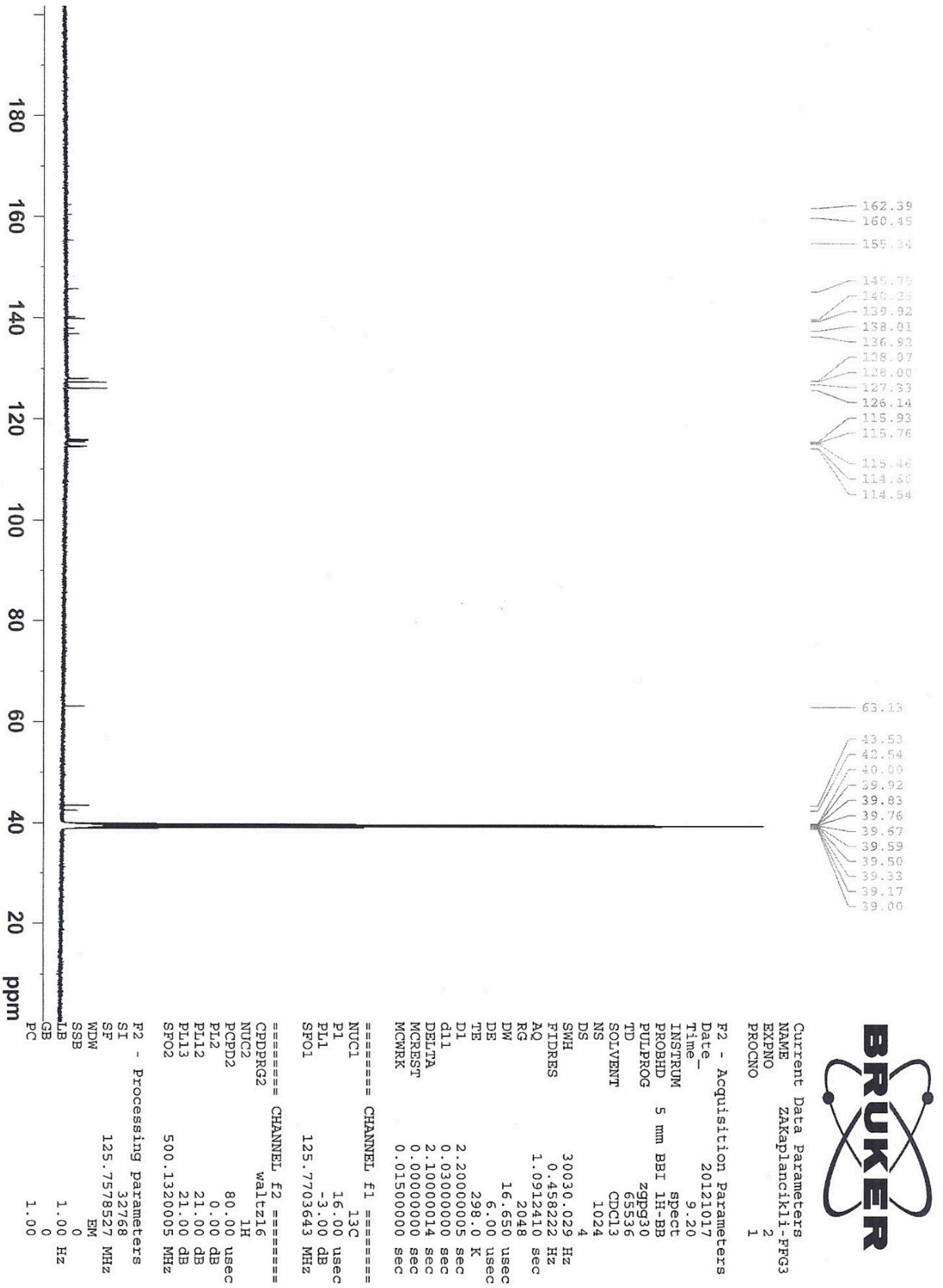

Figure S6. ^{13}C-NMR Spectrum of compound **2b**.

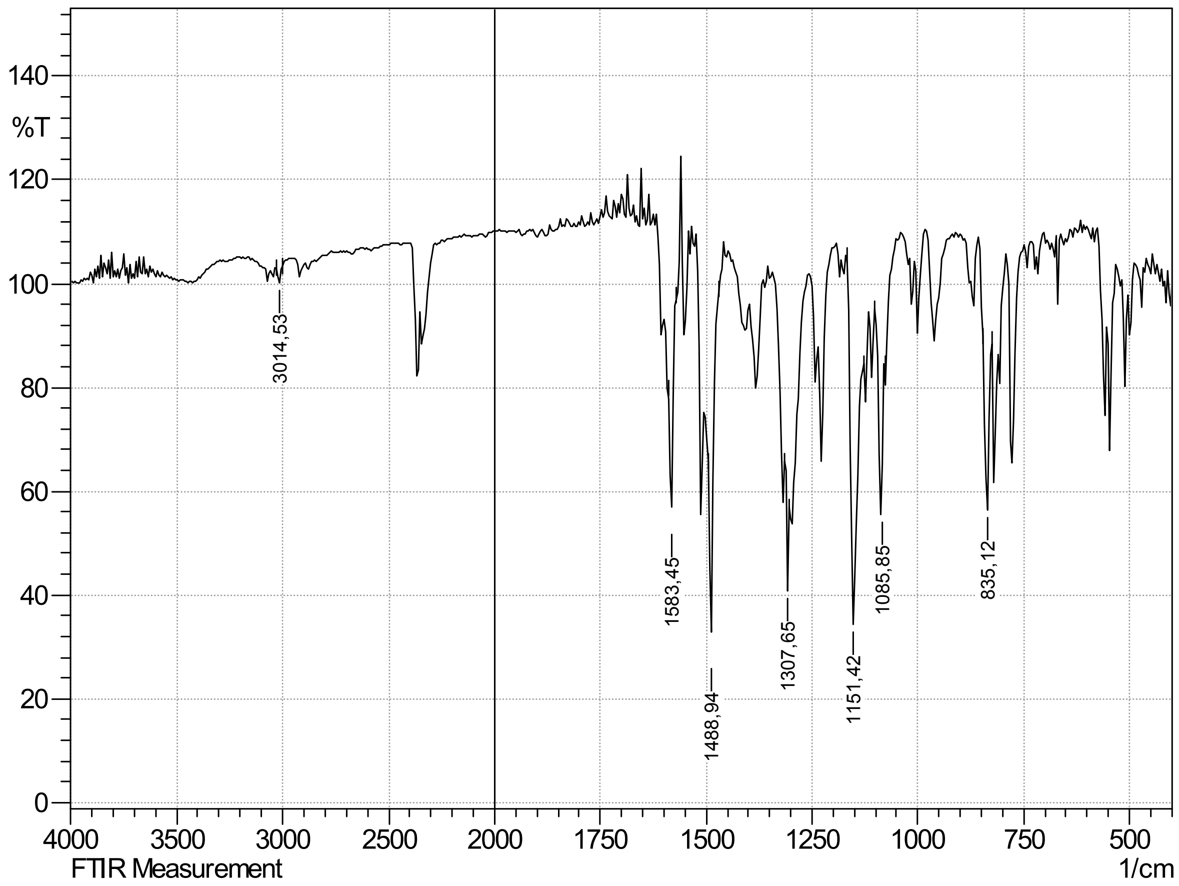

Figure S7. IR Spectrum of compound **2c**.

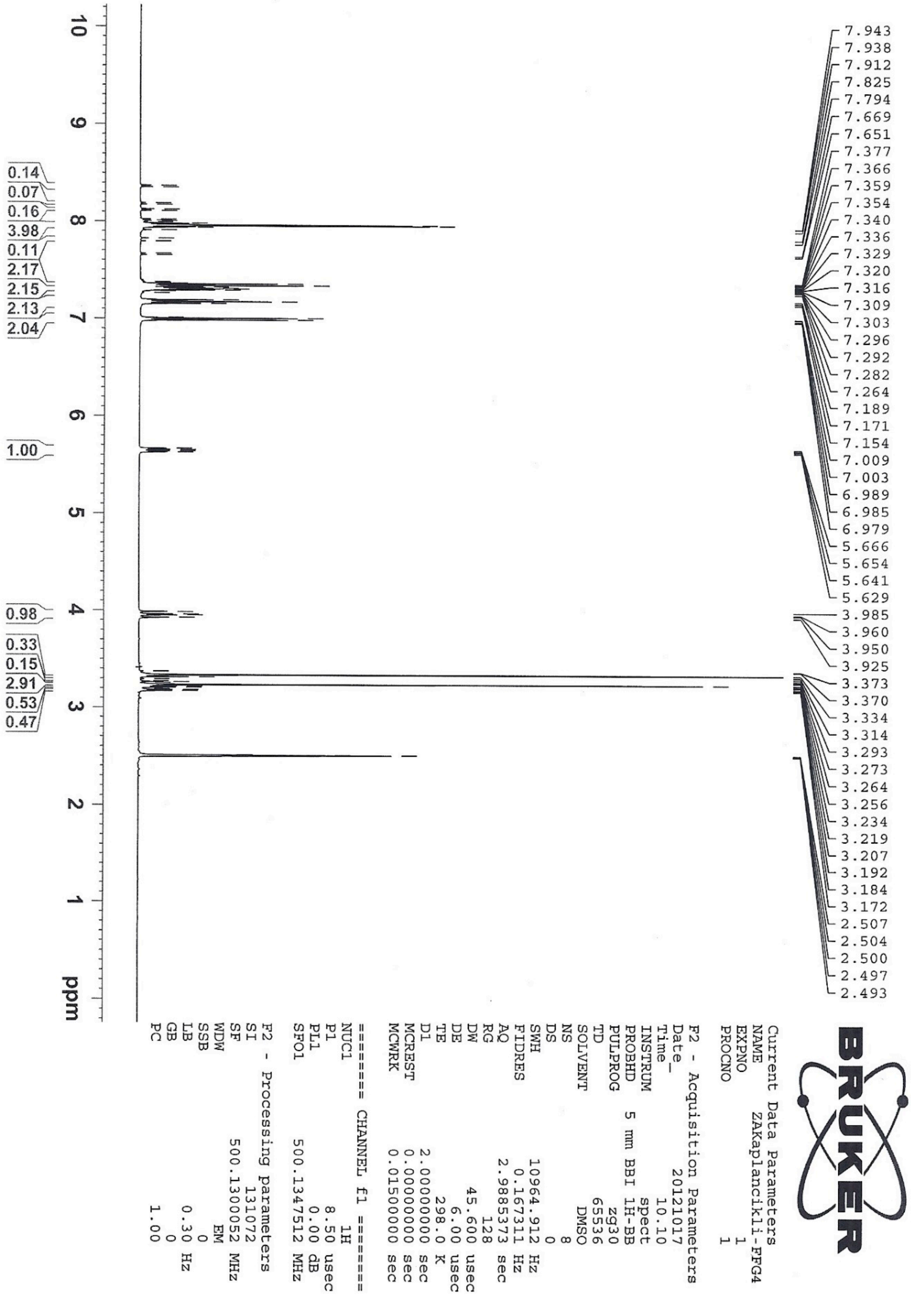

Figure S8. ^{1}H-NMR Spectrum of compound **2c**.

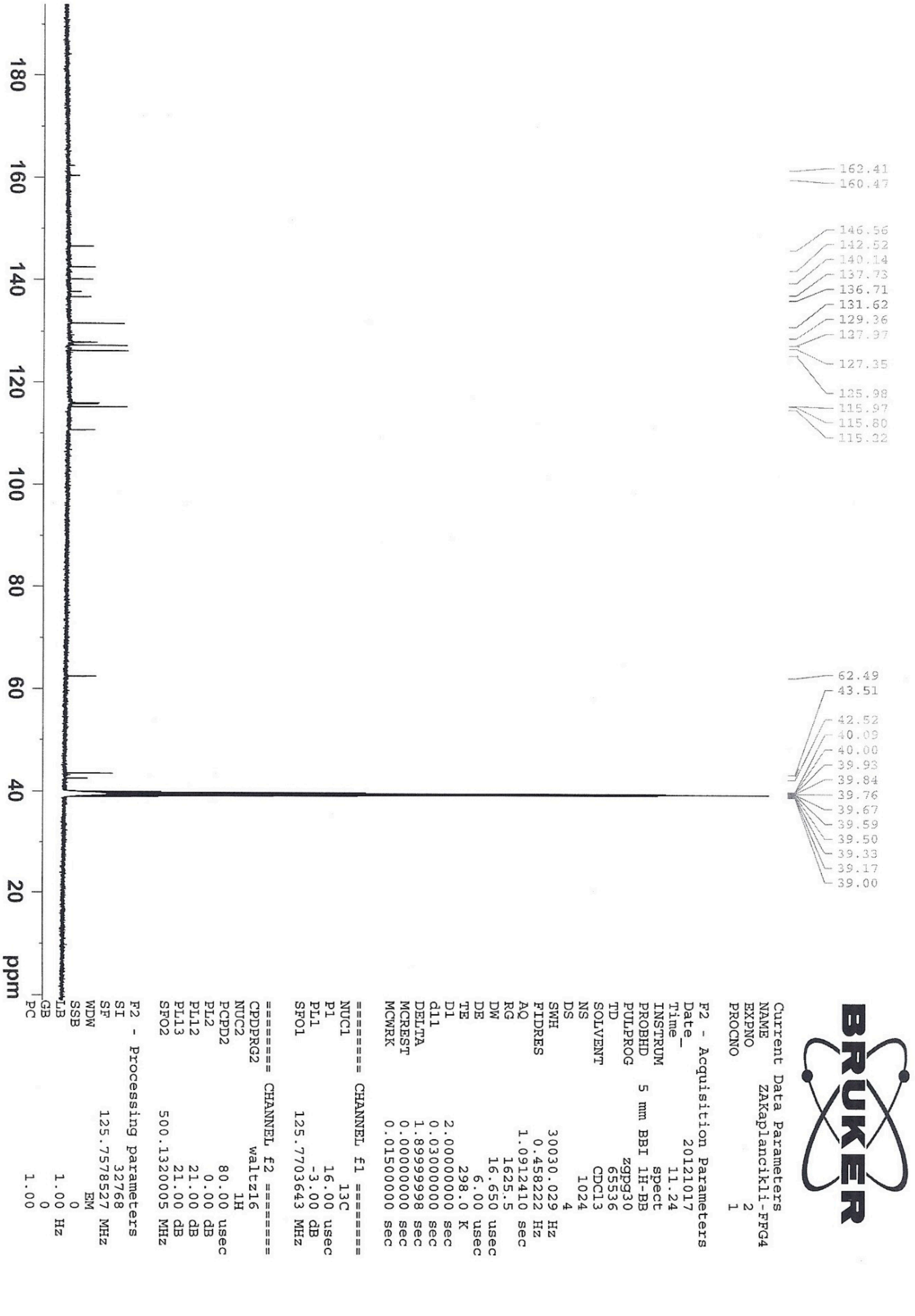

Figure S9. ^{13}C-NMR Spectrum of compound **2c**.

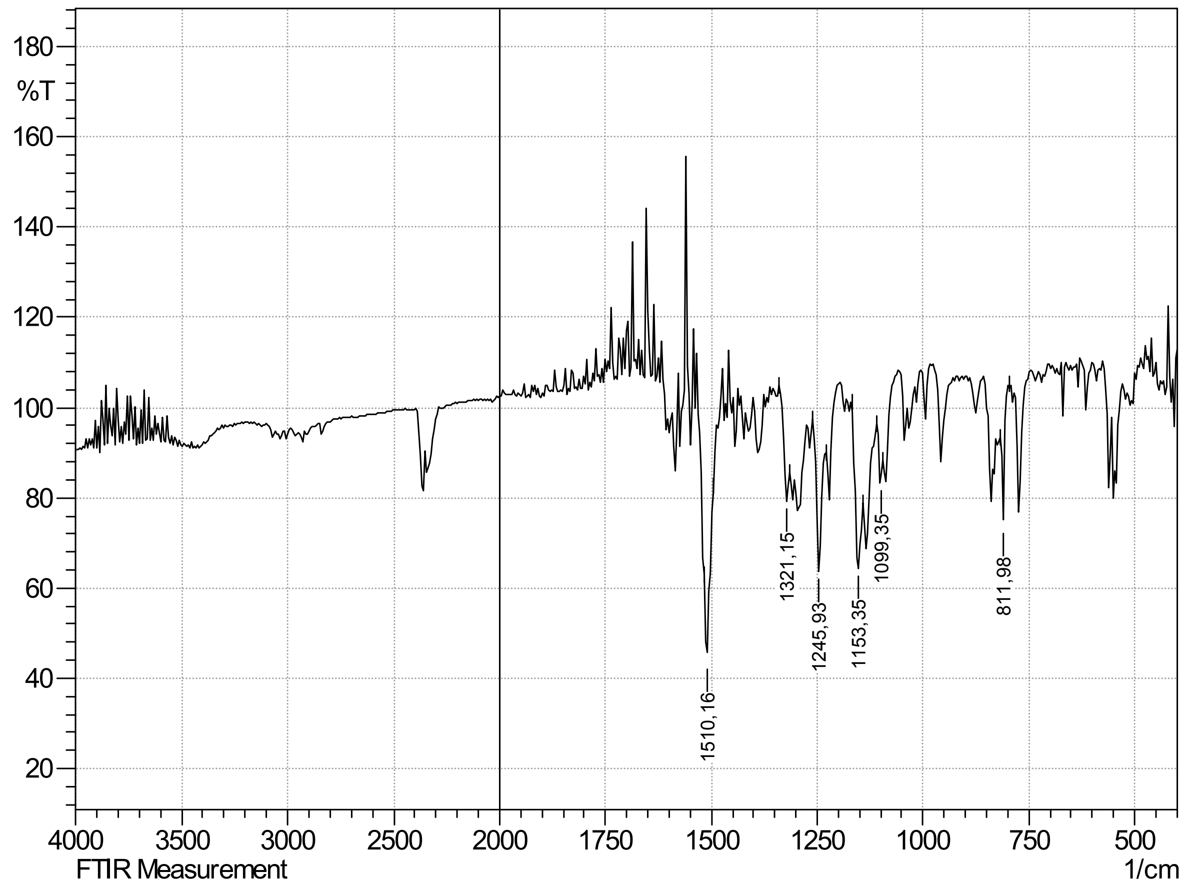

Figure S10. IR Spectrum of compound **2d**.

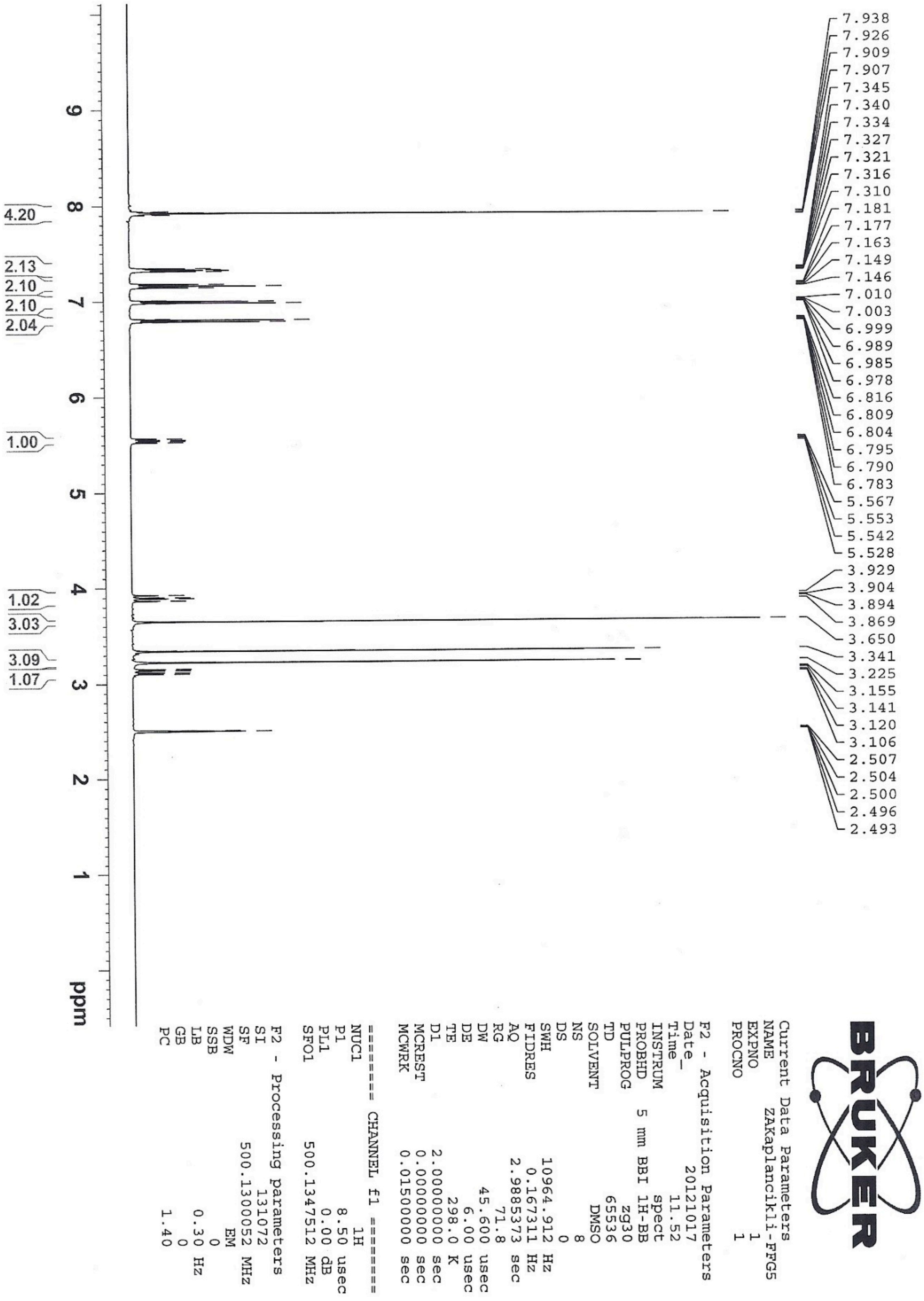

Figure S11. ^{1}H-NMR Spectrum of compound **2d**.

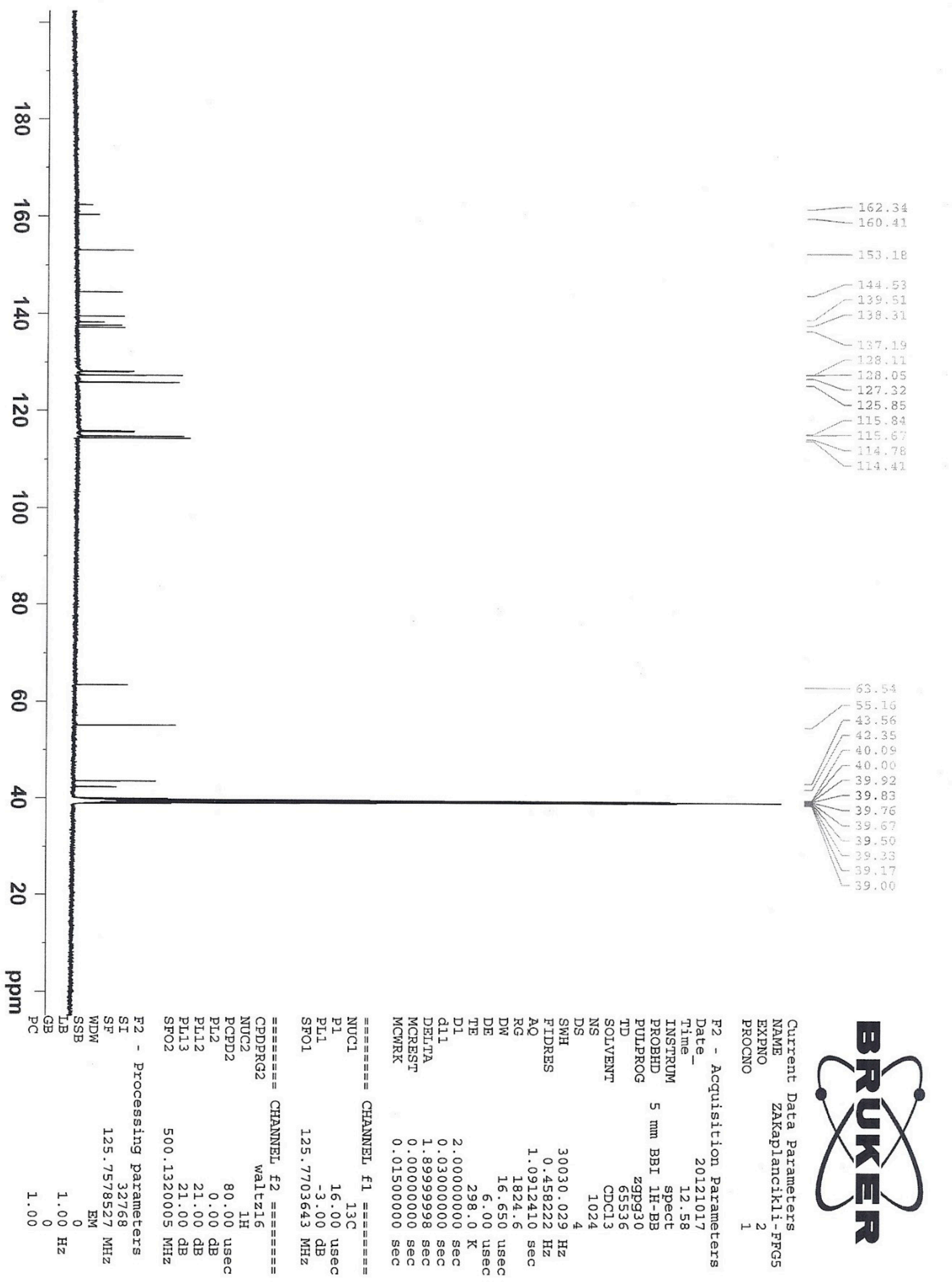

Figure S12. ^{13}C-NMR Spectrum of compound **2d**.

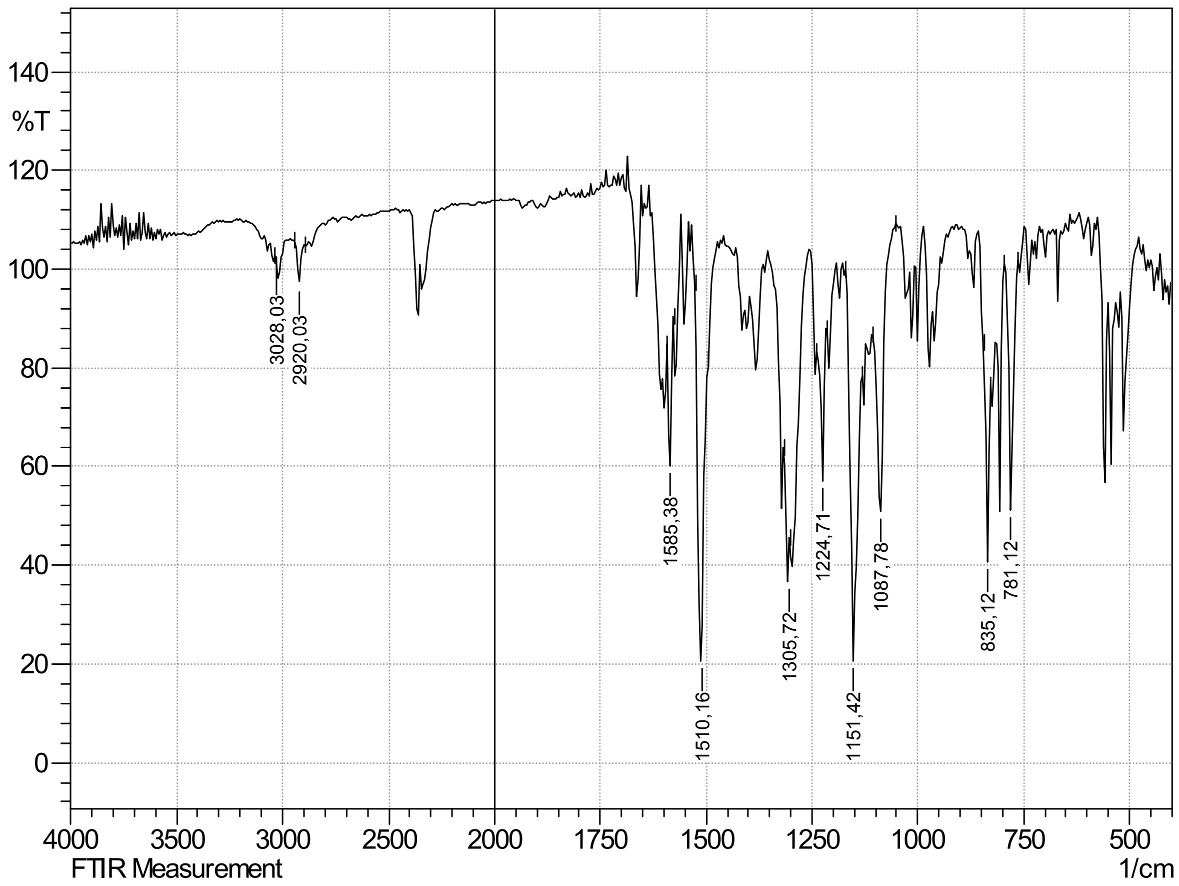

Figure S13. IR Spectrum of compound **2e**.

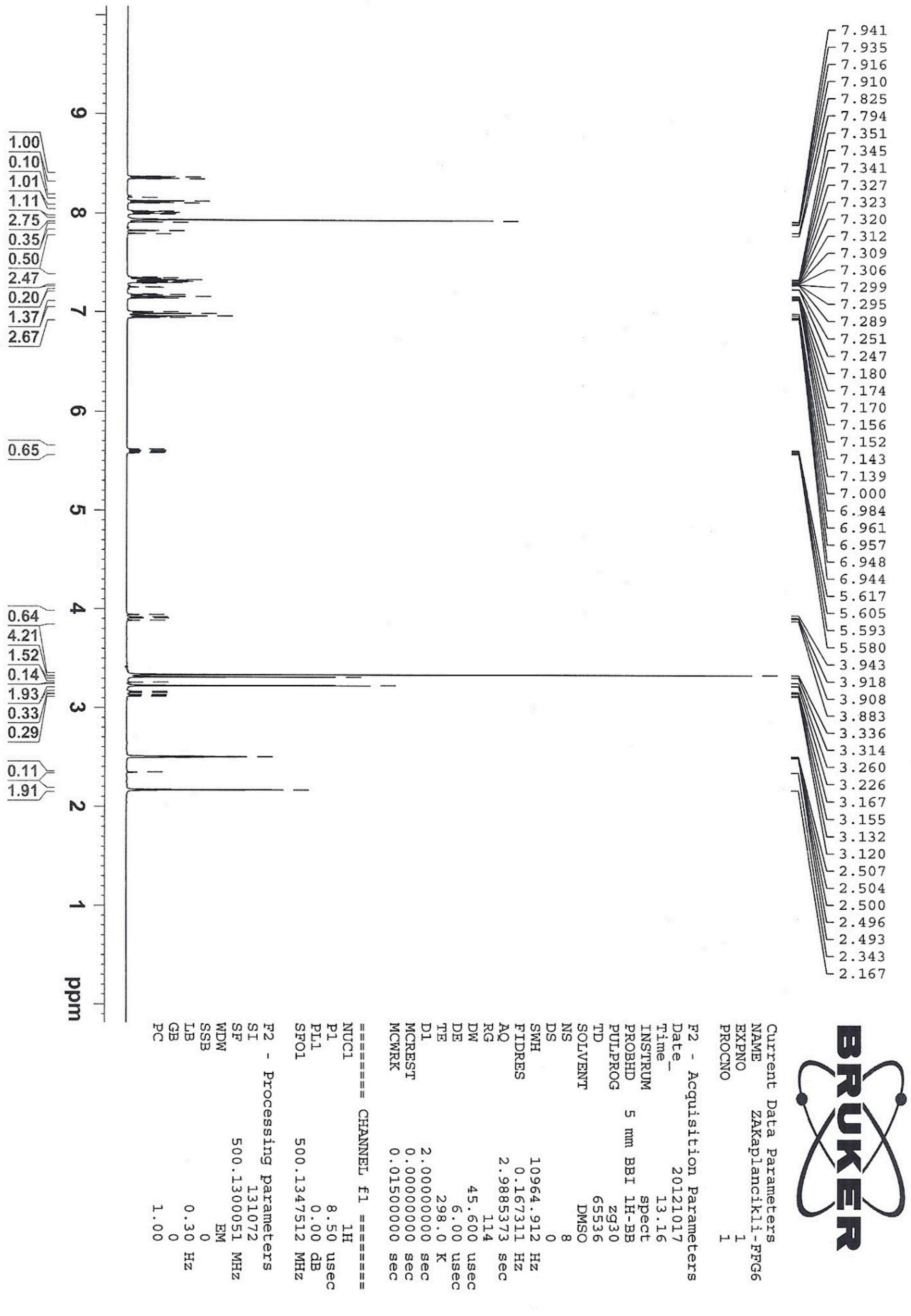

Figure S14. ^{1}H-NMR Spectrum of compound **2e**.

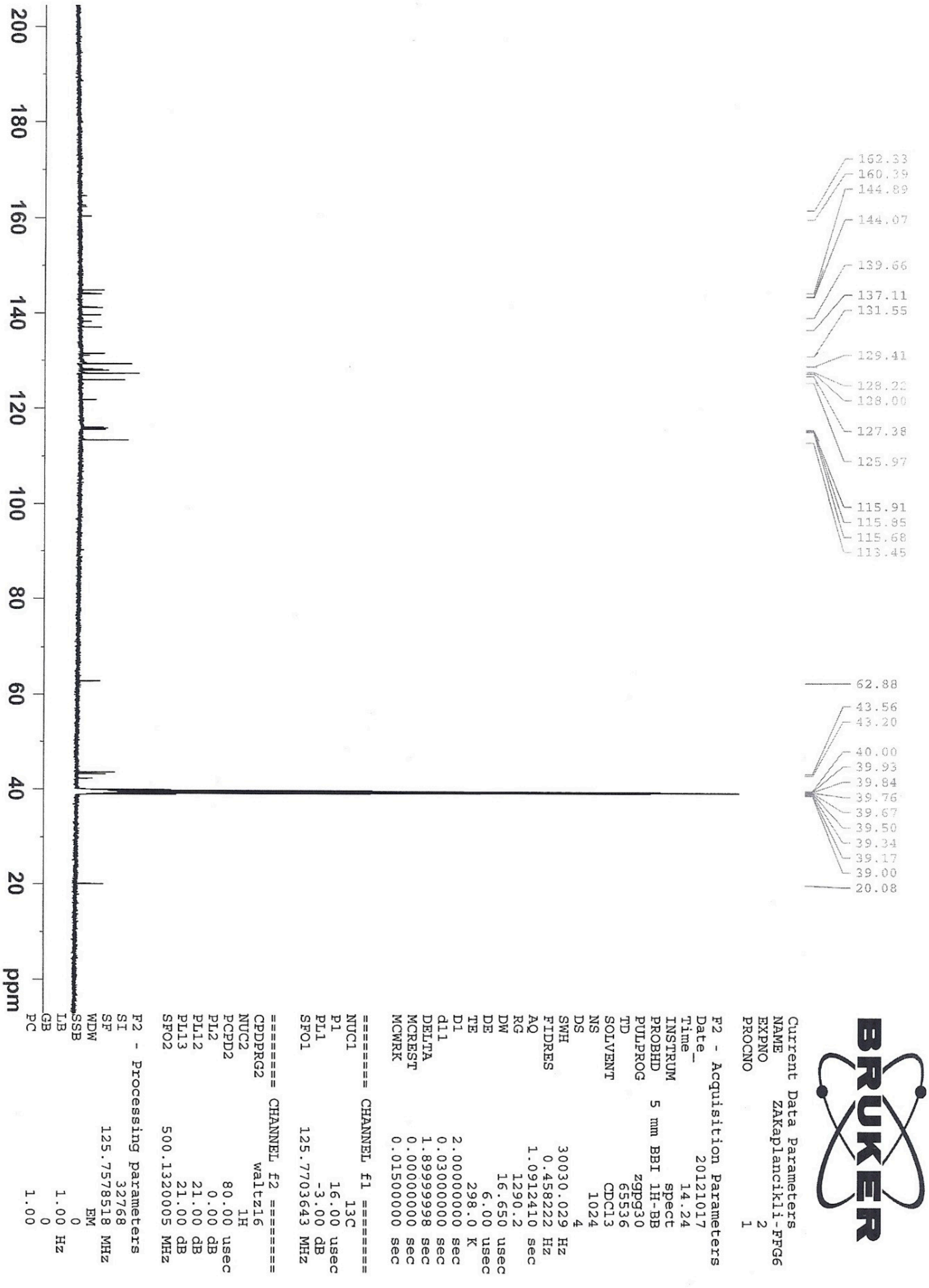

Figure S15. ^{13}C-NMR Spectrum of compound **2e**.

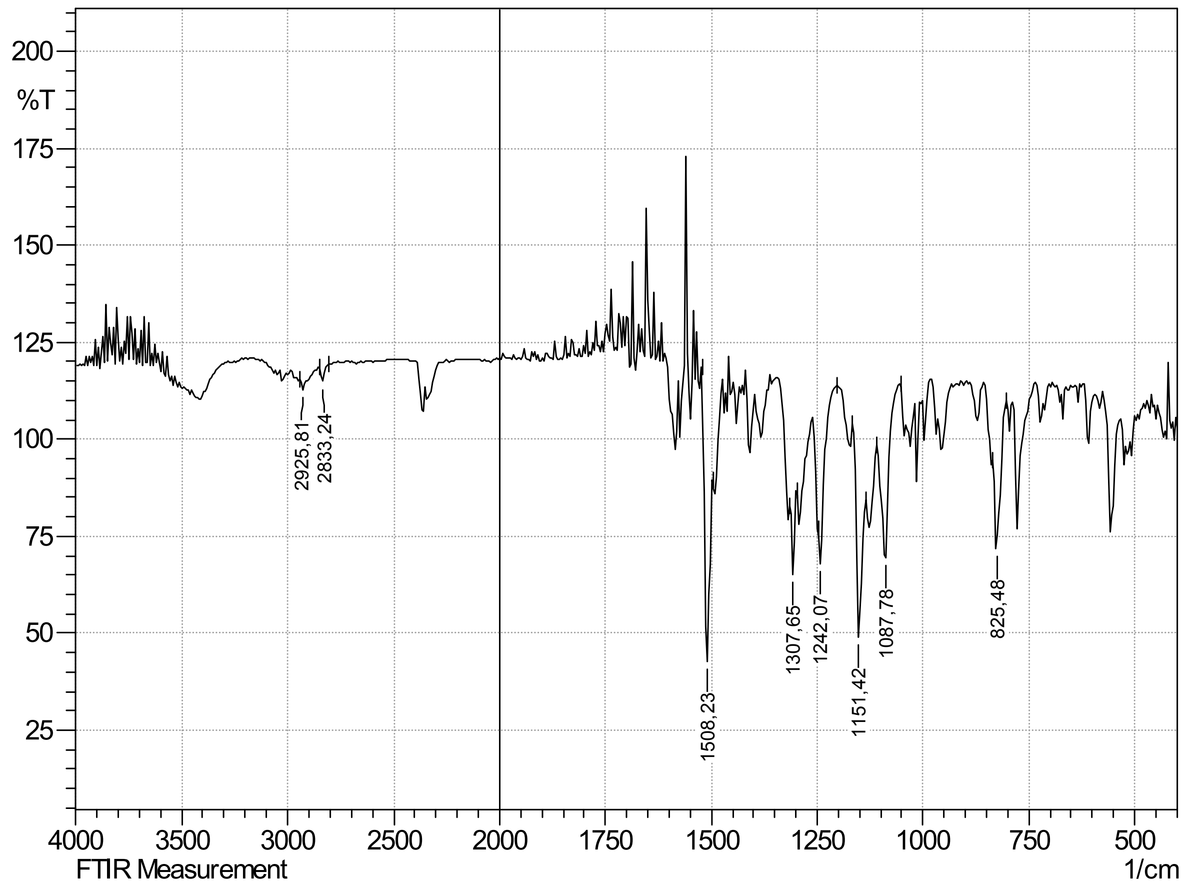

Figure S16. IR Spectrum of compound **2f**.

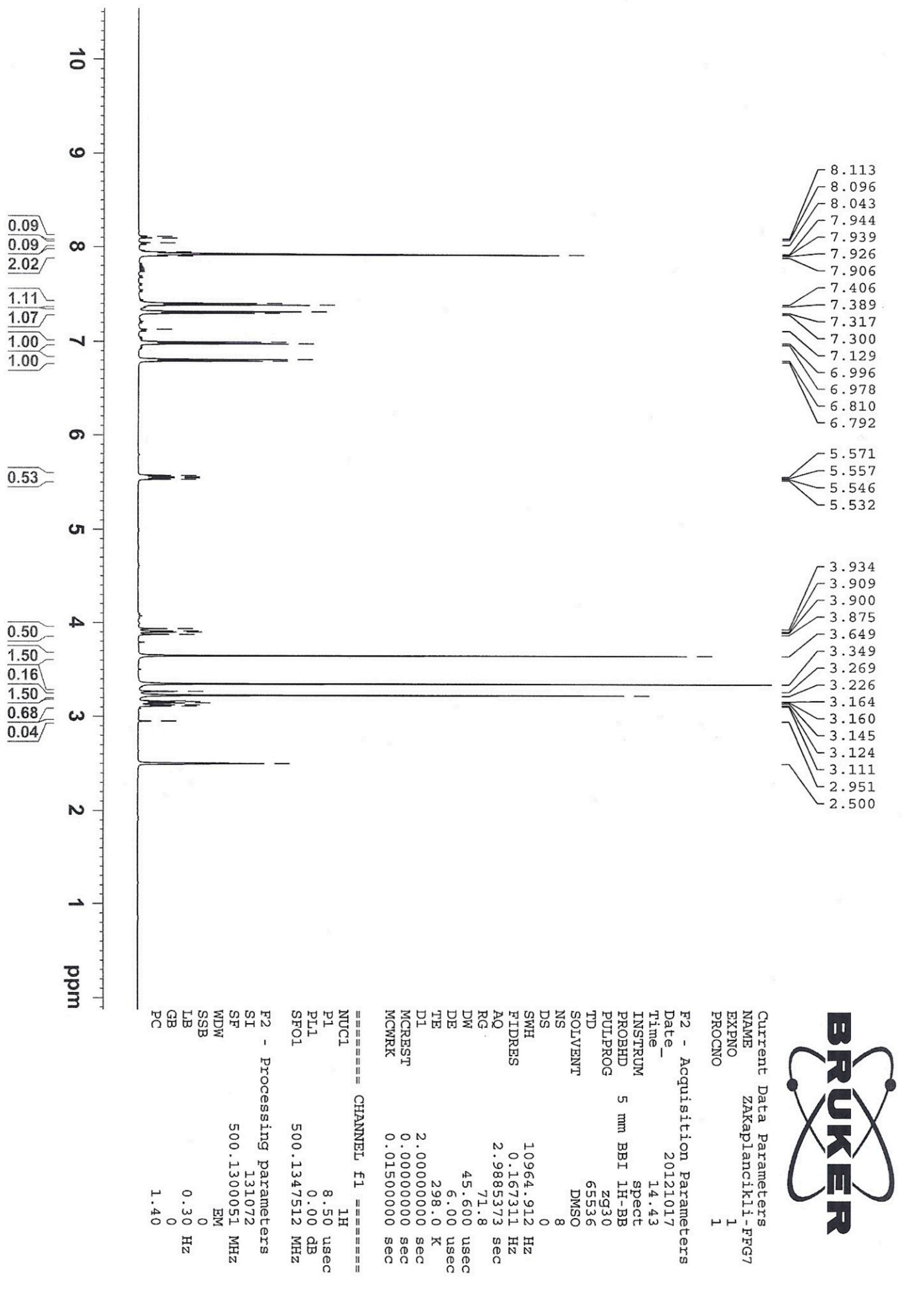

Figure S17. ^{1}H-NMR Spectrum of compound **2f**.

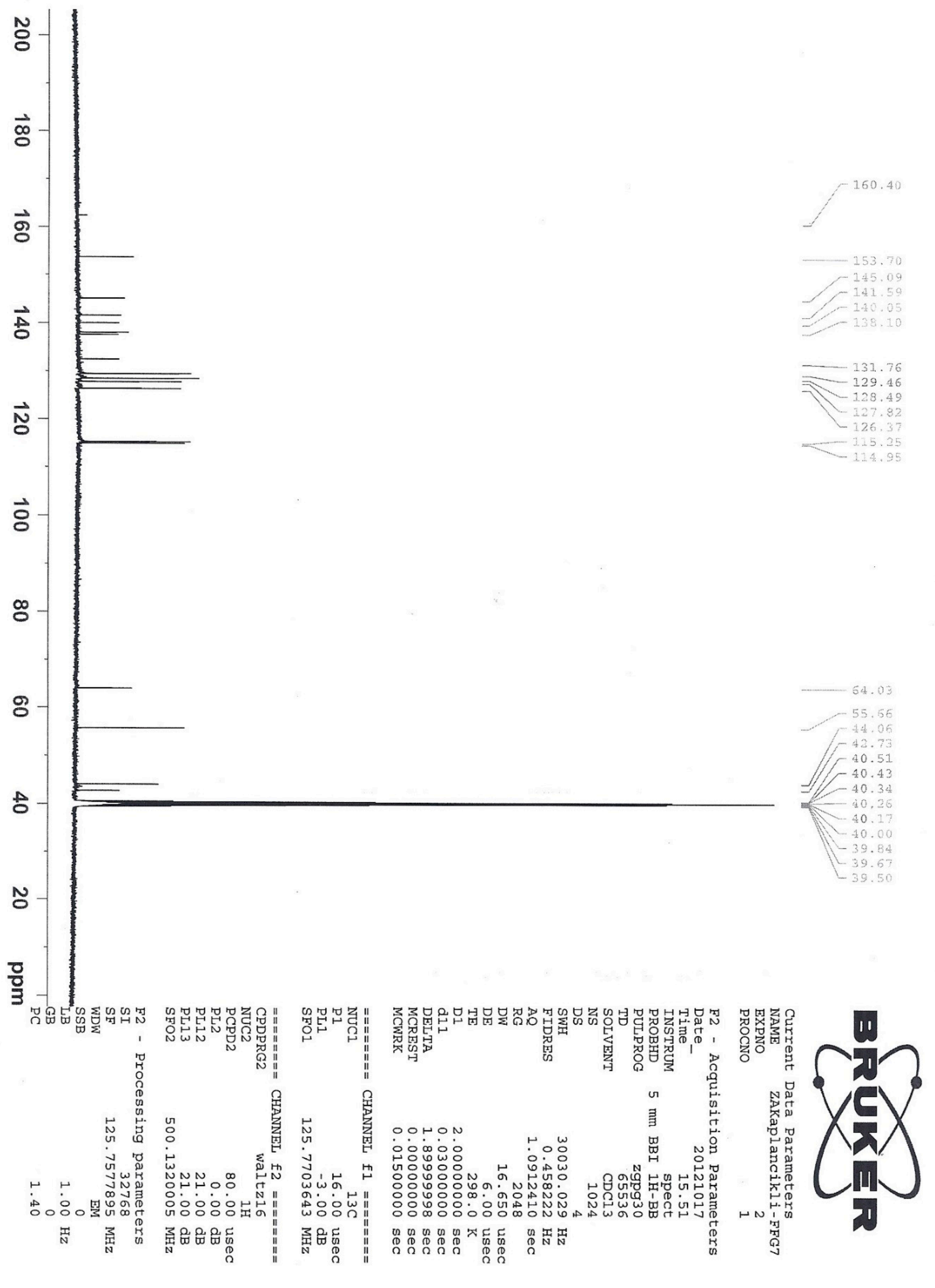

Figure S18. ^{13}C-NMR Spectrum of compound **2f**.

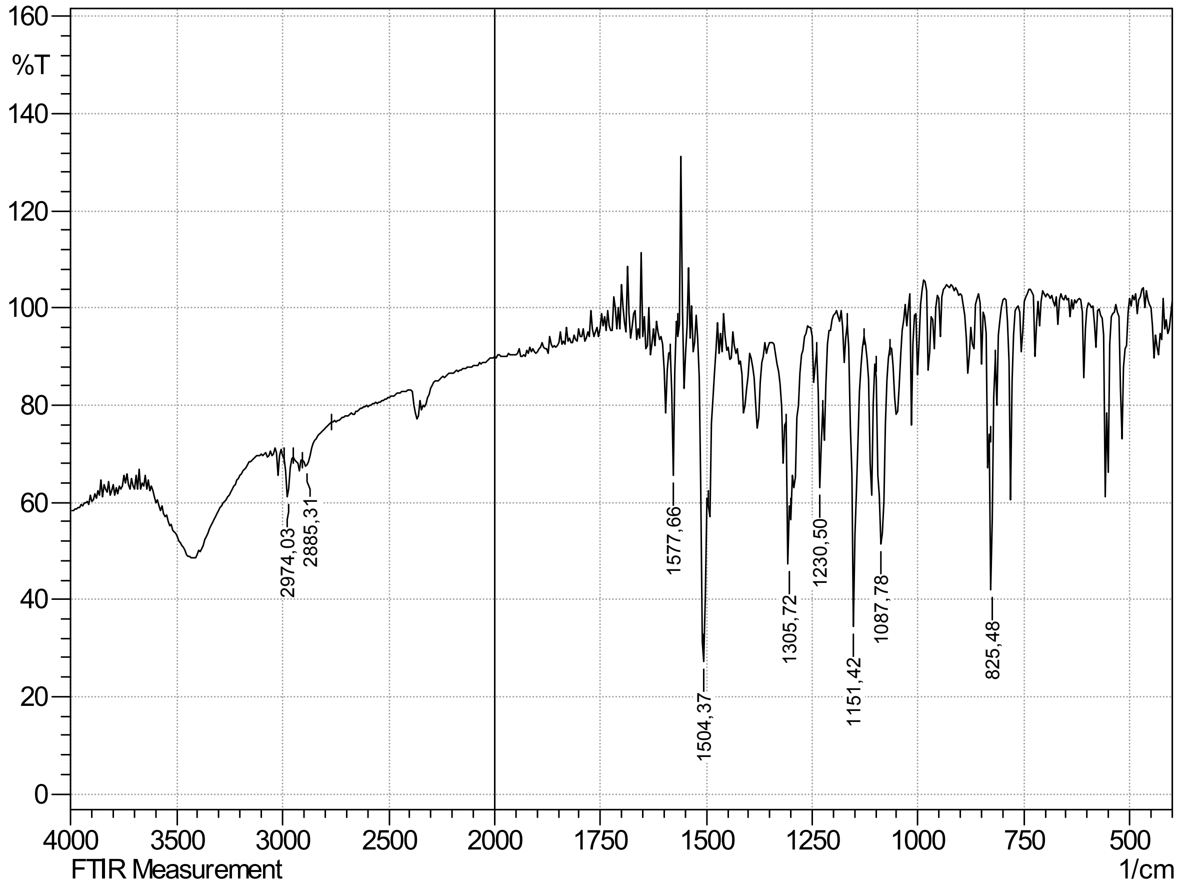

Figure S19. IR Spectrum of compound **2g**.

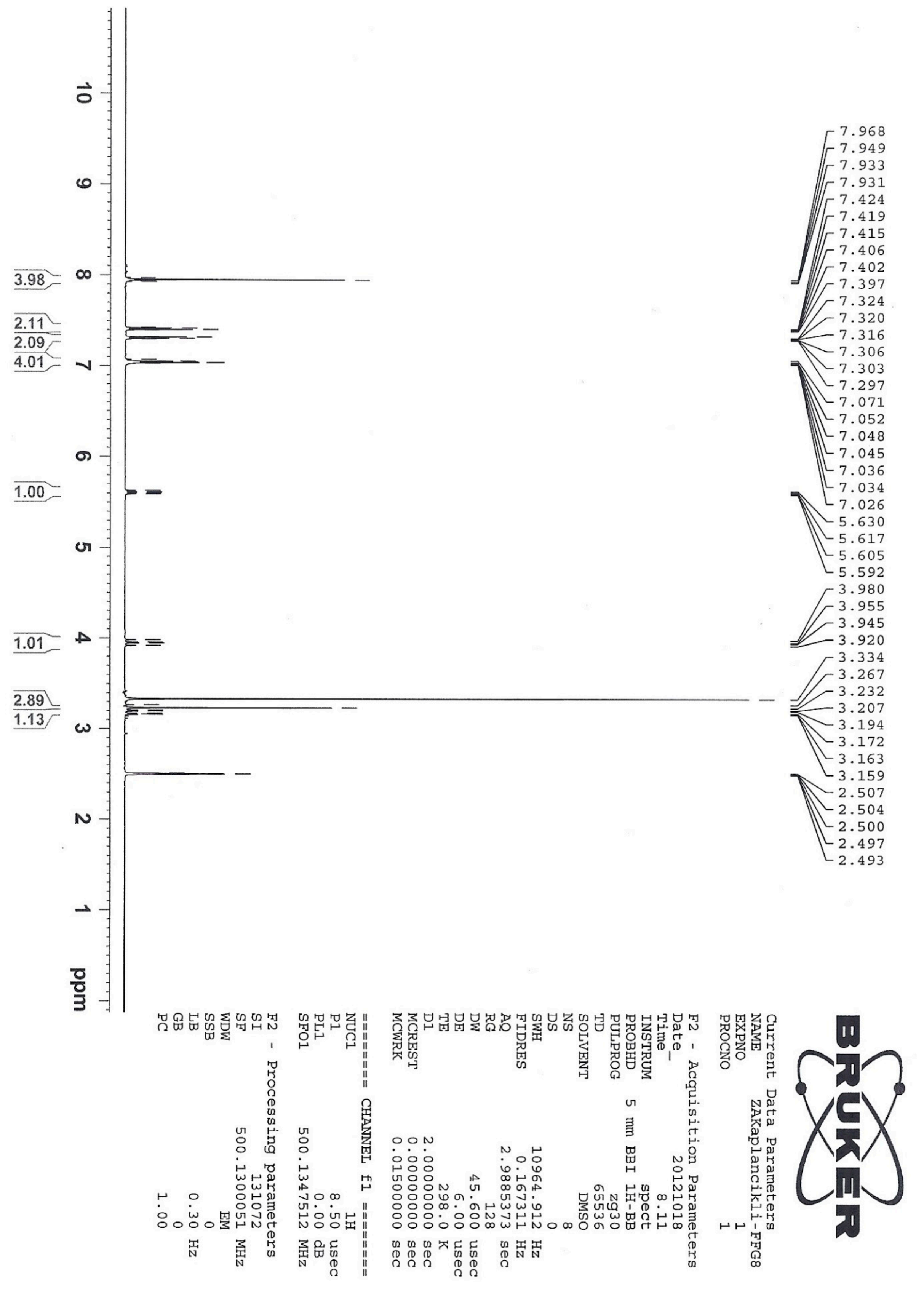

Figure S20. ^{1}H-NMR Spectrum of compound **2g**.

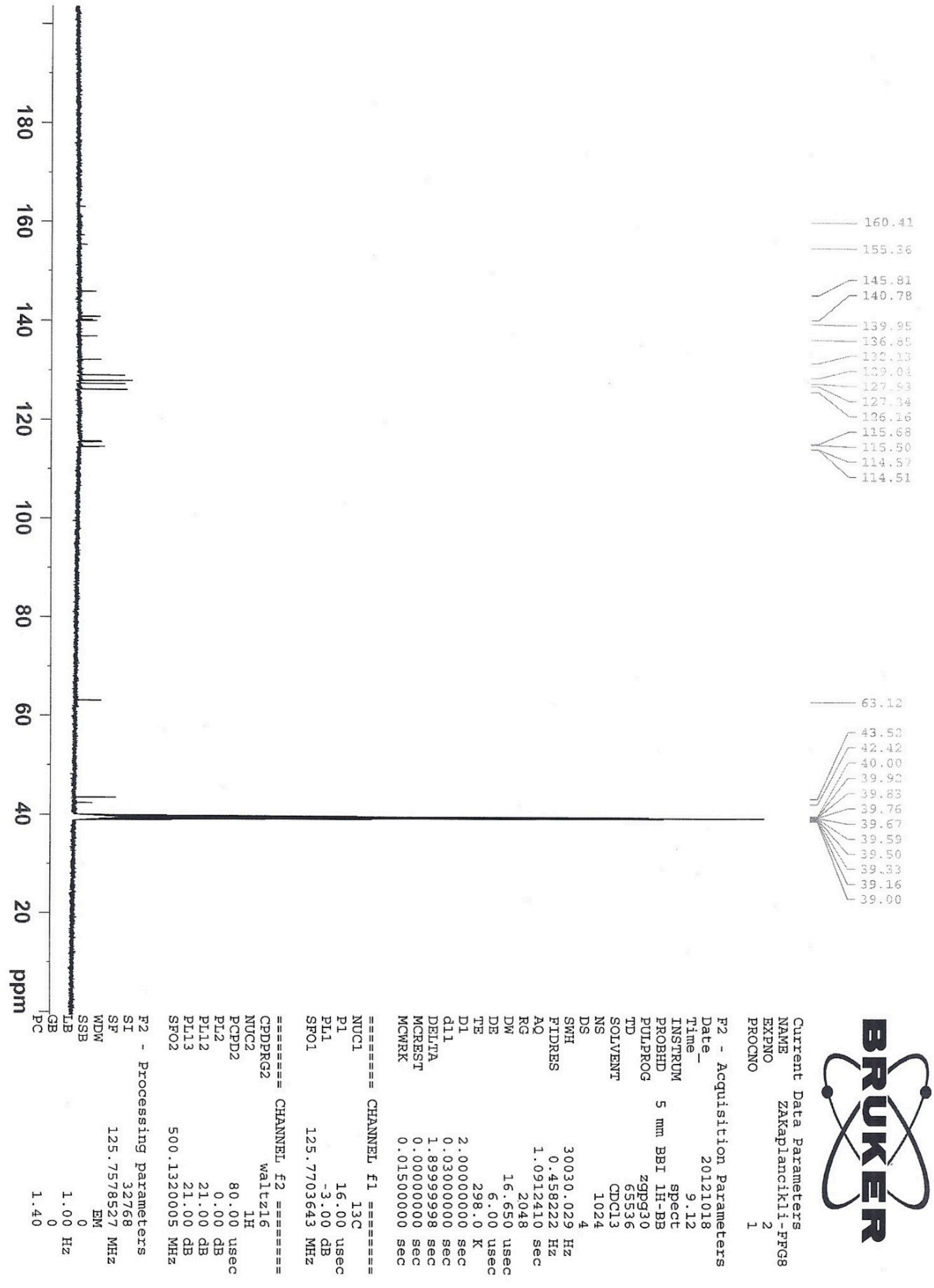

Figure S21. ^{13}C-NMR Spectrum of compound **2g**.

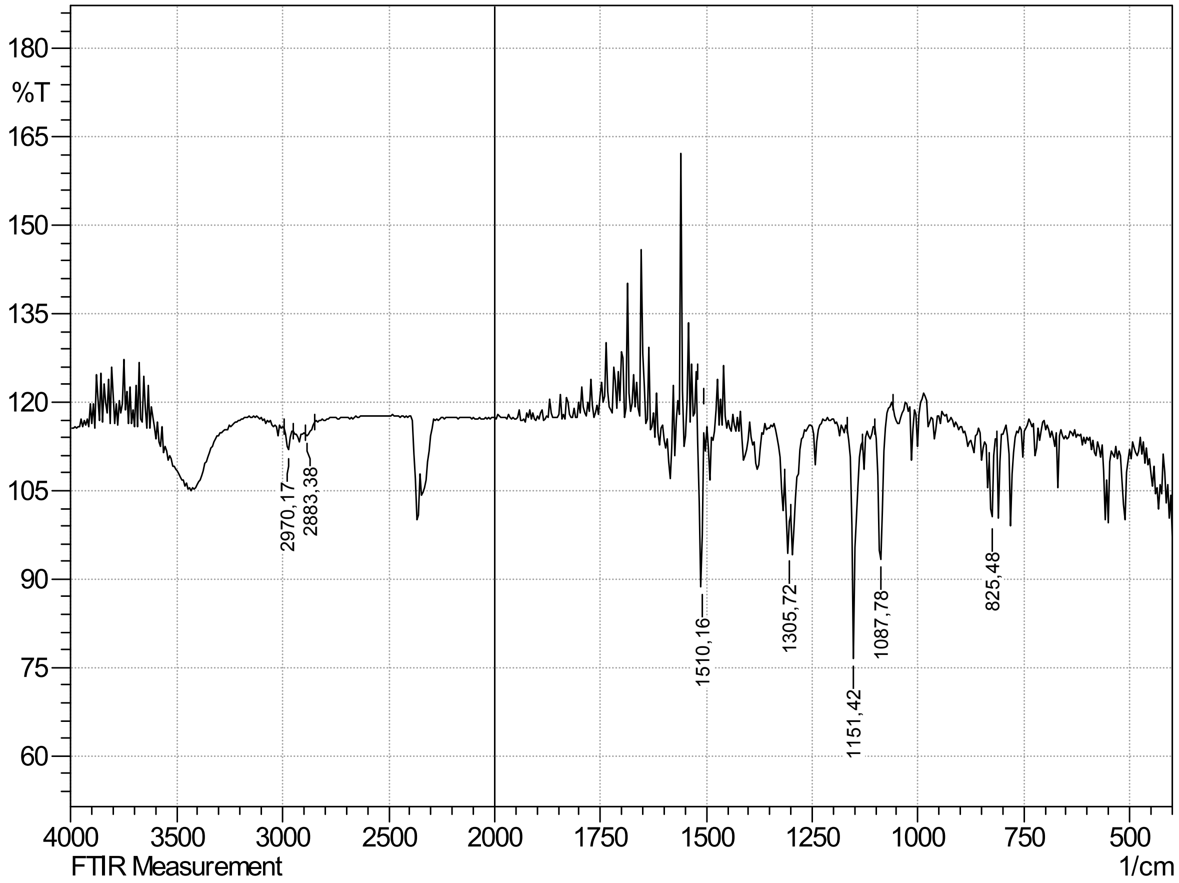

Figure S22. IR Spectrum of compound **2h**.

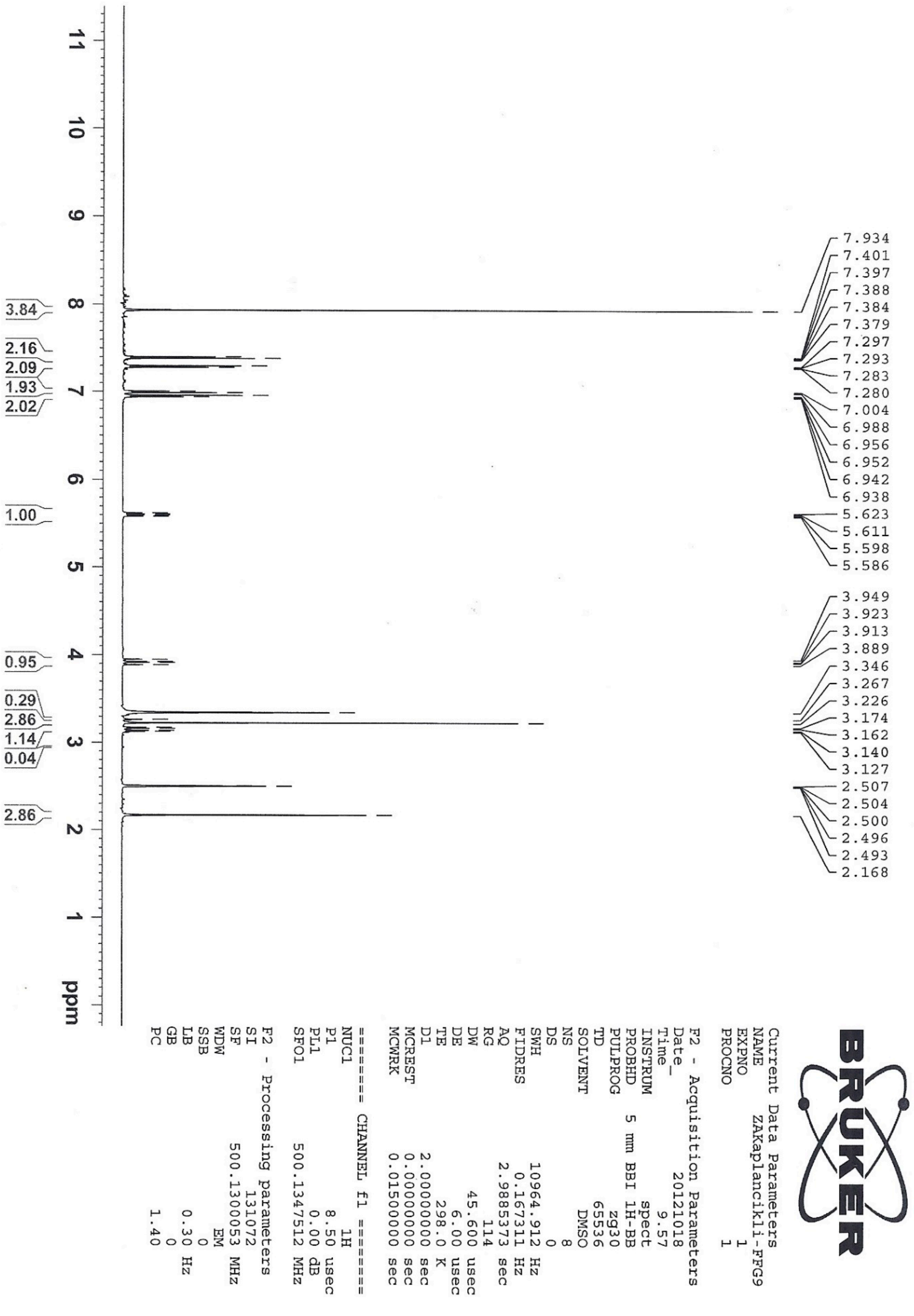

Figure S23. ^{1}H-NMR Spectrum of compound **2h**.

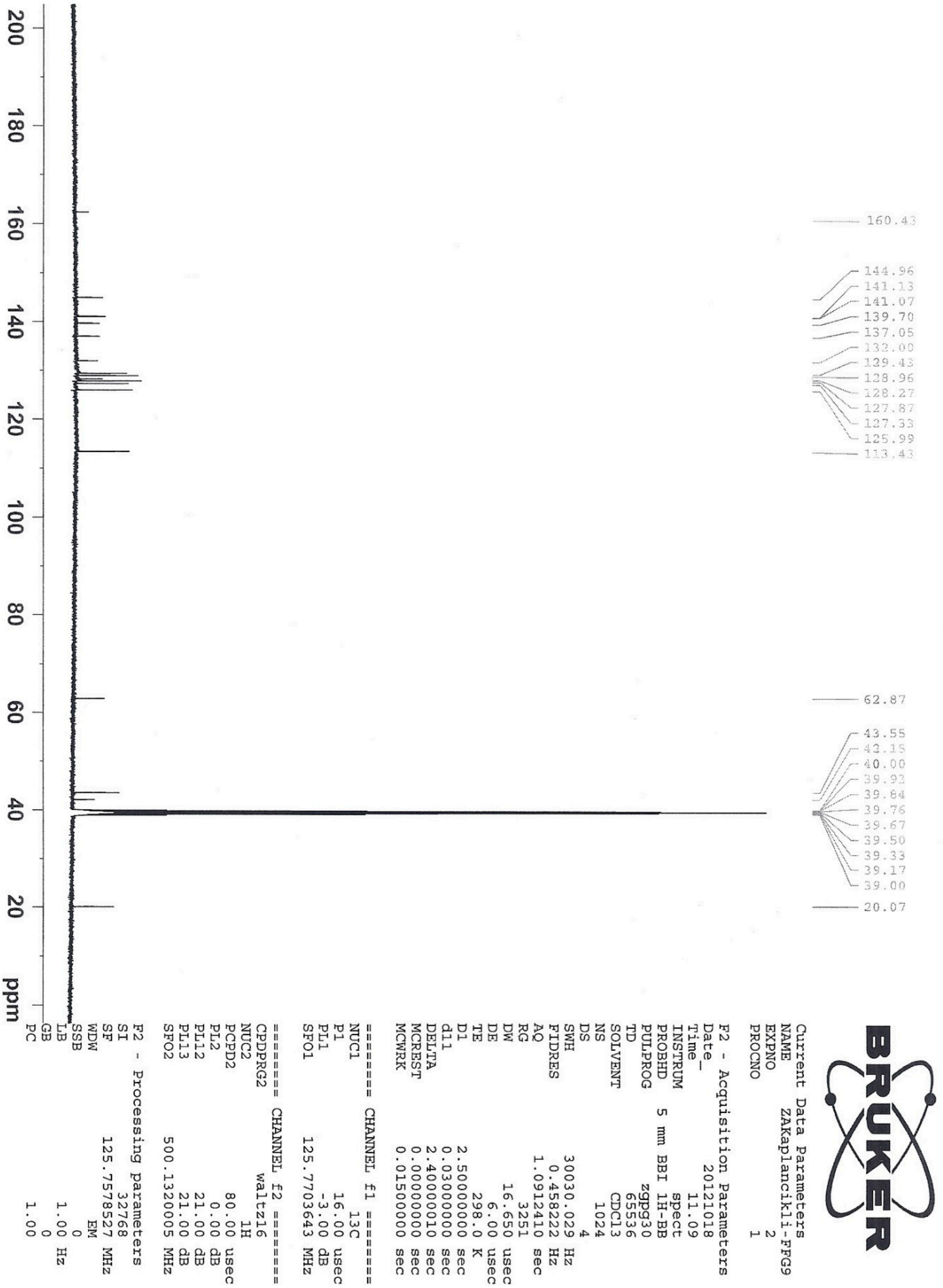

Figure S24. ^{13}C-NMR Spectrum of compound **2h**.

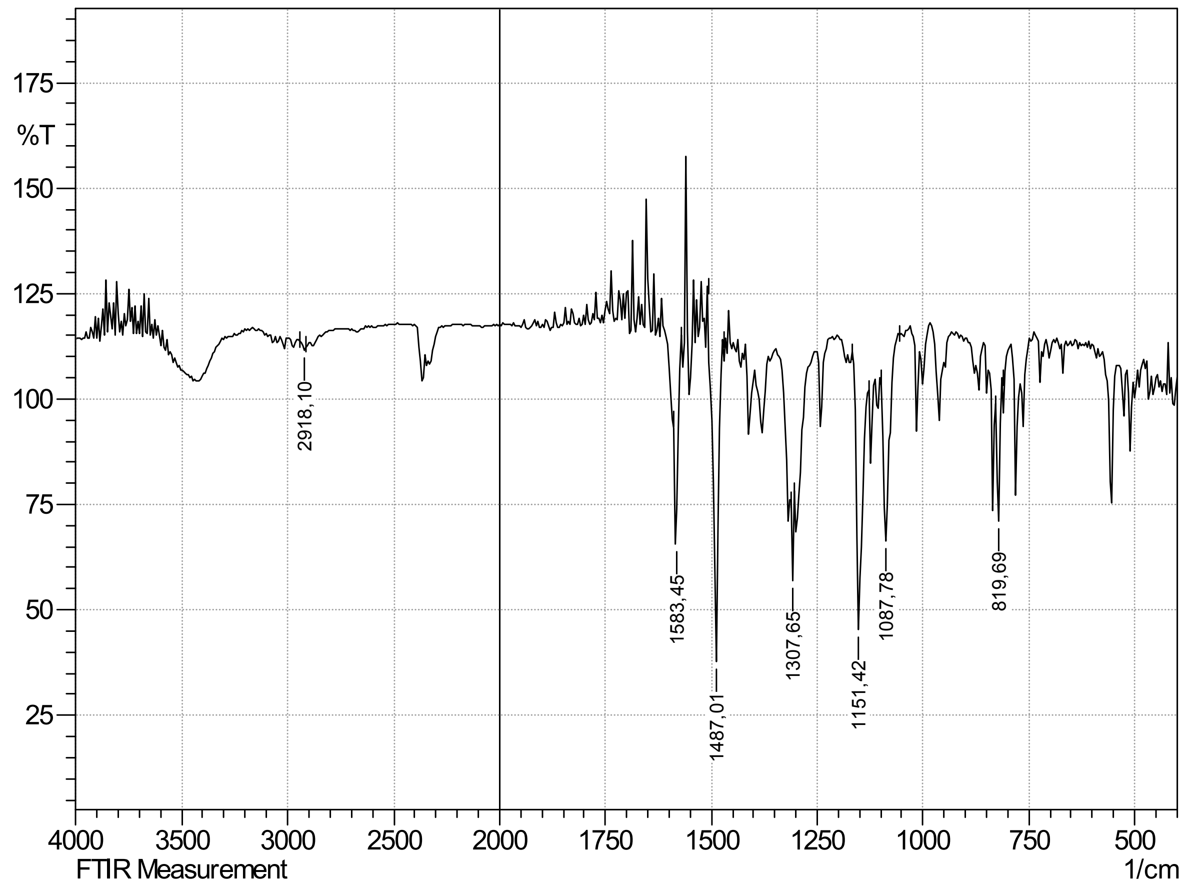

Figure S25. IR Spectrum of compound **2i**.

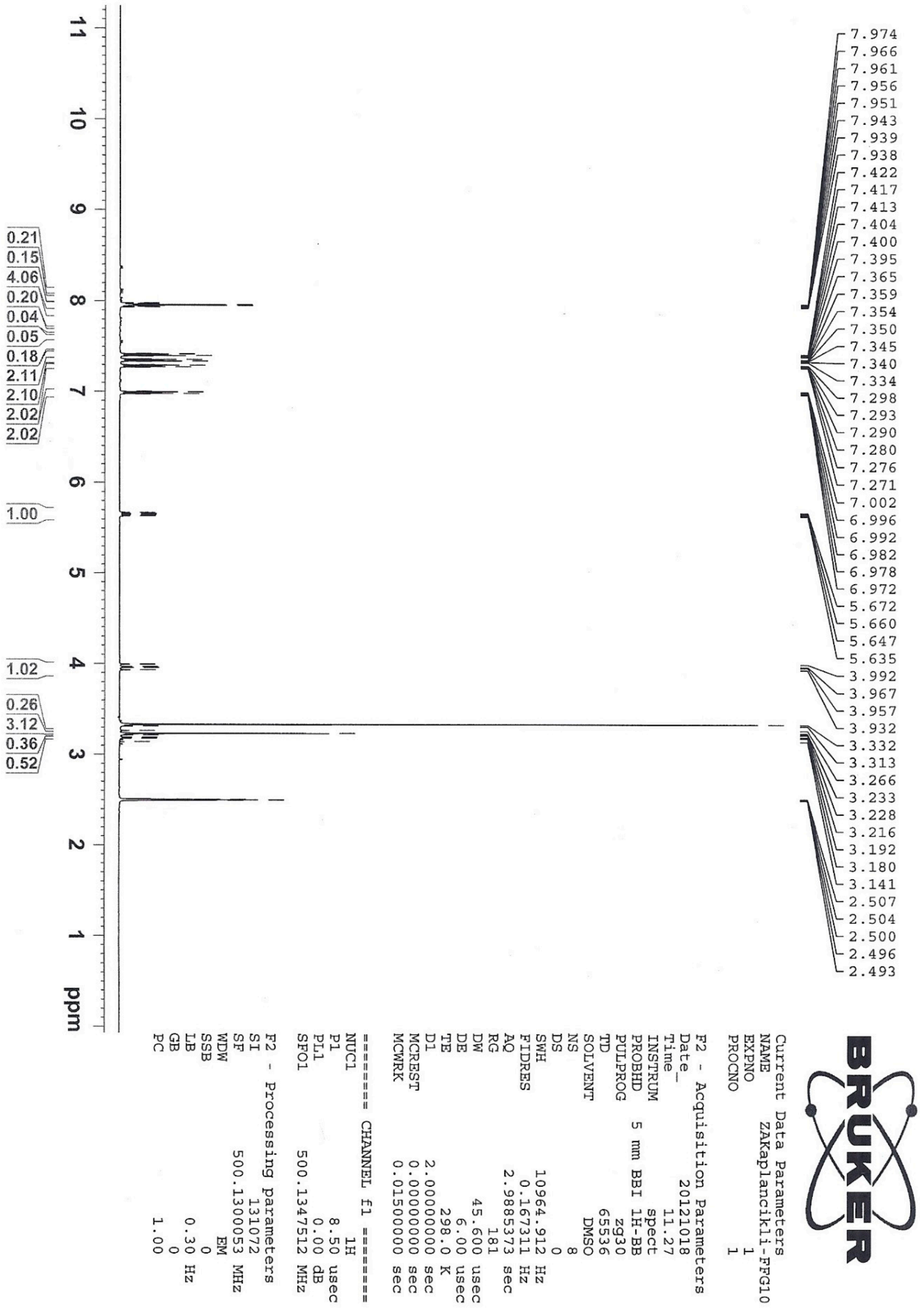

Figure S26. ^{1}H-NMR Spectrum of compound **2i**.

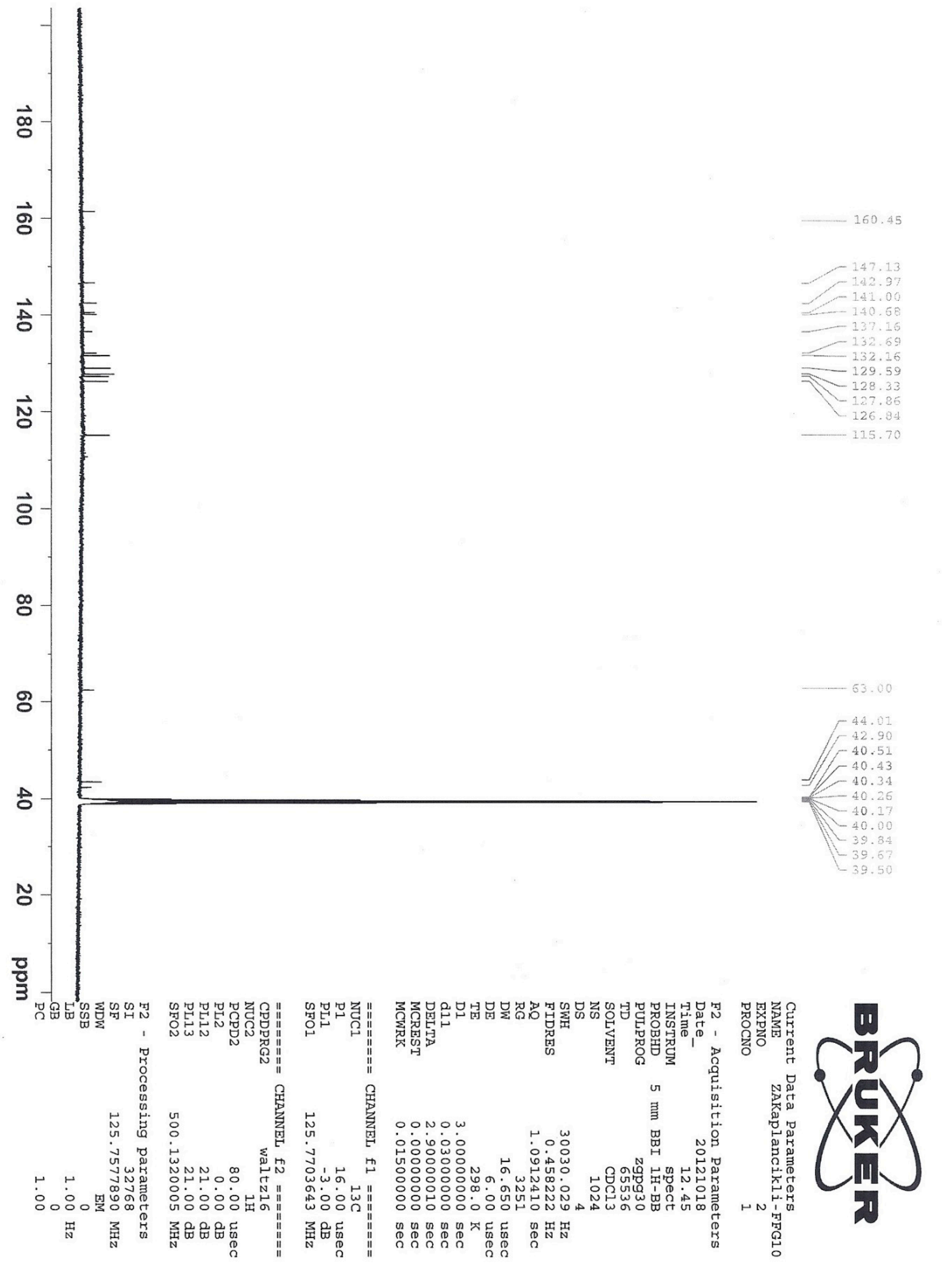

Figure S27. ^{13}C-NMR Spectrum of compound **2i**.

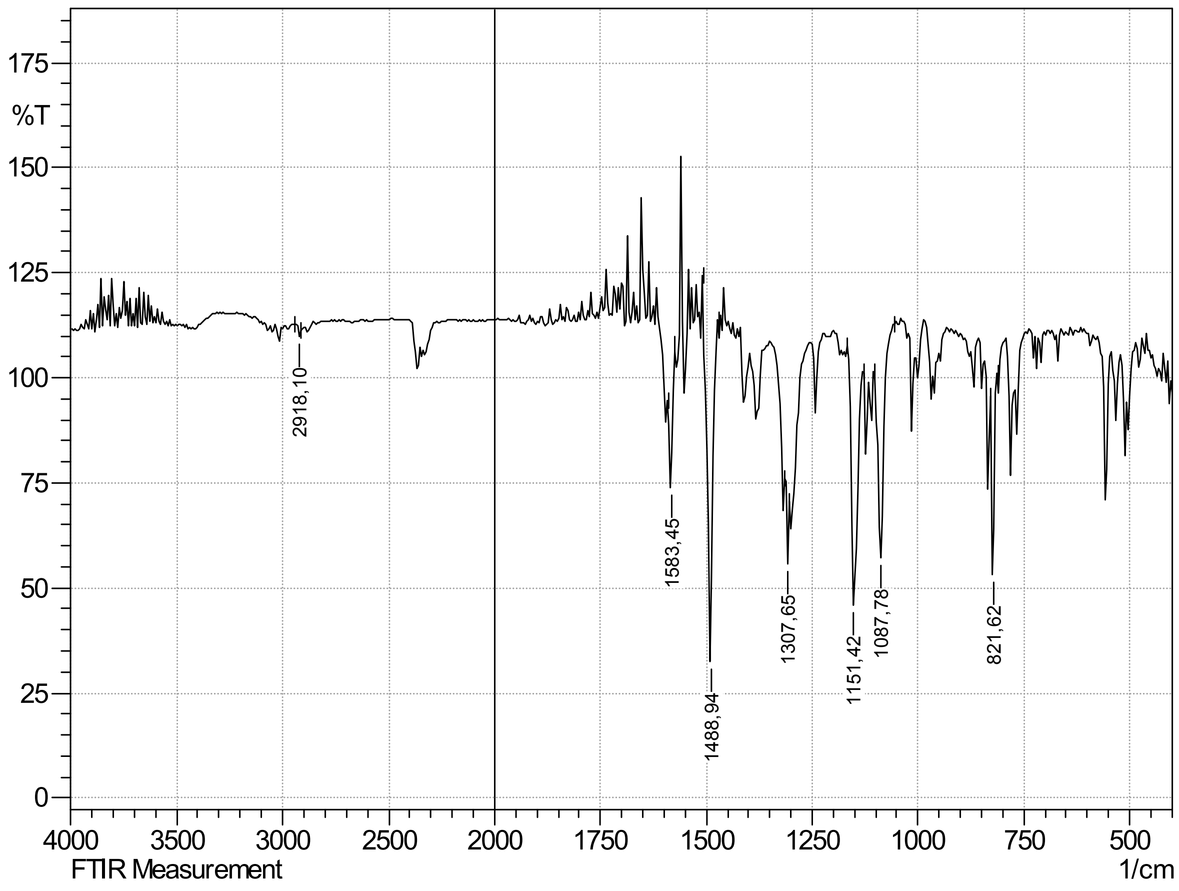

Figure S28. IR Spectrum of compound **2j**.

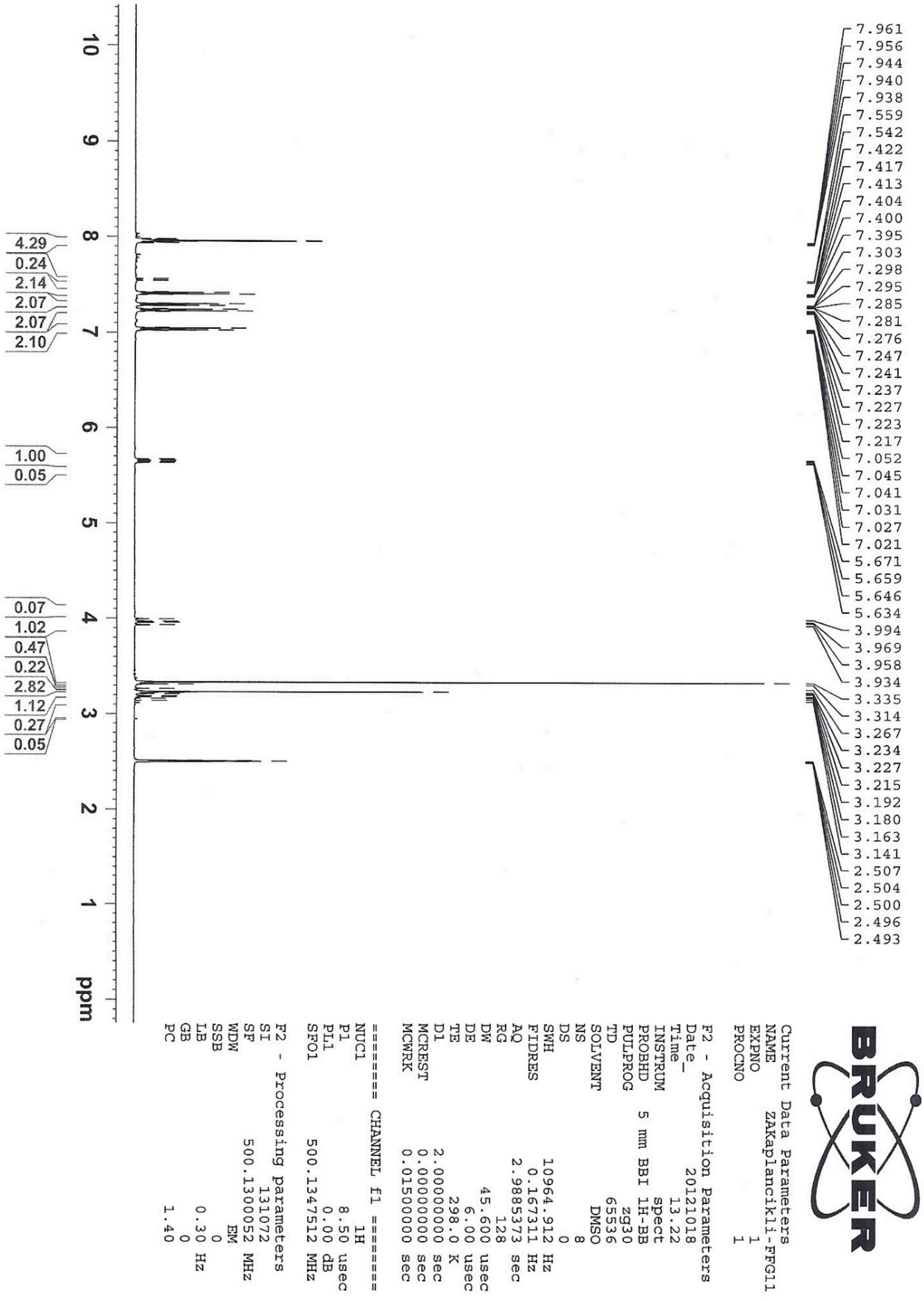

Figure S29. ^{1}H-NMR Spectrum of compound **2j**.

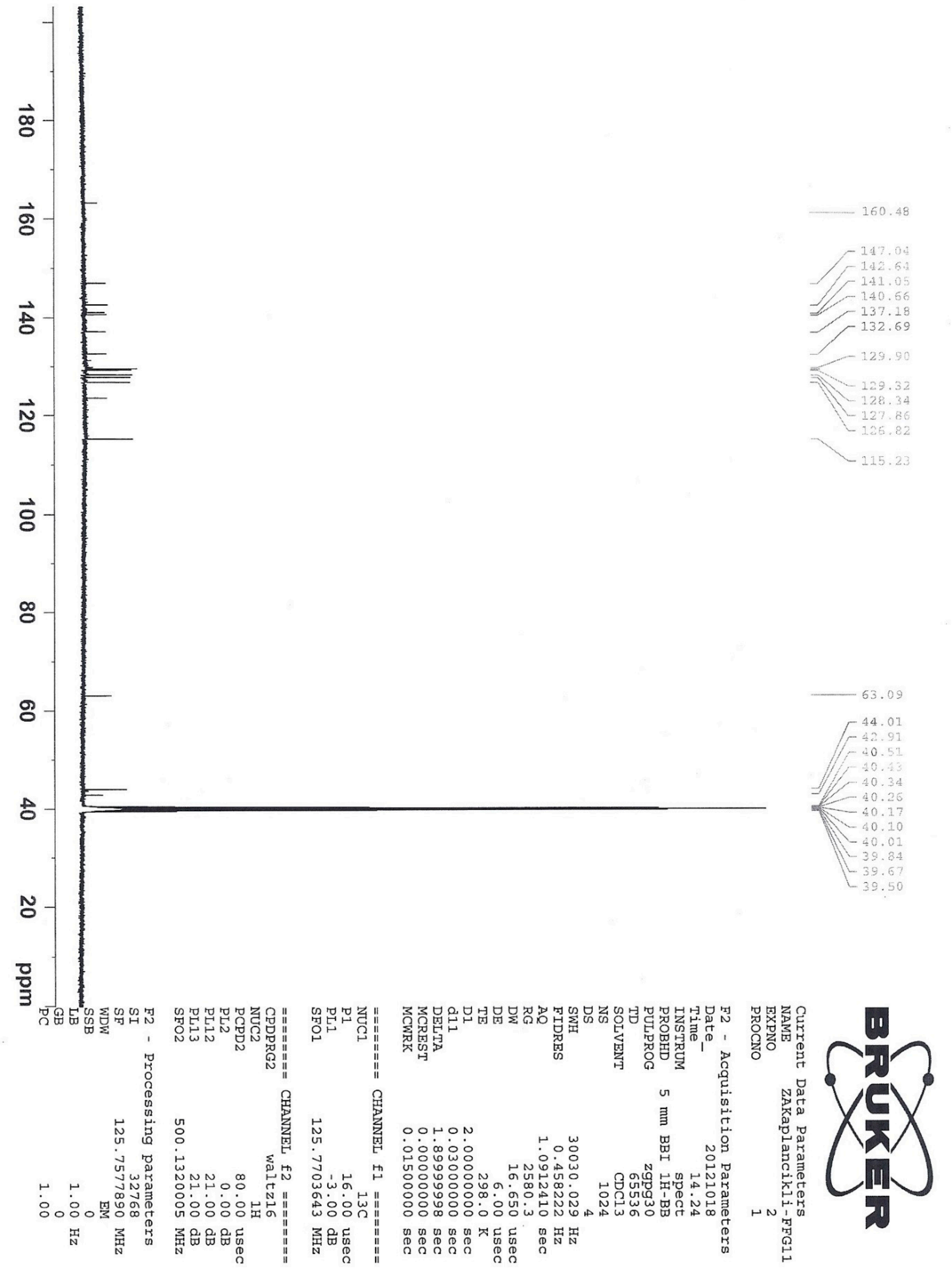

Figure S30. ^{13}C-NMR Spectrum of compound **2j**.

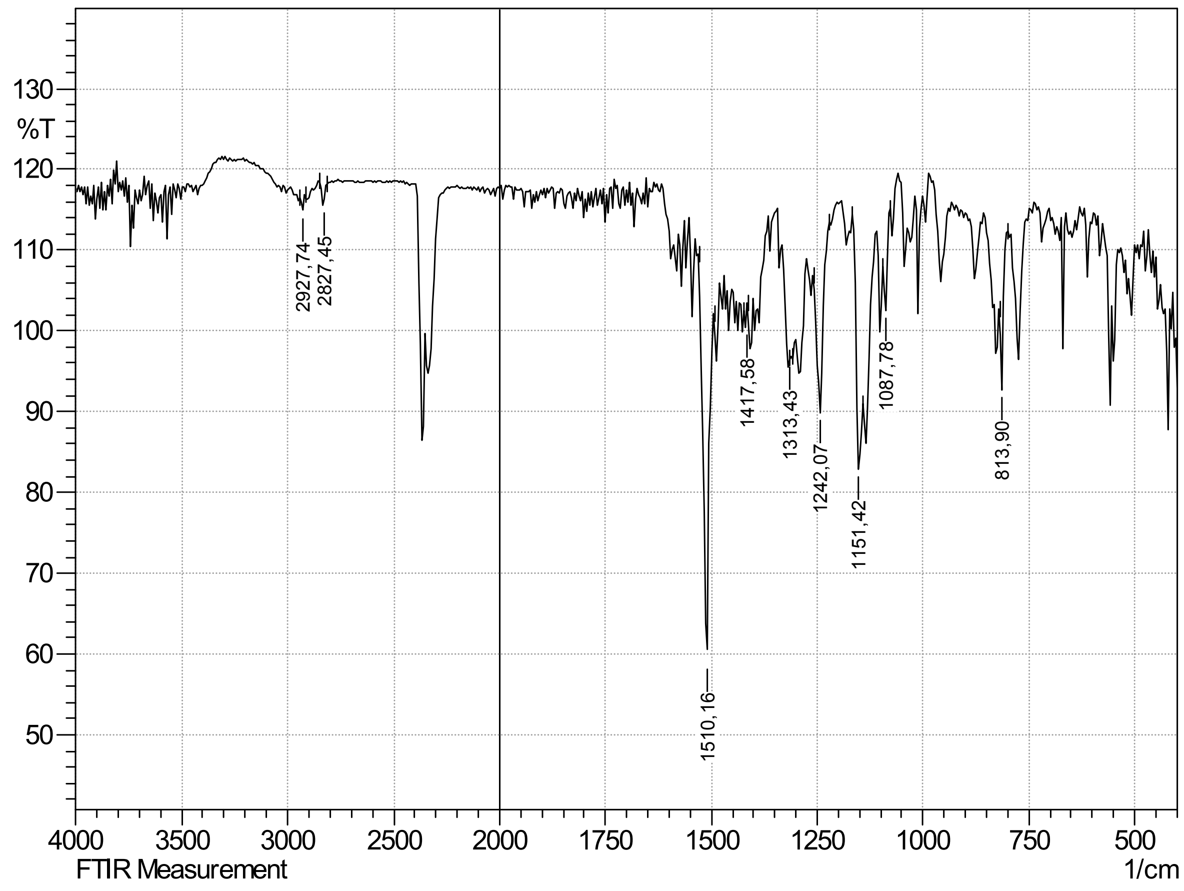

Figure S31. IR Spectrum of compound **2k**.

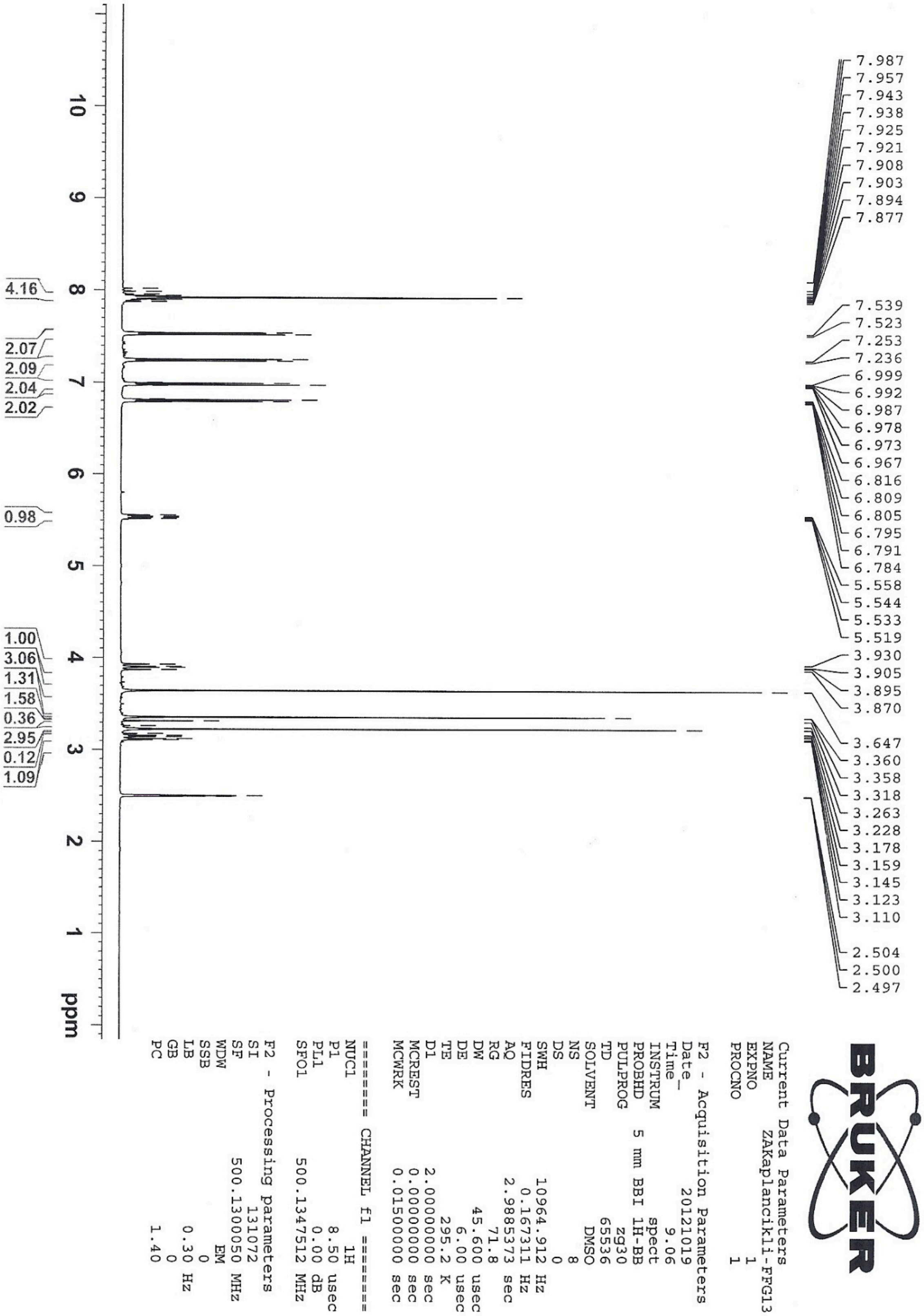

Figure S32. ^{1}H-NMR Spectrum of compound **2k**.

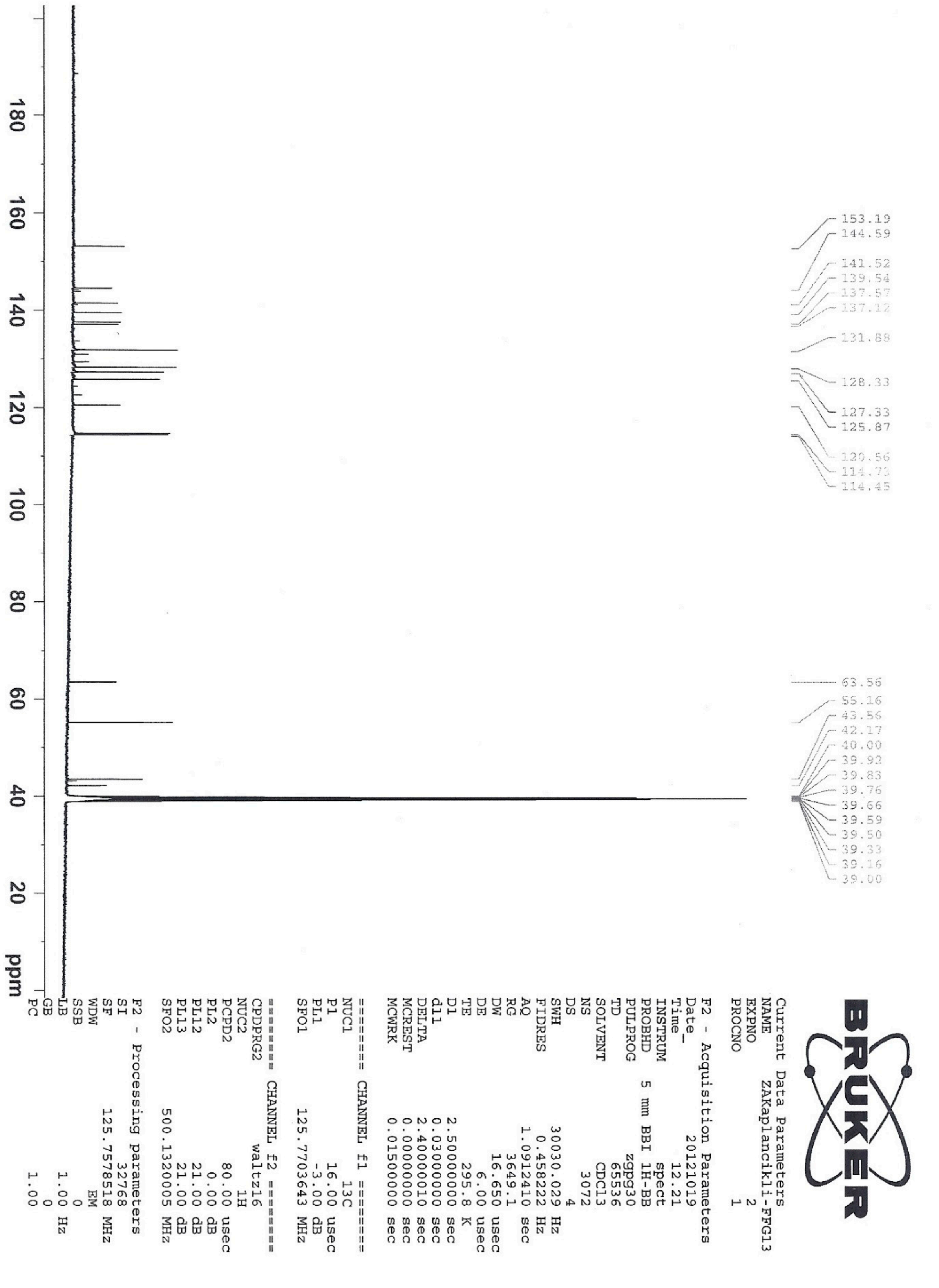

Figure S33. ^{13}C-NMR Spectrum of compound **2k**.

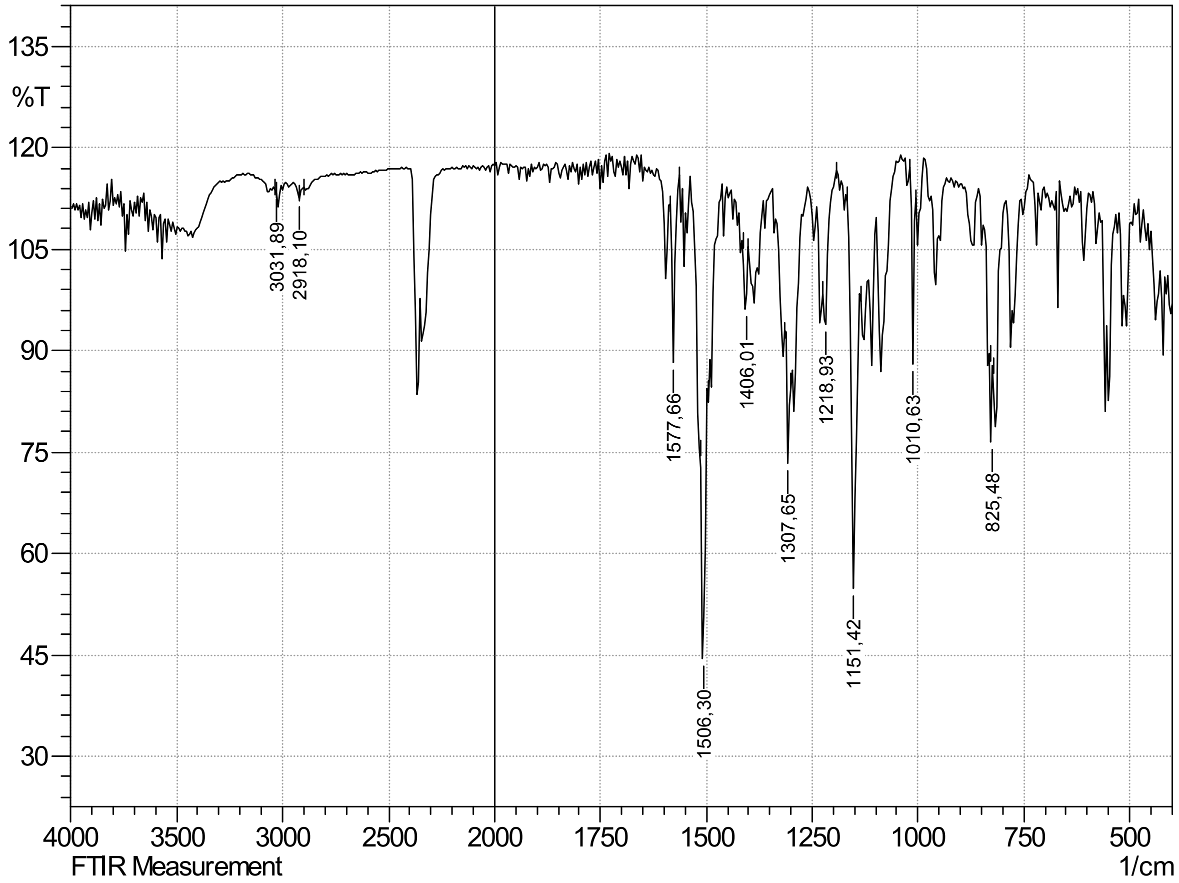

Figure S34. IR Spectrum of compound **2l**.

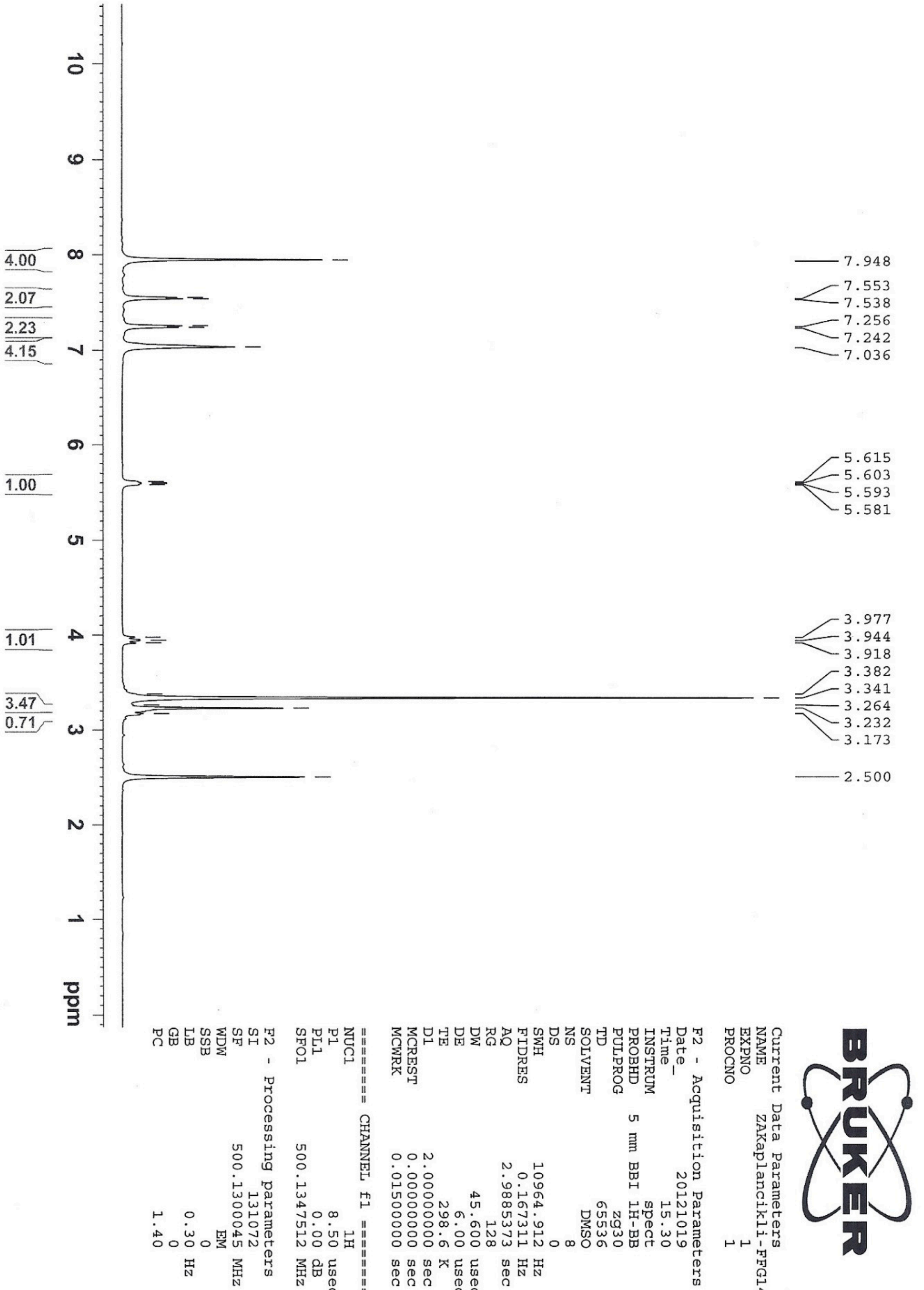

Figure S35. ^{1}H-NMR Spectrum of compound **2l**.

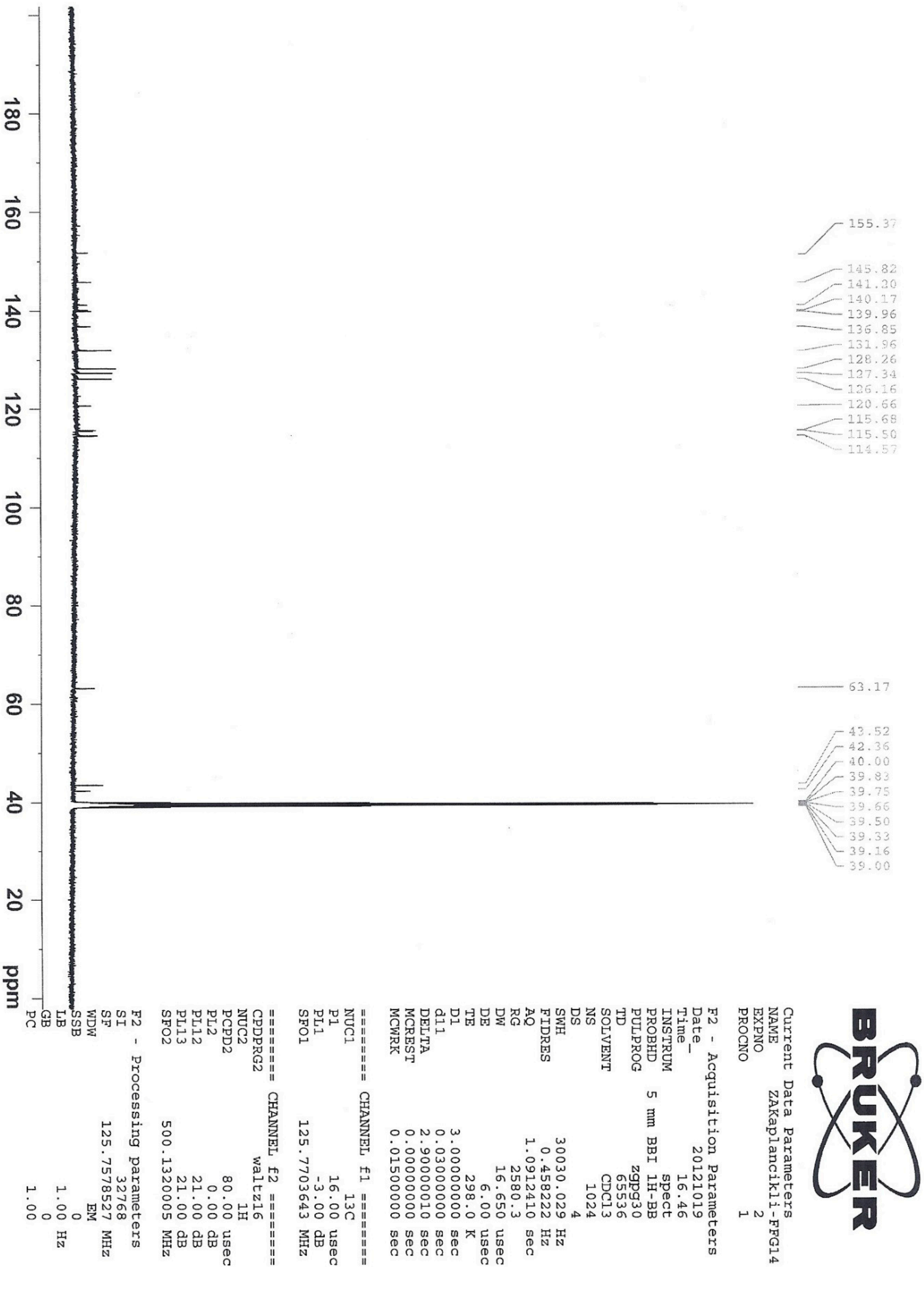

Figure S36. ^{13}C-NMR Spectrum of compound **2l**.

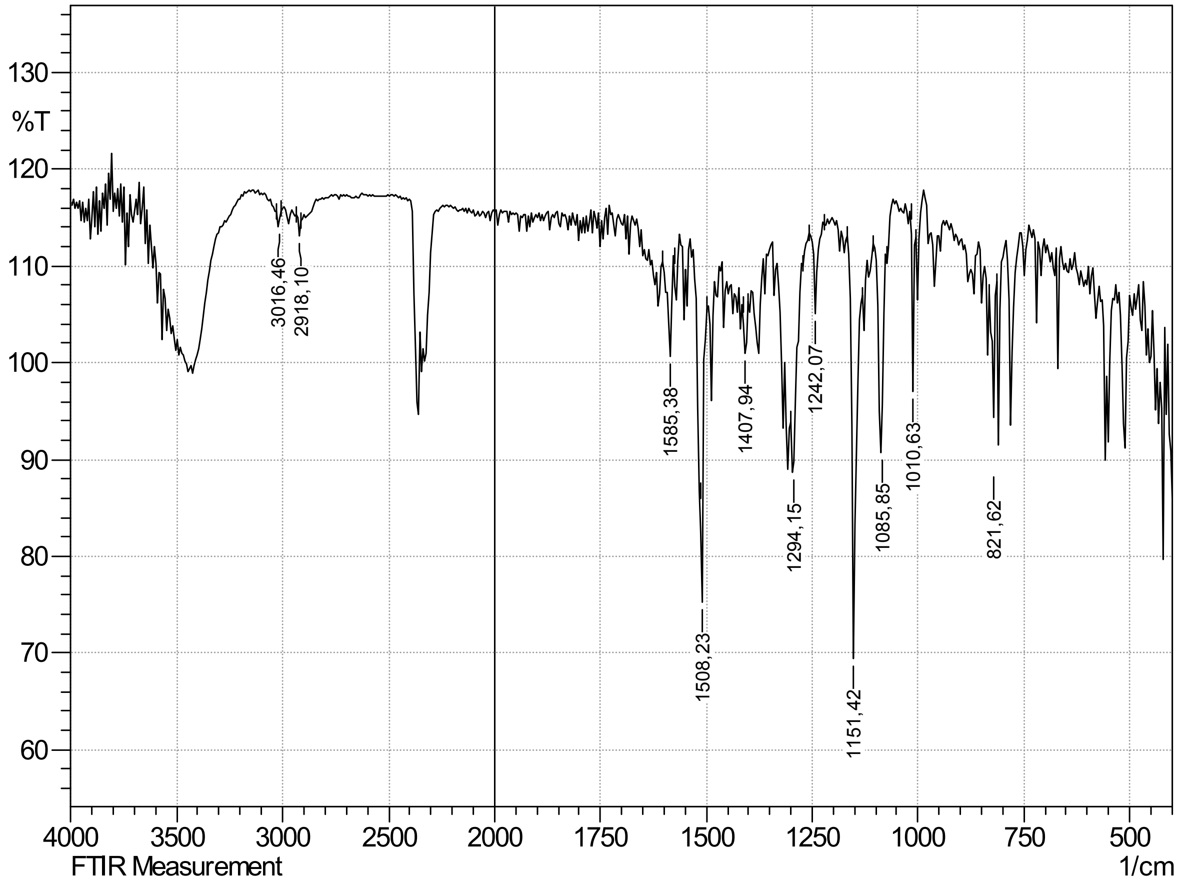

Figure S37. IR Spectrum of compound **2m**.

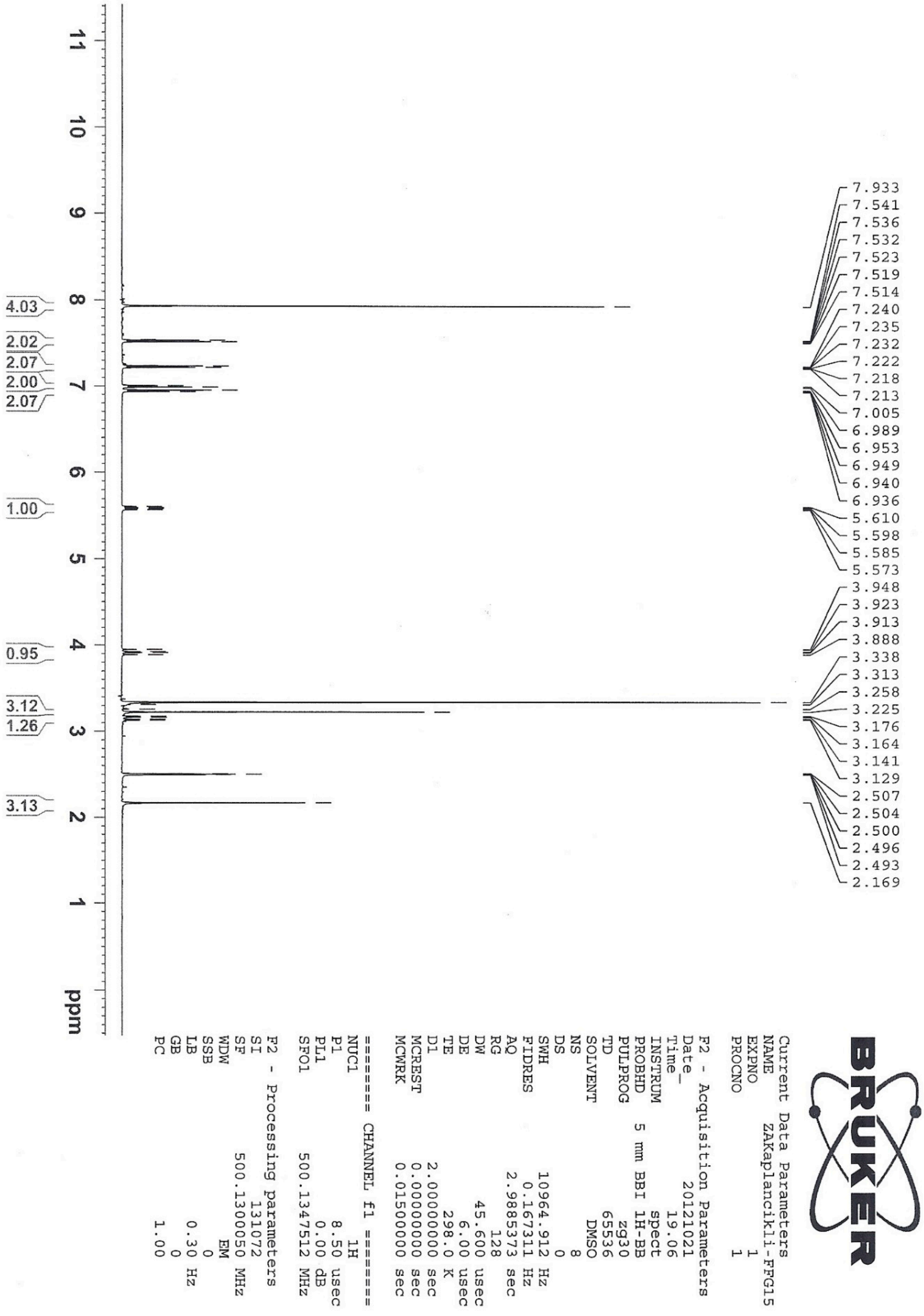

Figure S38. ^{1}H-NMR Spectrum of compound **2m**.

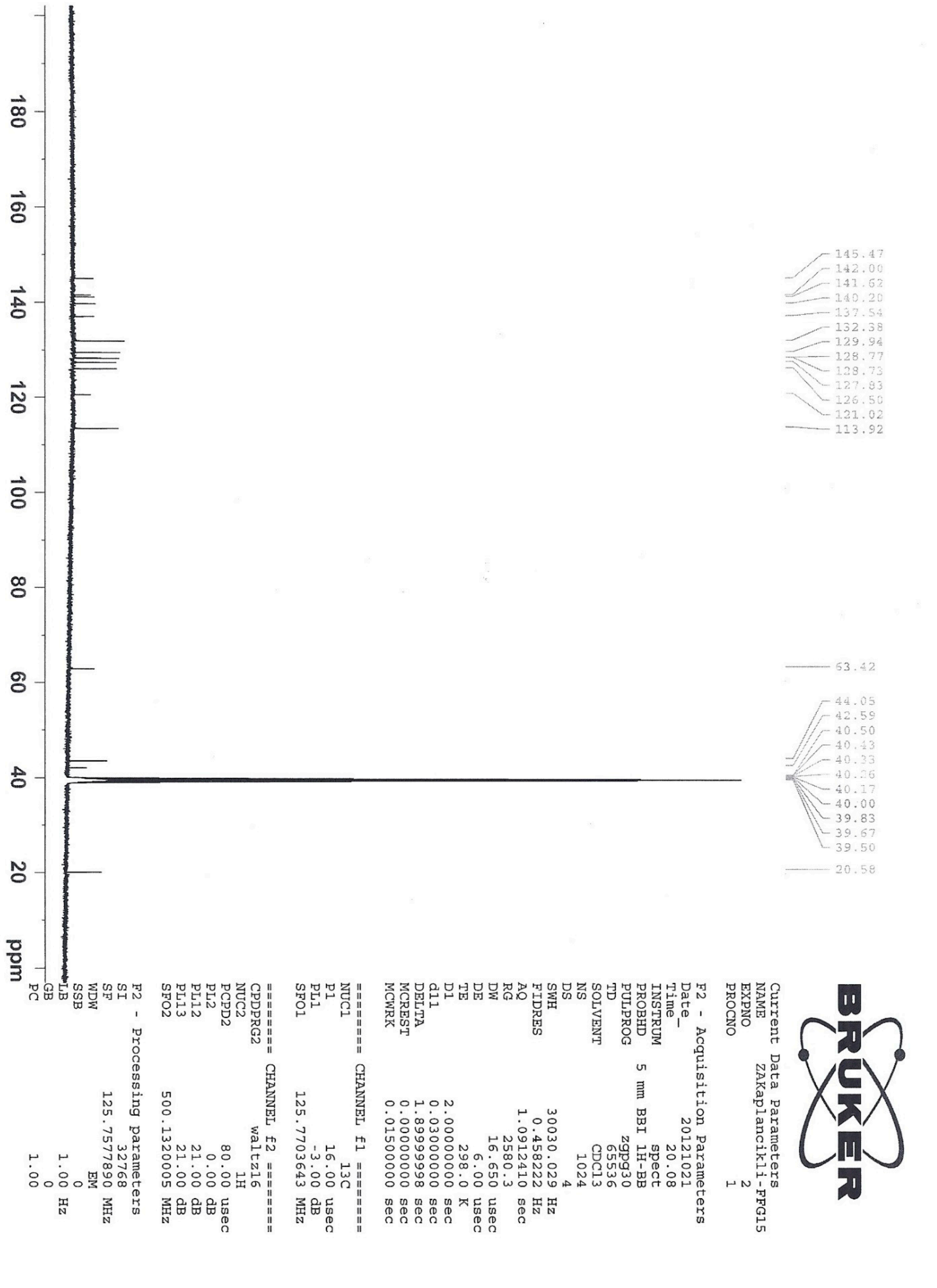

Figure S39. ^{13}C-NMR Spectrum of compound **2m**.

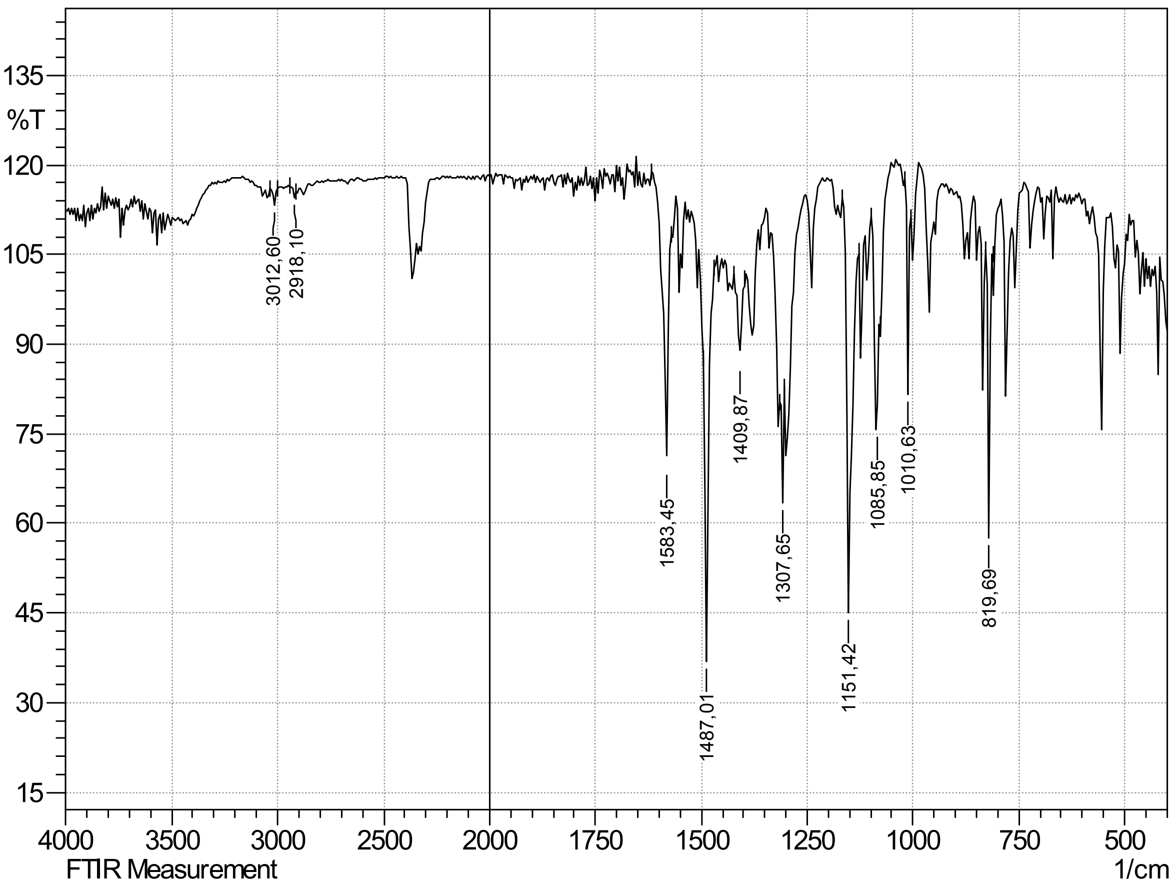

Figure S40. IR Spectrum of compound **2n**.

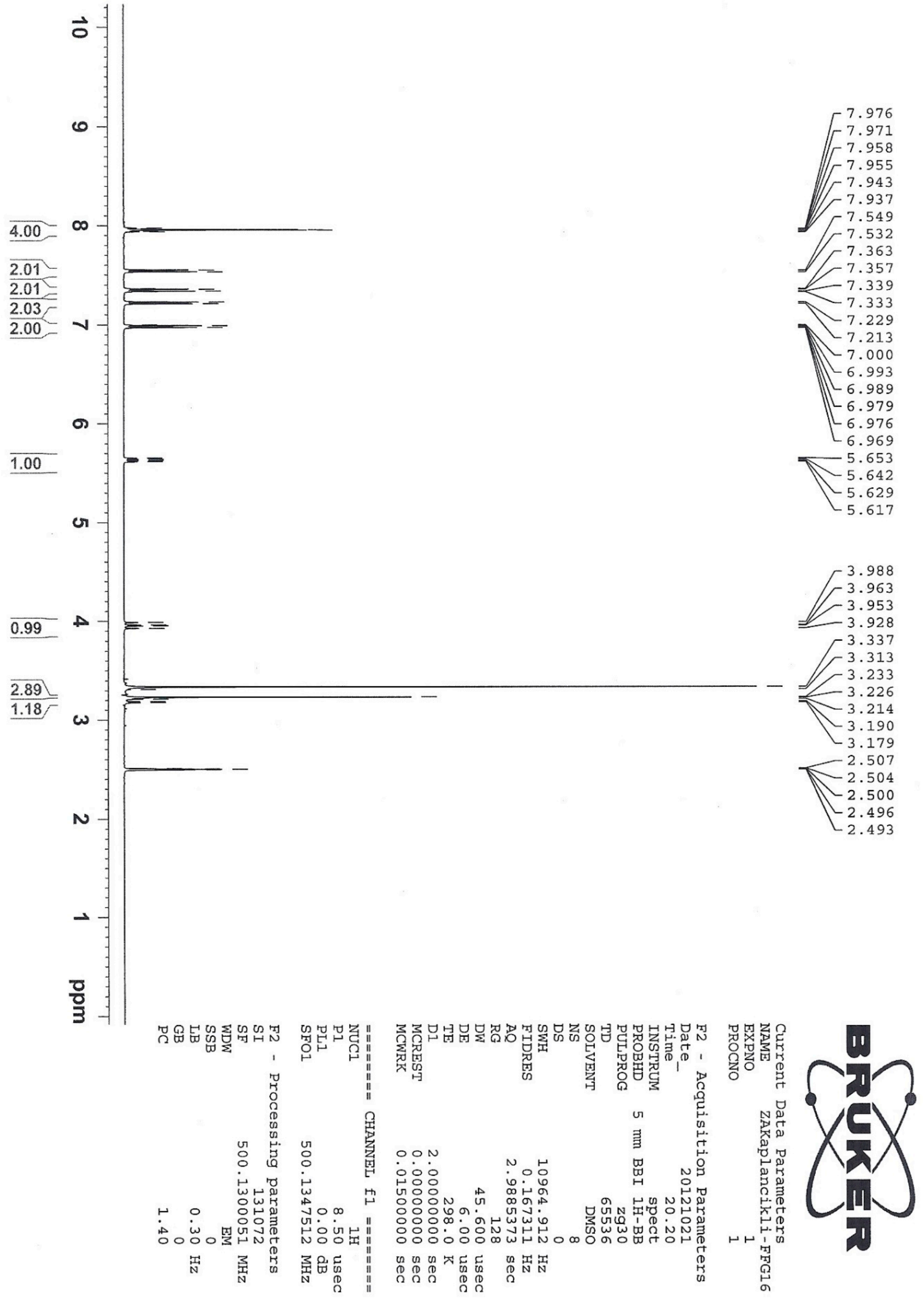

Figure S41. ^{1}H-NMR Spectrum of compound **2n**.

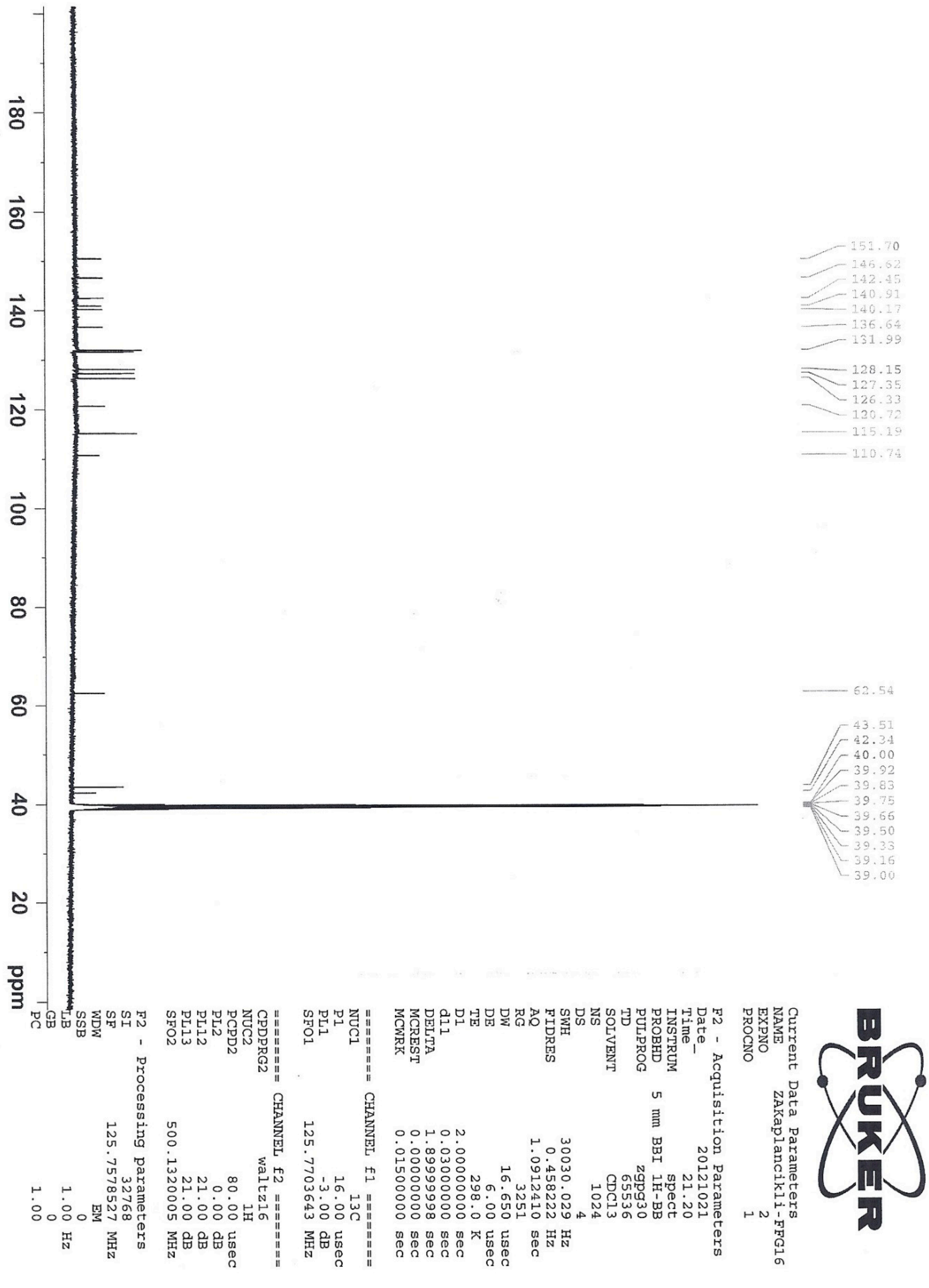

Figure S42. ^{13}C-NMR Spectrum of compound **2n**.

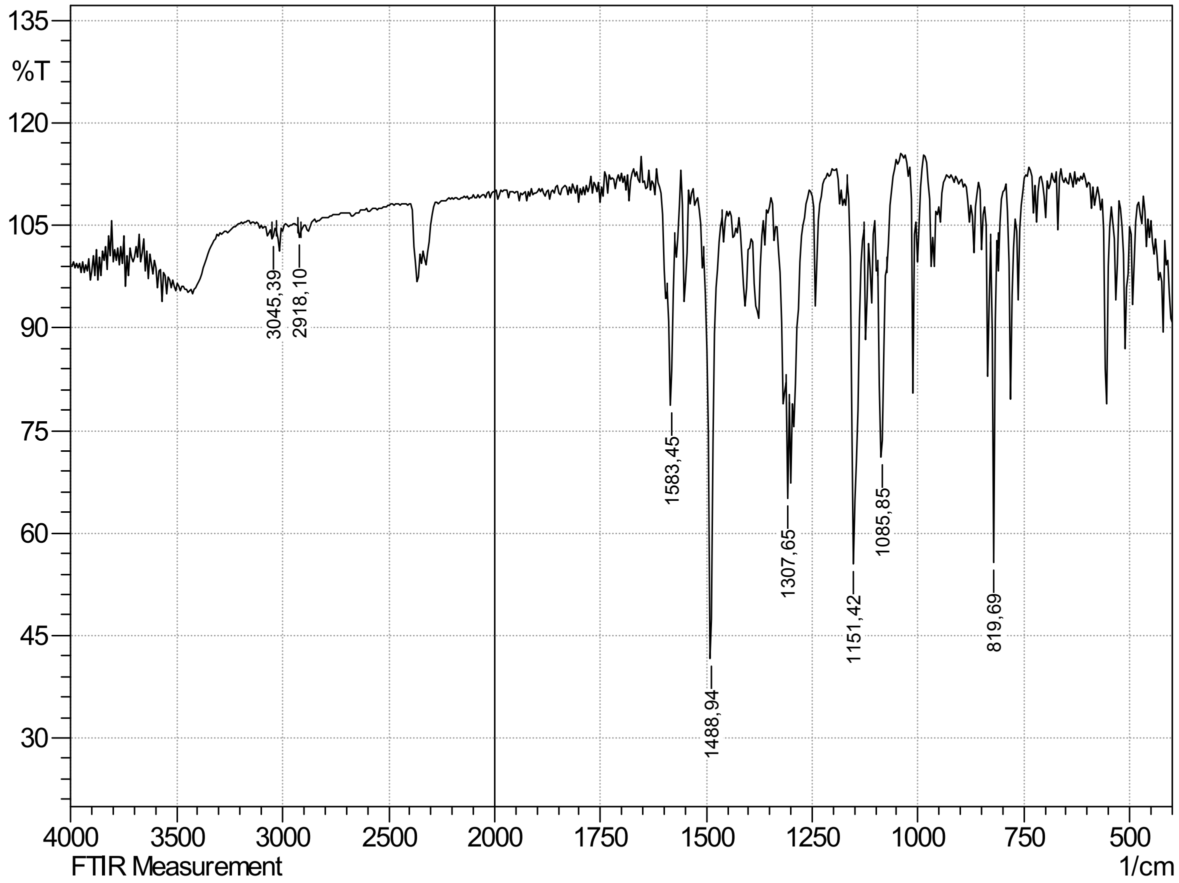

Figure S43. IR Spectrum of compound **2o**.

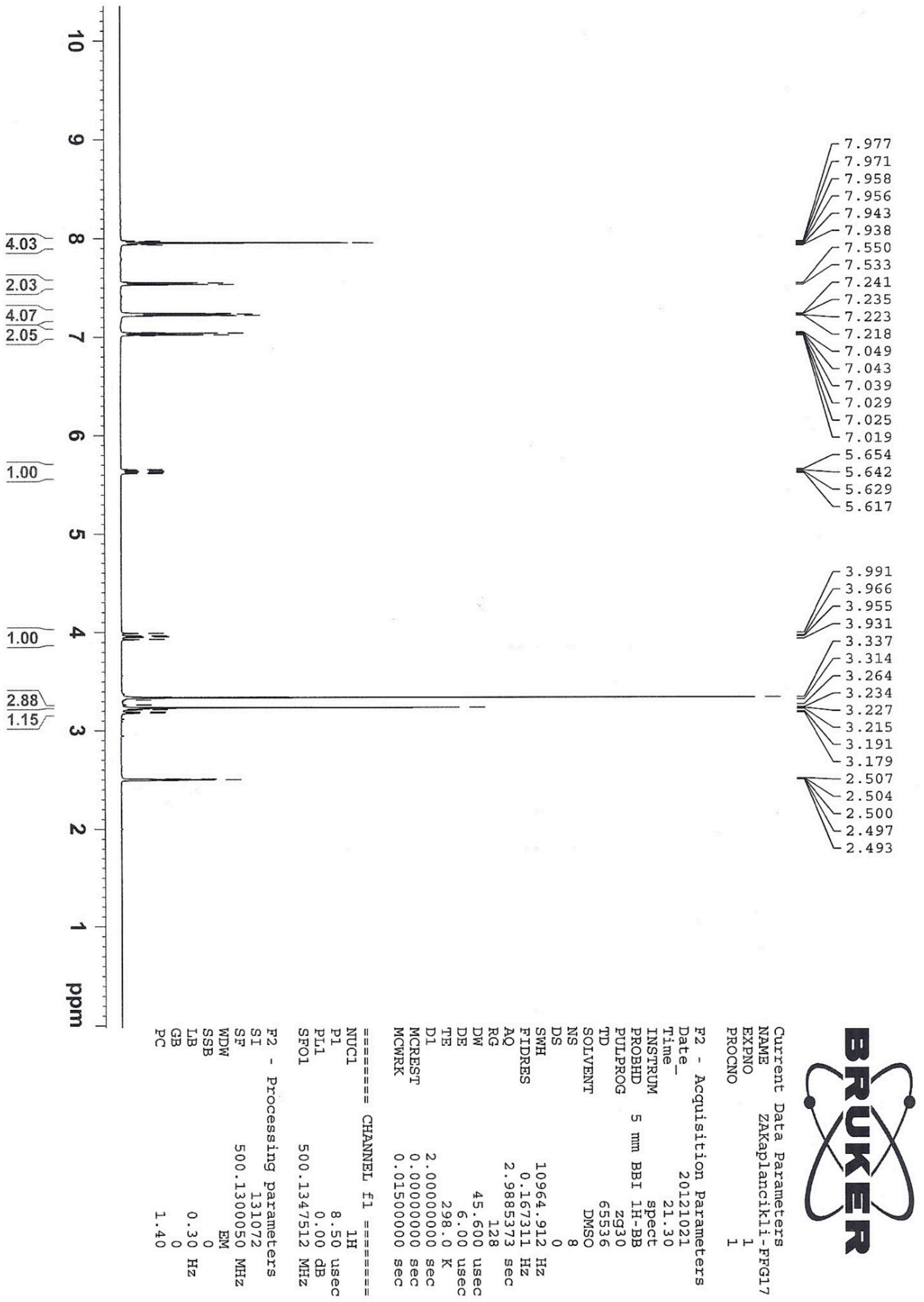

Figure S44. ^{1}H-NMR Spectrum of compound **2o**.

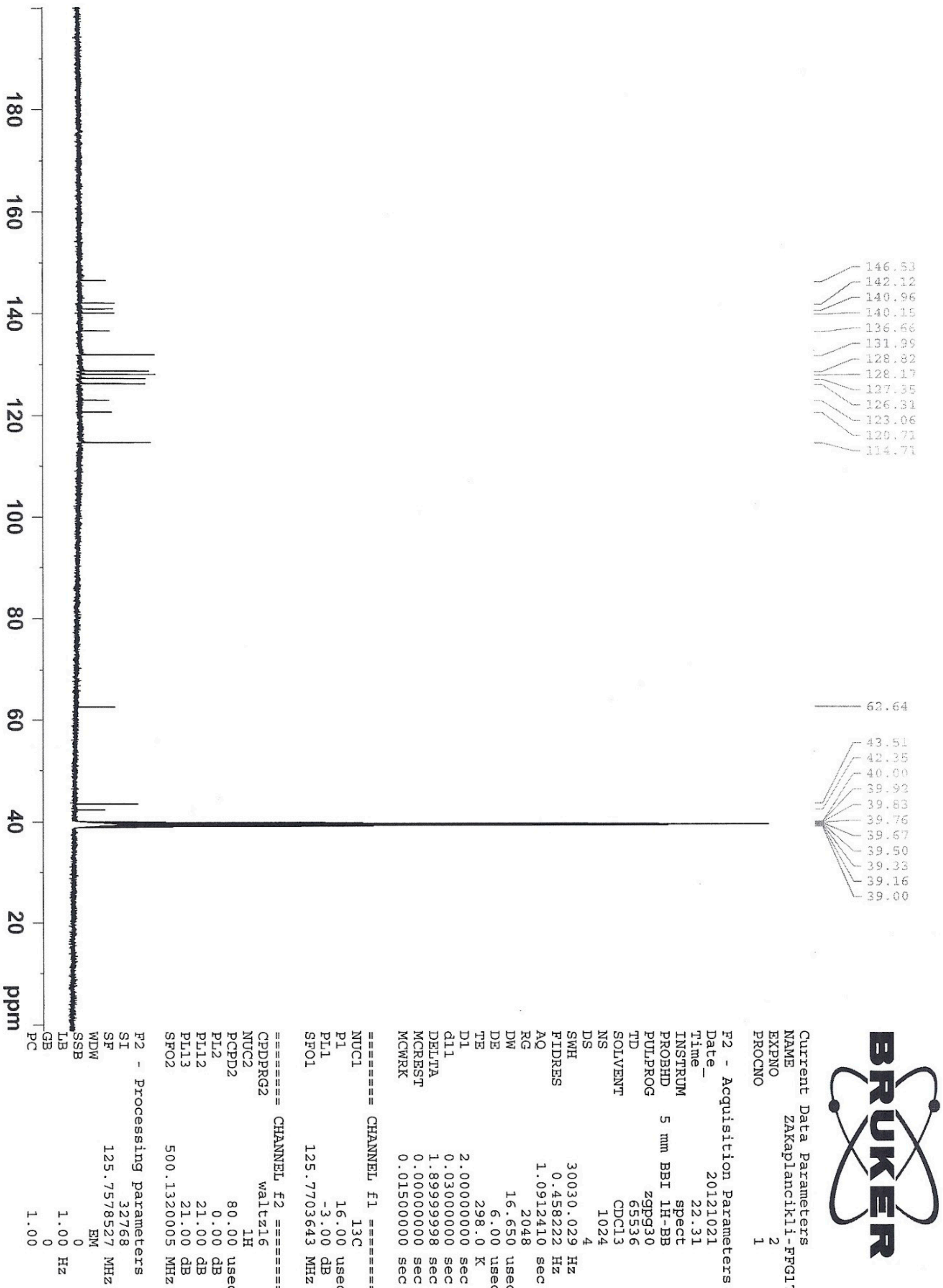

Figure S45. ^{13}C-NMR Spectrum of compound **2o**.

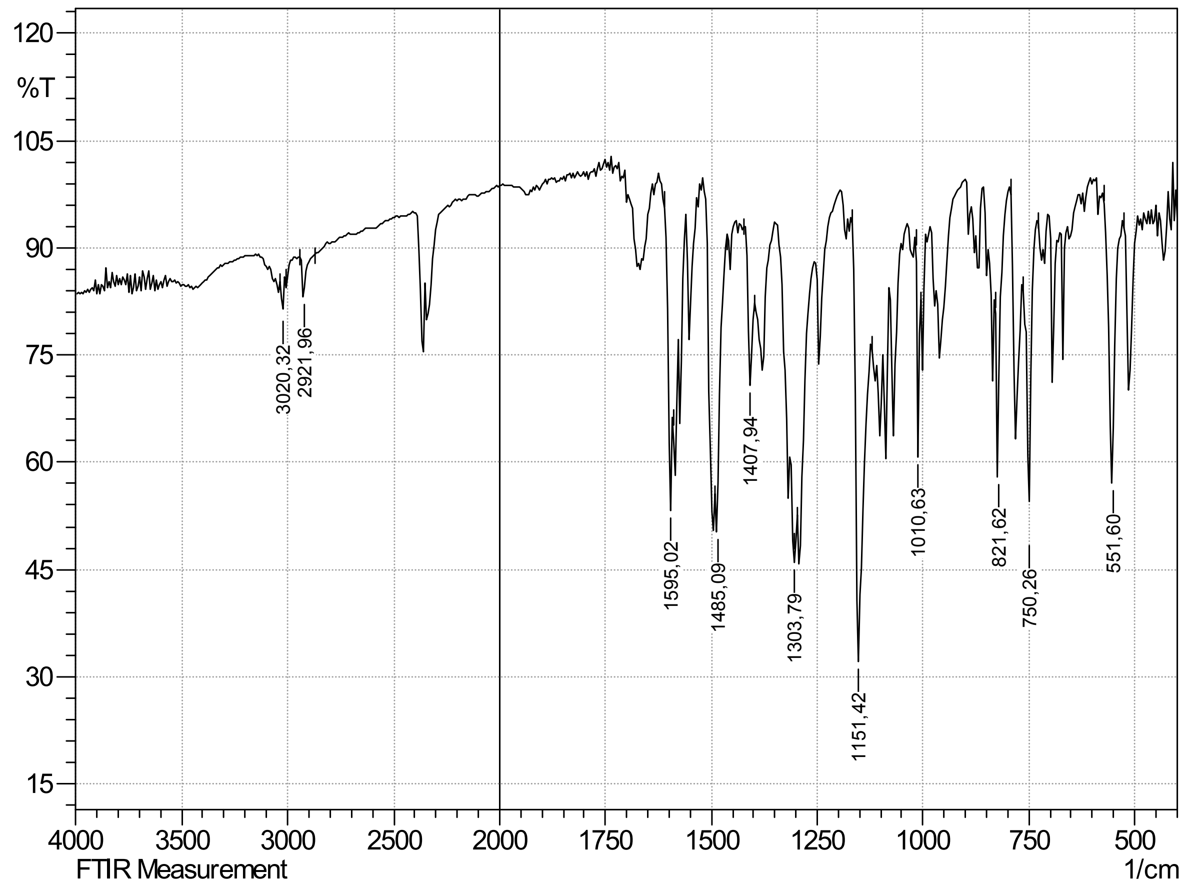

Figure S46. IR Spectrum of compound **2p**.

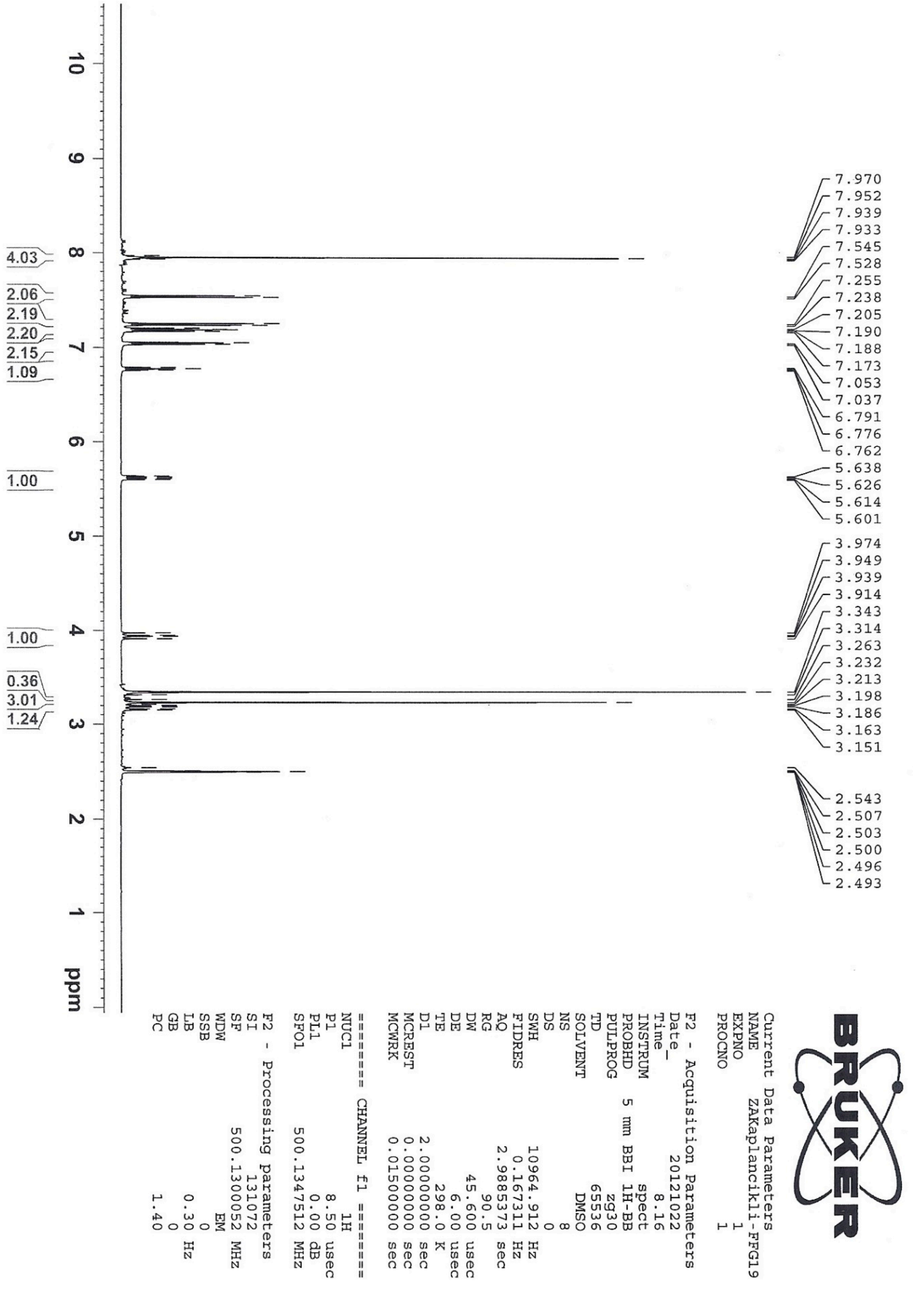

Figure S47. ^{1}H-NMR Spectrum of compound **2p**.

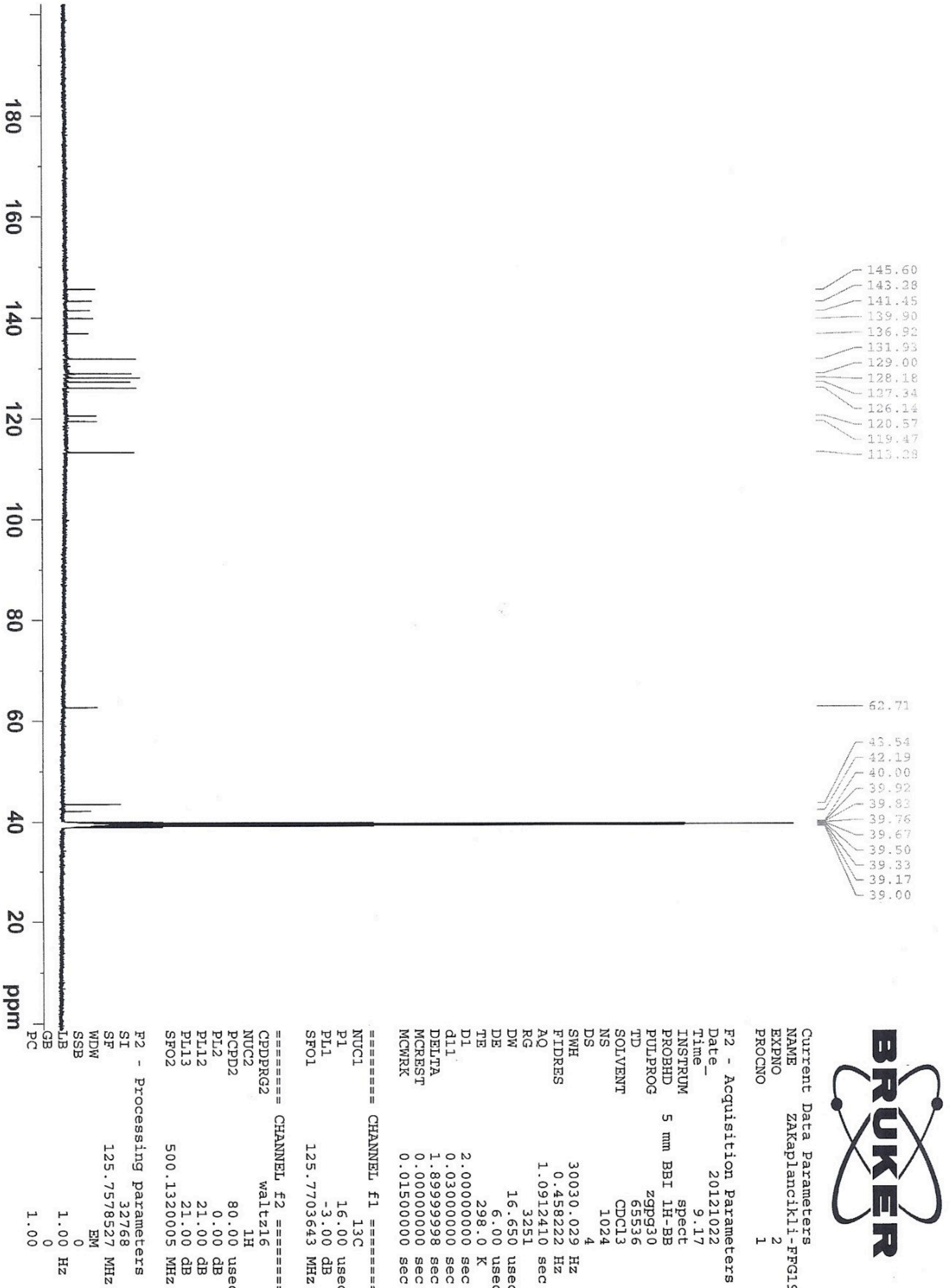

Figure S48. ^{13}C-NMR Spectrum of compound **2p**.

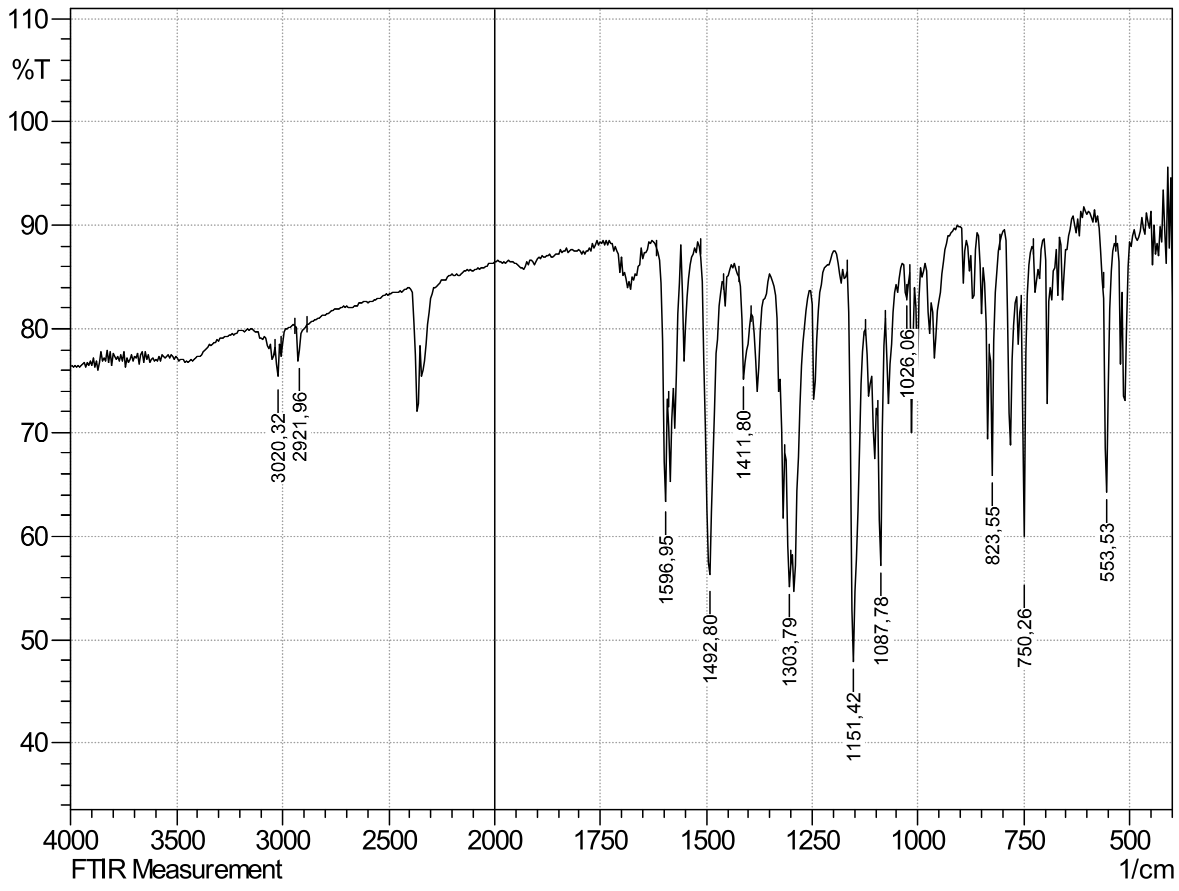

Figure S49. IR Spectrum of compound **2r**.

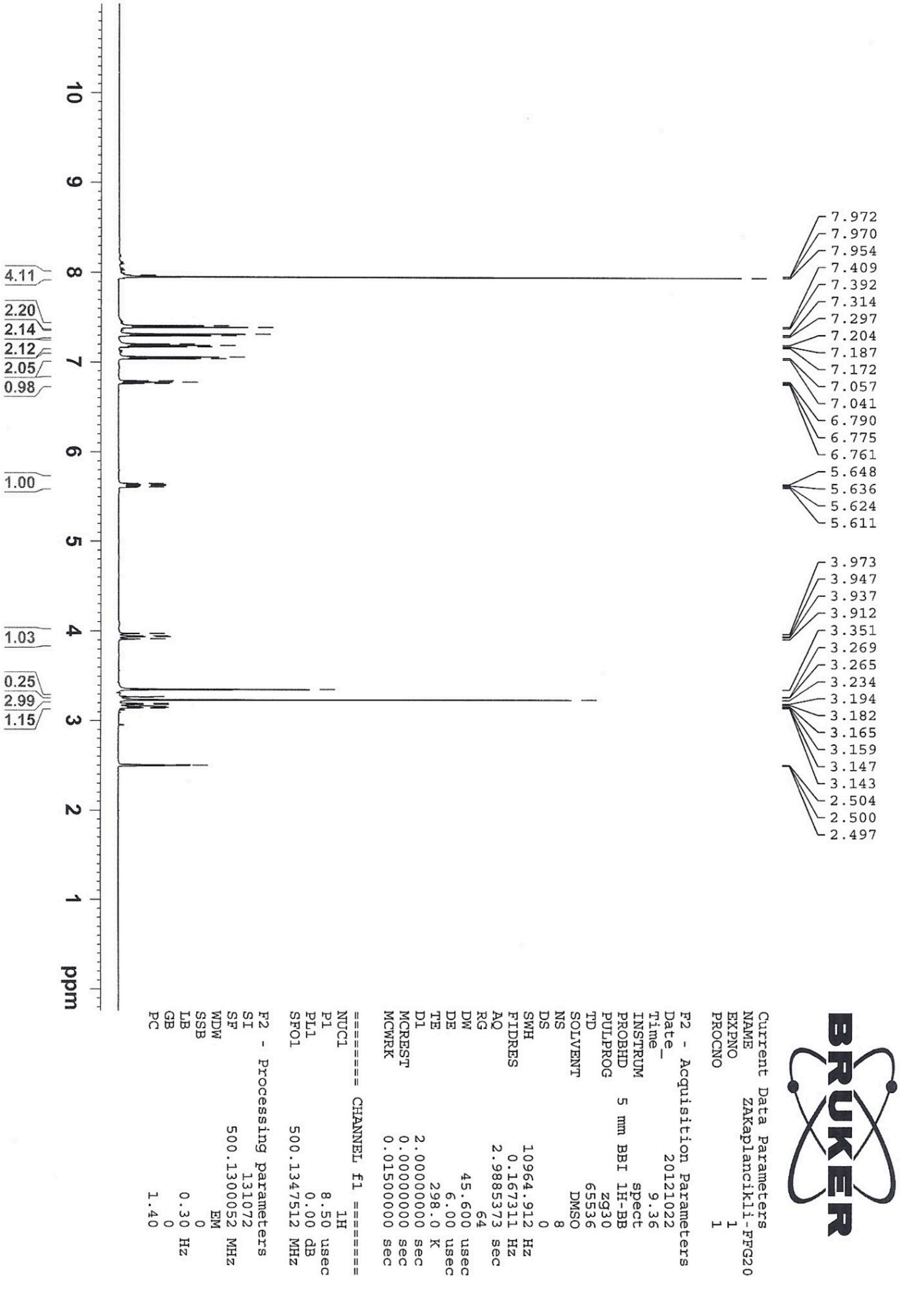

Figure S50. ^{1}H-NMR Spectrum of compound **2r**.

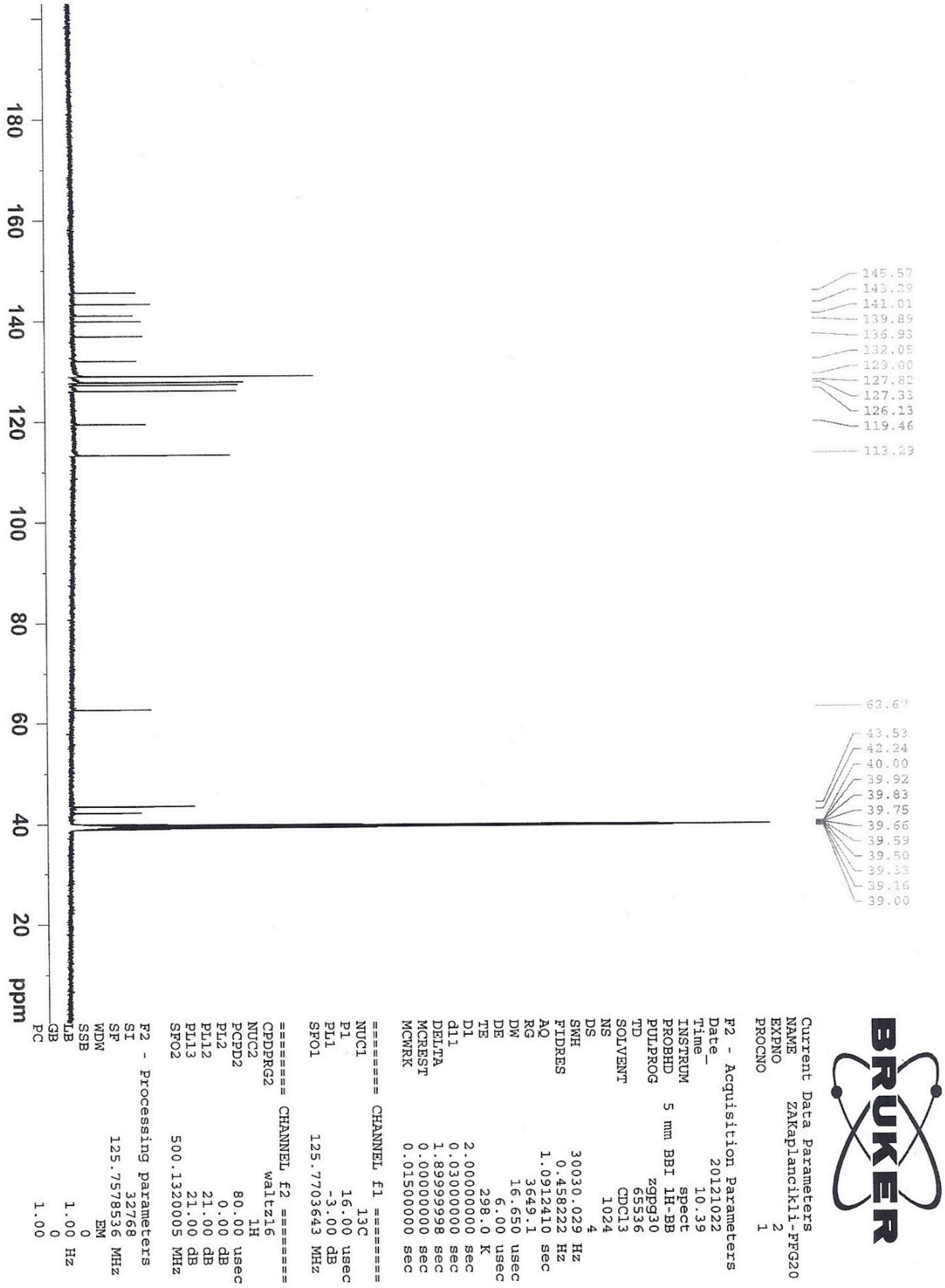

Figure S51. ^{13}C-NMR Spectrum of compound **2r**.

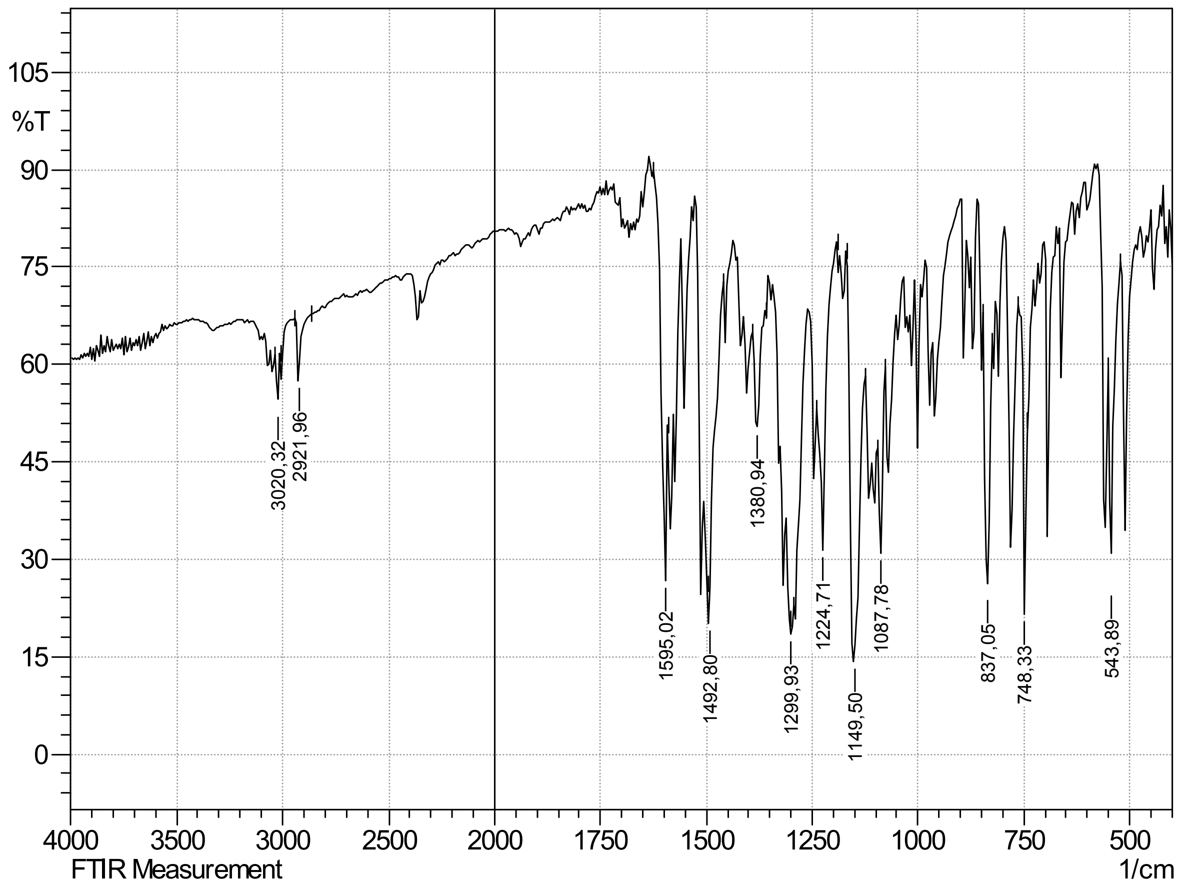

Figure S52. IR Spectrum of compound **2s**.

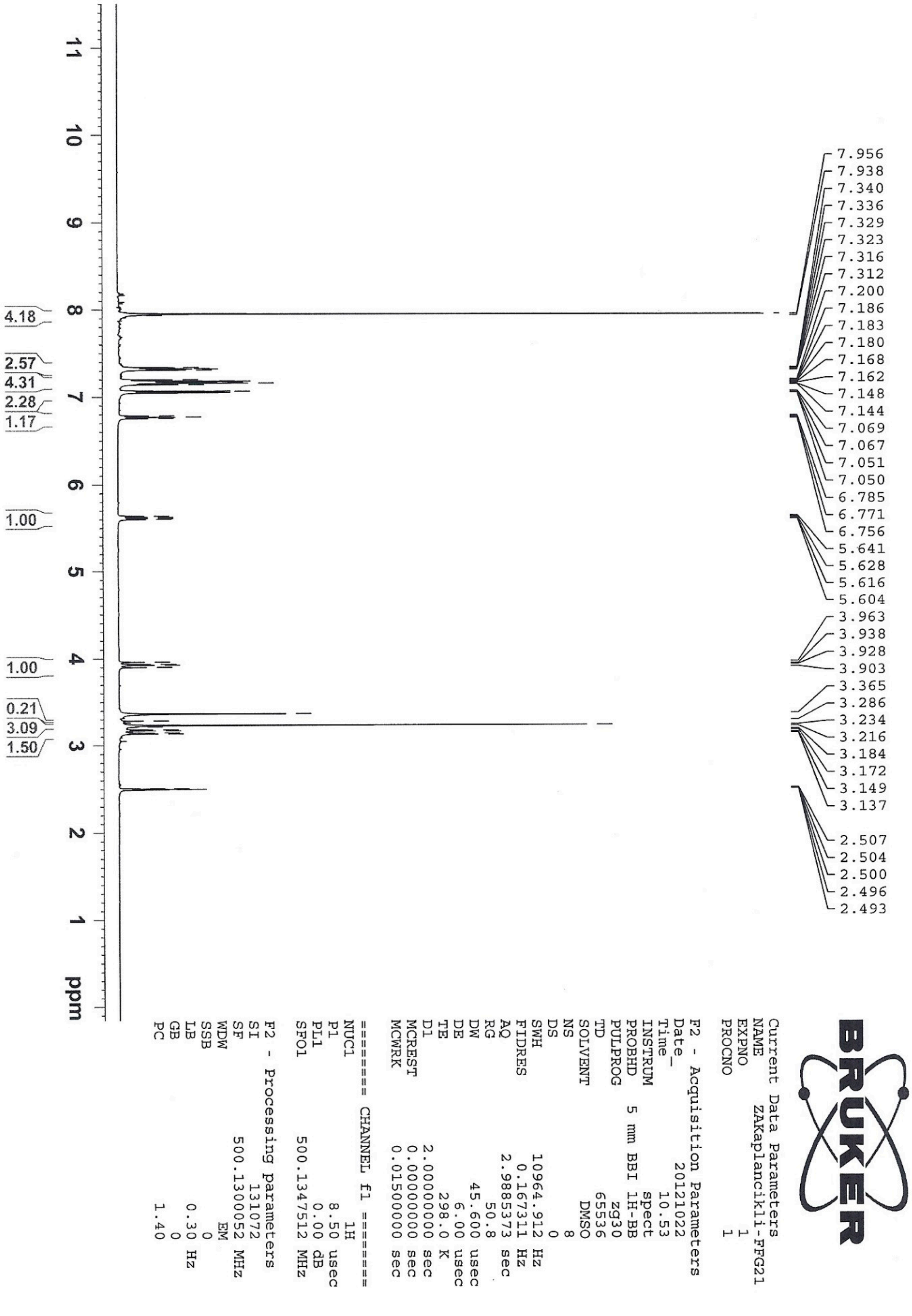

Figure S53. ^{1}H-NMR Spectrum of compound **2s**.

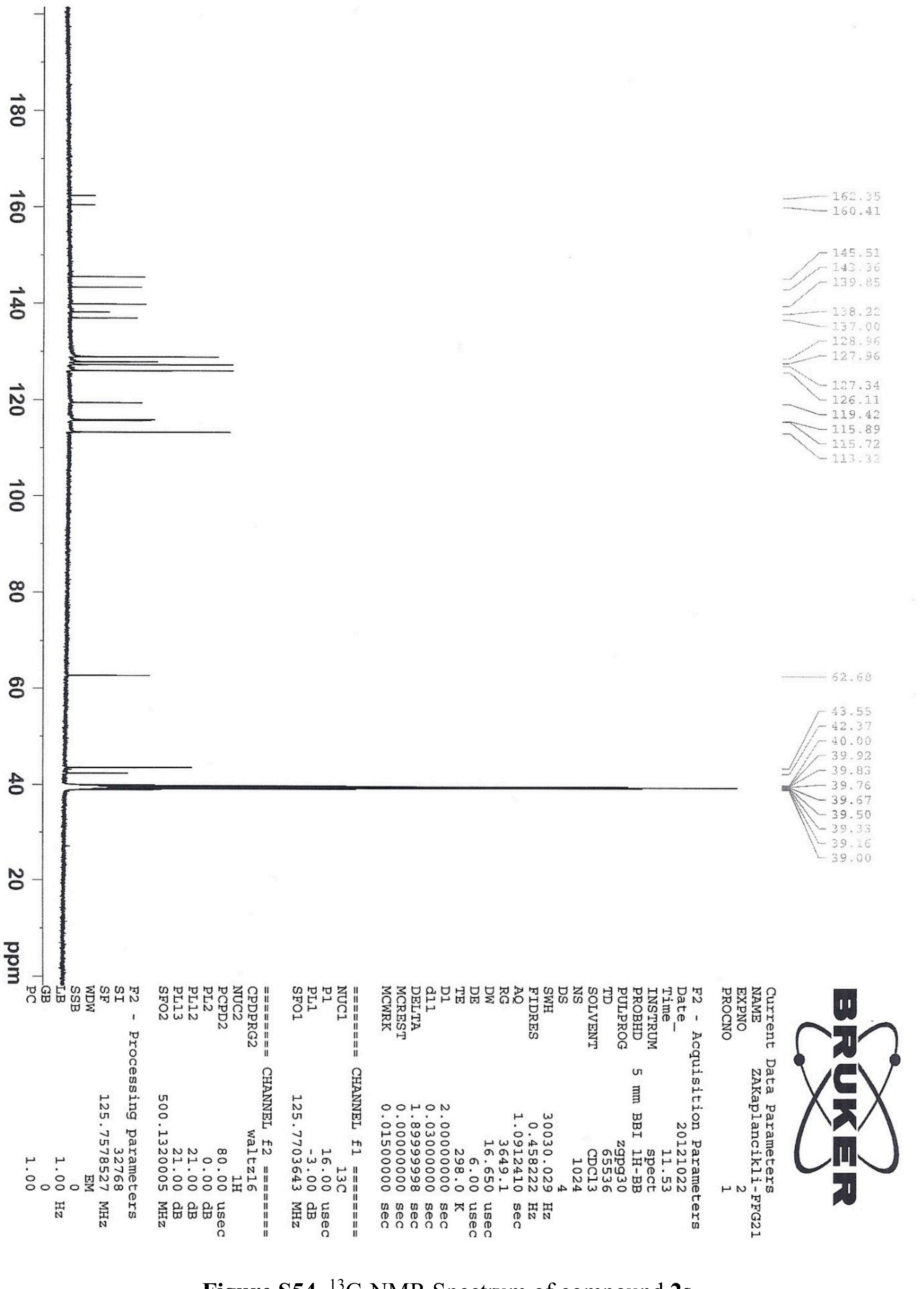

Figure S54. ^{13}C-NMR Spectrum of compound **2s**.
